# Theoretical and mathematical codynamics of nonlinear tuberculosis and COVID-19 model pertaining to fractional calculus and probabilistic approach

**DOI:** 10.1038/s41598-024-59261-7

**Published:** 2024-04-17

**Authors:** Saima Rashid, Sher Zaman Hamidi, Saima Akram, Muhammad Aon Raza, S. K. Elagan, Beida Mohsen Tami Alsubei

**Affiliations:** 1 Department of Mathematics, Government College University, Faisalabad, 38000 Pakistan; 2 Department of Computer Science and Mathematics, Lebanese American University, Beirut, 11022801 Lebanon; 3 Department of Physics, Nangarhar University, Jalalabad City, Nangarhar, 2601 Afghanistan; 4 Department of Mathematics, Government College Women University Faisalabad, Faisalabad, 38000 Pakistan; 5 Centre for Advanced Studies in Pure and Applied Mathematics, Bahauddin Zakariya, Multan, 60000 Pakistan; 6 Department of Mathematics and Statistics, College of Science, Taif University, P.O. Box 11099, Taif, 21944 Saudi Arabia; 7 Mathematics Program, Department of Science and Technology, Ranyah University College, Taif University, P.O. Box 11099, Taif, 21944 Saudi Arabia

**Keywords:** Co-infection TB and COVID-19 model, Deterministic and probabilistic model, Fractional calculus, Global positive solution, Ergodic stationary distribution, Probability density function, Quasi-equilibrium, Biochemistry, Diseases, Nanoscience and technology

## Abstract

Severe acute respiratory syndrome coronavirus 2 (SARS-CoV-2) is a novel virus known as coronavirus 2 (SARS-CoV-2) that affects the pulmonary structure and results in the coronavirus illness 2019 (COVID-19). Tuberculosis (TB) and COVID-19 codynamics have been documented in numerous nations. Understanding the complexities of codynamics is now critically necessary as a consequence. The aim of this research is to construct a co-infection model of TB and COVID-19 in the context of fractional calculus operators, white noise and probability density functions, employing a rigorous biological investigation. By exhibiting that the system possesses non-negative and bounded global outcomes, it is shown that the approach is both mathematically and biologically practicable. The required conditions are derived, guaranteeing the eradication of the infection. Sensitivity analysis and bifurcation of the submodel are also investigated with system parameters. Furthermore, existence and uniqueness results are established, and the configuration is tested for the existence of an ergodic stationary distribution. For discovering the system’s long-term behavior, a deterministic-probabilistic technique for modeling is designed and operated in MATLAB. By employing an extensive review, we hope that the previously mentioned approach improves and leads to mitigating the two diseases and their co-infections by examining a variety of behavioral trends, such as transitions to unpredictable procedures. In addition, the piecewise differential strategies are being outlined as having promising potential for scholars in a range of contexts because they empower them to include particular characteristics across multiple time frame phases. Such formulas can be strengthened via classical technique, power-law, exponential decay, generalized Mittag–Leffler kernels, probability density functions and random procedures. Furthermore, we get an accurate description of the probability density function encircling a quasi-equilibrium point if the effect of TB and COVID-19 minimizes the propagation of the codynamics. Consequently, scholars can obtain better outcomes when analyzing facts using random perturbations by implementing these strategies for challenging issues. Random perturbations in TB and COVID-19 co-infection are crucial in controlling the spread of an epidemic whenever the suggested circulation is steady and the amount of infection eliminated is closely correlated with the random perturbation level.

## Introduction

The COVID-19 outbreak has posed novel obstacles to worldwide medical systems, resulting in enormous impacts on nations around the globe. Undoubtedly, the battle against COVID-19 has taken up much of the attention, but it is important to remember that TB has existed as a problem for quite a while. Mankind has been plagued by this extremely contagious sickness for ages. Ultimately, 2020 will likely go down in history as the year that the coronavirus ailments, or COVID-19, took center stage. The outbreak’s causative agent, the SARS-CoV-2, first appeared in China in the second half of 2019^1,2^. Even though COVID-19 continues to be a topic widely discussed in academic journals and news reports, it’s crucial to remember about other infectious illnesses, such as TB^3,4^.

The COVID-19 outbreak has had a major effect on the TB treatment mechanism, resulting in a reduction in both detection and transmission. This is explained by the repercussions of TB care and limitations on accessibility for patients, which have led to an increase in TB-related mortality^5,6^. In order to successfully combat both of these transmissible illnesses, this viewpoint assessment seeks to point out the overlap between COVID-19 and TB, emphasizing their combined menace and suggesting common approaches.

**Figure 1:**
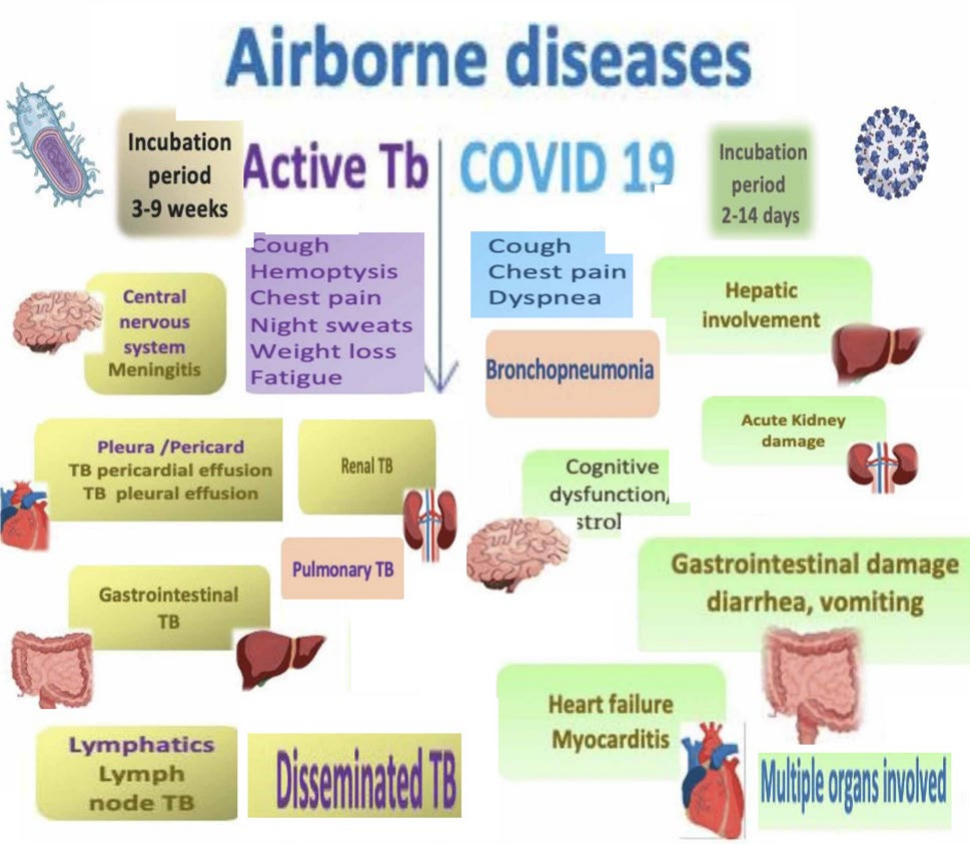
Identical indications and multi-organ connection to TB and COVID-19.

Furthermore, there are some notable clinical commonalities between the COVID-19 epidemic and TB. Since pulmonary secretions are an important way that these ailments are communicated, proximity and congested surroundings are favorable. Furthermore, COVID-19 and TB are especially dangerous for disadvantaged and underprivileged groups, such as the elderly, people with preexisting medical disorders, and people with compromised immunological capabilities. The COVID-19 epidemic has had a complex effect on TB. The increased challenge has caused a diversion to medical supplies, which has disrupted attempts to diagnose, address and regulate TB. Security measures, prohibitions on traveling and restricted availability of healthcare resources have made it more difficult to identify cases of TB and diagnose patients on time. The combination of these two contagious illnesses has produced a complicated scenario that needs prompt monitoring and all-encompassing approaches. Both COVID-19 and TB have a number of similarities, most notably the way in which their respective causal agents-*mycobacterium TB* and SARS-CoV-2-are transmitted^7^. Pulmonary system emissions are the route of transmission for both infections^8,9^. Both COVID-19 and TB can spread via aerosols and droppings, with the respiratory tract being their usual site of infection. It is crucial to remember, though, that such illnesses may impact a variety of organs^10^. In addition, finding and evaluating interactions as well as safeguarding medical personnel and individuals at risk are essential elements of healthcare safety for these illnesses. To create comprehensive and inexpensive prevention and treatment strategies, it is essential to comprehend the channels and components impacting propagation. Numerous decades of therapeutic and laboratory research on TB have yielded a plethora of data that can be used to identify, prioritize and evaluate exposures^8^. It should come as no surprise that more research is needed to better understand how SARS-CoV-2 spreads, and there is ongoing debate regarding the distinct functions played by airborne particles, microbes and big pulmonary secretions^11^. In particular, excessive growth occurrences have been linked to the propagation of these two diseases^12,13^. Figure 1 listed below shows a graphic that illustrates several of the prevalent therapeutic manifestations and multi-organ dysfunction.

**Figure 2:**
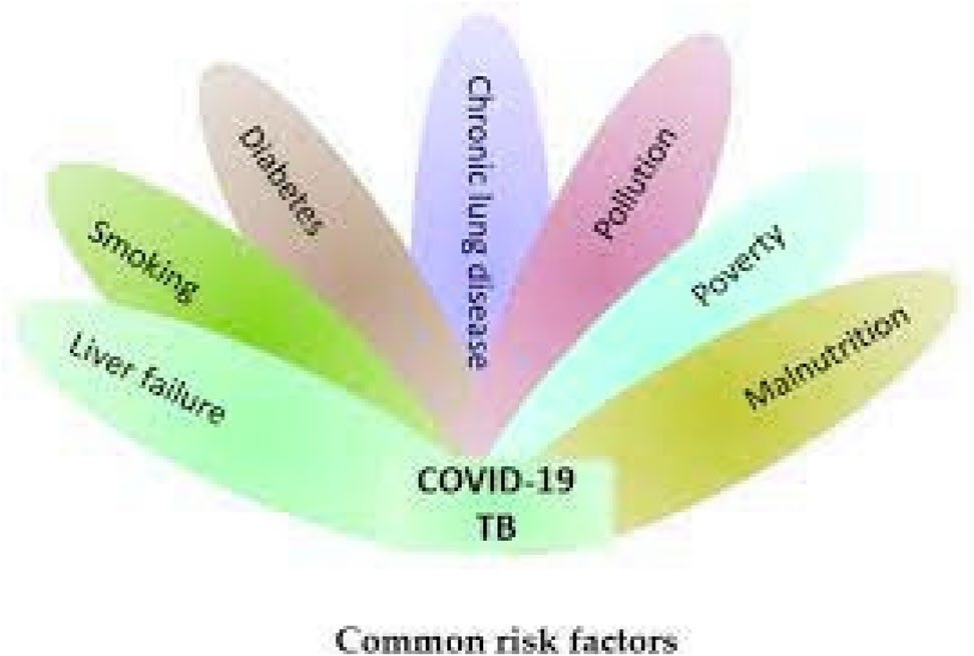
Basic danger signs for TB and COVID-19.

Whereas the implantation time for TB can range from 2 weeks to many decades until the TB infection advances, that of COVID-19 is less lengthy, ranging from 1 to 14 days. The manifestations of COVID-19 include anemia, wheezing, throat irritation, diminished or absent perception, flavor loss, vomiting, muscular soreness, and exhaustion. Usually, these indications start off suddenly. On the other hand, TB causes a high temperature, perspiration at night, a persistently persistent cough, bleeding in the cough, decreased hunger, heartburn, and exhaustion. On the other hand, TB manifestations appear gradually and with a subtle beginning. When it comes to COVID-19, those with coexisting illnesses, including HIV, insulin resistance, being overweight, persistent lung disease, persistent cardiac problems, and impaired immune systems, are more likely to have extreme symptoms. These inherent medical issues may exacerbate the advancement of the sickness. Conversely, concomitant conditions, including type II diabetes, sickle cell syndrome, severe obstructive pulmonary ailments, HIV, and a weakened immune system, are recognized to escalate the likelihood and intensity of TB transmission. Combating and curing serious forms of both COVID-19 and TB require an understanding of and commitment to controlling these coexisting conditions^14,15^. Figure 2 lists some of the prevalent danger indicators for both TB and COVID-19.

Meanwhile, the testing process facilities have been disrupted by COVID-19, resulting in decreased personnel objectives, longer evaluation processing times, and the unavailability of forensic equipment. The timely delivery of TB screening tests as well as their accessibility have been greatly impacted by these delays. Screening findings may take longer to reach people, which could postpone therapeutic beginnings and raise the danger of tuberculosis spreading throughout populations^16^. Screening TB infections and locating regions with widespread dissemination require efficient acquisition and inspection methods. The distribution of resources and focused treatments can be guided by observational reports. Effective use of statistical analysis and health monitoring networks can help with preventive choices and offer real-time information^17^.

During the years, a great deal of mathematical concepts have been developed to help us understand the world in which we live. In order to regulate presentations involving considerable obstacles, powerful artificial intelligence algorithms have been constructed, and the concept of space and time modeling has been put into practice. Some of the algorithmic techniques that are particularly commonly applied in modeling and prediction involve the idea of differentiation. Differential equations (DEs) are scientific techniques that have been created using this concept. In the beginning endeavor, researchers suggested a number of algorithms via multifaceted associations. The variation in the compositions could include local (exchange rate, conformable derivative, and fractal derivative)^18–20^; nonlocal/singular kernel (Riemann–Liouville, Liouville–Caputo, and multiple expressions)^21^; local/non-singular kernel (Caputo–Fabrizio operators)^22^; and finally non-local/non-singular (Atangana–Baleanu–Caputo operators)^23^. For a variety of interpretations of differential derivatives or the individuals who structured the foundations, a number of academics suggest numerous novel approaches. Fractional-order (FO) calculus has a connection to realistic endeavors and finds extensive application in multiple domains such as atomic physics, optics, image encryption, nanotechnology, and infectious disease^18–20,24^.

Recently, a subfield of mathematical physics and comprehension known as fractional calculus uses FO derivatives to study how inventions and documentation operate. FO modeling, as opposed to integer-order settings, can employ reminiscence memory of the power, exponential decay, or generalized Mittag–Leffler (GML) formation kernel to capture non-local spatial–temporal interactions. The conceivable benefits of using the fractional approach by Atangana–Baleanu involve all non-localities that are inherent within the explanation, just like in all previous variations. However, the most significant characteristic is that it has a nonsingular and non-local kernel, represented by the GML functionality, which, from an empirical viewpoint, includes the clarification and advancement of competencies delineated by a series of privileges. Kumar et al.^25^ contemplated a new investigation on fractional HBV models through Caputo and Atangana–Baleanu–Caputo derivatives. Mekkaoui et al.^26^ presented the predictor–corrector for non-linear DEs and integral equations with fractional operators. Atangana and Araz^27^ described a successive midpoint method for nonlinear DEs with integer and Caputo–Fabrizio derivatives. On the other hand, it has been shown that the previously mentioned approach precisely conveys the complex compositions of many practical representations^23,27^. The piecewise derivative, which has recently gained prominence^28^, was presented by Atangana and Araz^29^ and distinguishes from every derivative by the fact that it may reprise the interconnected paths that comprise these fractional algorithms in a differentiation technique. Every aspect that happens demonstrates that while the prevalence of the codynamics of COVID-19 and TB is probabilistic instead of deterministic in nature, knowledgeable research is based on an empirical methodology. A number of academics investigated the real-world growth of viruses and bacteria using the fundamental concept of probabilistic modeling, as reported in Refs.^30,31^.

Certain probabilistic COVID-19 individuals via TB concurrent infection outbreak frameworks using theropetic representatives hydroxychloroquine, azithromycin, lopinavir/ritonavir, and darunavir/cobicistat conjunction systems have been successfully defined to examine the influence of probabilistic white noise and offer several efficient initiatives for governing infection interactions. These frameworks are founded on the randomly generated linear disruptive methodology, which assumes the biological nature of ambient white noise correlates to the dimension of every compartment. Moreover, it has been demonstrated experimentally that a probabilistic COVID-19 and TB system that includes immunological dysfunction affected by inherent and adapted resistance can prevent an epidemic of co-infection. Motivated by these findings^32,33^, we also presume that the random perturbation is closely connected to specific populations of evolution of TB and COVID-19 diagnostics. In order to illustrate the significant influence of a probabilistic framework condition mentioned in Ref.^34^, we performed to create this paper. Additionally, we create a probabilistic mathematical structure utilizing piecewise fractional derivative expressions to analyze the co-infection process incorporating the positive immunomodulation against COVID-19, likely because of trained innate immunity and crossed heterologous immunity within predetermined time intervals. In order to achieve this, we separate the population into two groups: the incidence and occurrence of exacerbated immune dysregulation and decreased lymphocyte function, along with erroneous variations. The probability that the most recent COVID-19-infected TB will be engaged is represented by the proportion $\psi \in (0,1)$, whereas the unexplained component $1-\psi$ will not be implicated. In addition, we established the global positive solutions of the co-infection model with a unique ergodic and stationary distribution (ESD) technique to illustrate the biological properties and statistical viability of this structure. We also provide the precise definition of the probabilistic density function (P.D.F) at a quasi-equilibrium point that represents the probabilistic COVID-19 approach, which reflects significant spontaneous features in probabilistic relevance. The ESD and P.D.F surrounding the quasi-equilibrium point of the randomized multidimensional codynamics framework will be better understood as a result of this investigation. The intention of the investigation is to acquire an improved comprehension of how the infection persists over time in the probabilistic codynamics system. In general, fractional operators examine simulations conducted numerically of the proposed system that include crossover structures and white noise.

## Codynamics model and preliminaries

The general population is divided into eight indistinguishable groups in this category, which are designated as susceptible people, $({\textbf{S}})$, latent TB patients who do not exhibit TB-associated indications and are not pathogenic ${{\textbf{L}}}_{{\textbf{T}}}$, influential TB-infected people ${{\textbf{I}}}_{{\textbf{T}}}$, COVID-19-infested humans who do not exhibit indications but are transmissible ${{\textbf{E}}}_{{\textbf{C}}}$, COVID-19-diagnosed people who exhibit scientific backing indications and are pathogenic ${{\textbf{I}}}_{{\textbf{C}}}$, both inactive TB and COVID-19-contaminated people ${{\textbf{L}}}_{{\textbf{T}}{\textbf{C}}}$, current TB and COVID-19-contaminated humans ${{\textbf{I}}}_{{\textbf{T}}{\textbf{C}}}$, and retrieved people ${{\textbf{R}}}$ consisting of both TB and COVID-19. The underlying computational framework for the codynamics of TB and COVID-19 is developed in this portion. Considering such, all people at moment $\tau$, represented by ${\textbf{N}}(\tau )$, are provided by

1 $$\begin{aligned} {\textbf{N}}(\tau )={\textbf{S}}(\tau )+{{\textbf{L}}}_{{\textbf{T}}}(\tau ) +{{\textbf{I}}}_{{\textbf{T}}}(\tau )+{{\textbf{E}}}_{{\textbf{C}}}(\tau )+{{\textbf{I}}}_{{\textbf{C}}}(\tau ) +{{\textbf{L}}}_{{\textbf{T}}{\textbf{C}}}(\tau )+{{\textbf{I}}}_{{\textbf{T}}{\textbf{C}}}(\tau )+{{\textbf{R}}}(\tau ). \end{aligned}$$

We hypothesized that acquisition increases the vulnerable community at an intensity of $\nabla$. Every person in every compartment experiences an inevitable mortality rate of $\beta$. Equivalent to formula (1), vulnerable individuals contract TB via interaction with current TB individuals via agent transmission $\psi _{{\textbf{T}}}$. The acceptable interaction rate for TB transmission is indicated by $\alpha _{1}$ within this manifestation. It is believed that people with persistent TB are undiagnosed and cannot pass on the illness^35^. Likewise, those at risk contract COVID-19 at an intensity of transmission $\psi _{{\textbf{C}}}$, which is determined as in formula (1), after effectively coming into proximity to COVID-19-infected people. The efficient interaction probability for COVID-19 infection is represented by $\alpha _{2}$ in this case. Furthermore, we hypothesized that people in the hidden TB segment ${{\textbf{L}}}_{{\textbf{T}}}$ depart at an incidence of $\mu$ to segment ${{\textbf{I}}}_{{\textbf{T}}}$, at an incidence of transmission of $\lambda \psi _{{\textbf{C}}}$ to the persistent TB as well as COVID-19 contaminated group, whilst certain of them recuperate at an intervention incidence of $\varpi$. Additionally, those in the TB-infected category ${{\textbf{I}}}_{{\textbf{T}}}$ recuperate due to the illness at an incidence of $\delta$, with the surviving percentage either transferring to the transmission category ${{\textbf{I}}}_{{\textbf{T}}{\textbf{C}}}$ at a pace of $\varsigma _{3}$ or dying at a speed of $\zeta _{{\textbf{T}}}$ via TB-induced mortality.

**Figure 3:**
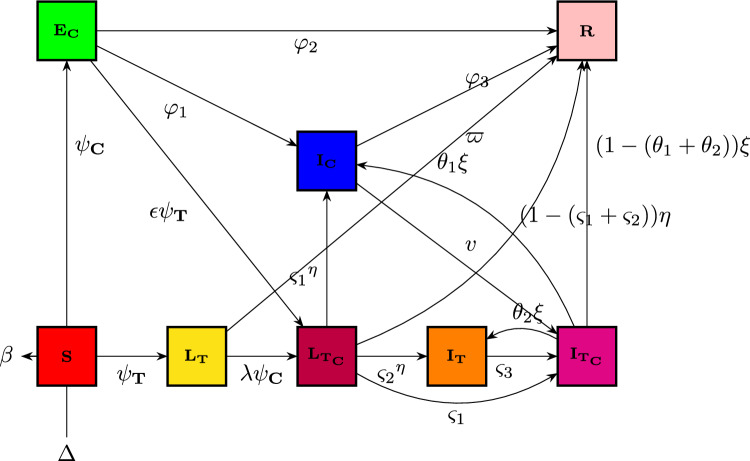
Flow diagram for depicting the codynamics process of TB-COVID-19 model (2).

The overall community in cohort ${{\textbf{L}}}_{{\textbf{T}}{\textbf{C}}}$ potentially dies at COVID-19-induced mortality pace $\zeta _{{\textbf{C}}}$ or advances at an intensity of $\rho$ to become contaminated category ${{\textbf{I}}}_{{\textbf{T}}{\textbf{C}}}$. As seen in Fig. 3, it is believed that the other people will be moved to the other cohort at a consistent multiplicity of $\eta$. In other words, the general population classified as ${{\textbf{L}}}_{{\textbf{T}}{\textbf{C}}}$ migrates at an intensity of $\varsigma _{2}\eta$ to category ${{\textbf{I}}}_{{\textbf{T}}}$, then at a pace of $\varsigma _{1}\eta$ to compartment ${{\textbf{I}}}_{{\textbf{C}}}$ group, and finally recovers at a pace of $(1-(\varsigma _{1}+\varsigma _{2}))\eta$. Additionally, we hypothesized that, although the codynamics-induced mortality prevalence is represented by $\zeta _{{\textbf{T}}{\textbf{C}}}$, people in compartment ${{\textbf{I}}}_{{\textbf{T}}{\textbf{C}}}$ depart for compartments ${{\textbf{I}}}_{{\textbf{T}}},~{{\textbf{I}}}_{{\textbf{C}}}$ or ${\textbf{R}}$, correspondingly, at an intensity of $\theta _{2}\xi ,~\theta _{1}\xi$ or $(1-(\theta _{1}+\theta _{2}))\xi .$ Furthermore, at an intensity of $\epsilon \psi _{{\textbf{T}}},\phi$ or $\varphi _{2},$ the COVID-19 exposure people ${{\textbf{E}}}_{{\textbf{C}}}$ can choose to depart to compartment ${{\textbf{L}}}_{{\textbf{T}}{\textbf{C}}},~{{\textbf{I}}}_{{\textbf{C}}}$ or ${\textbf{R}}$, respectively. Comparably, the number of individuals in compartment ${{\textbf{I}}}_{{\textbf{C}}}$ is either moved to the codynamics cohort at an intensity of $\nu$ or restored at a steady pace of $\varphi _{3}$. $\zeta _{{\textbf{C}}}$ represents the disease-induced fatality rate within this category. Figure 3 displays the suggested system’s process layout.

It leads to frameworks for the subsequent nonlinear DEs determined by the procedure illustration:

2 $$\begin{aligned} {\left\{ \begin{array}{ll} \dot{{\textbf{S}}}=\nabla -(\psi _{{\textbf{T}}}+\psi _{{\textbf{C}}}+\beta ){\textbf{S}},\\ \dot{{{\textbf{L}}}_{{\textbf{T}}}}=\psi _{{\textbf{T}}}{\textbf{S}}-(\beta +\mu +\lambda \psi _{{\textbf{C}}}+\varpi ){{\textbf{L}}}_{{\textbf{T}}},\\ \dot{{{\textbf{I}}}_{{\textbf{T}}}}=\mu {{\textbf{L}}}_{{\textbf{T}}}+\varsigma _{2}\eta {{\textbf{L}}}_{{\textbf{T}}{\textbf{C}}}+\theta _{2}\xi {{\textbf{I}}}_{{\textbf{T}}{\textbf{C}}}-(\beta +\varsigma _{3}+\zeta _{{\textbf{T}}}+\delta ){{\textbf{I}}}_{{\textbf{T}}},\\ \dot{{{\textbf{E}}}_{{\textbf{C}}}}=\psi _{{\textbf{C}}}{\textbf{S}}-(\beta +\epsilon \psi _{{\textbf{T}}}+\varphi _{1}+\varphi _{2}){{\textbf{E}}}_{{\textbf{C}}},~~~~~~~~~~~~~~~~~~~~~~~~~~~~~~~0\le \tau \le \intercal _{1},\\ \dot{{{\textbf{I}}}_{{\textbf{C}}}}=\varphi _{1} {{\textbf{E}}}_{{\textbf{C}}}+\rho \eta {{\textbf{L}}}_{{\textbf{T}}{\textbf{C}}}+\theta _{1}\xi {{\textbf{I}}}_{{\textbf{T}}{\textbf{C}}}-(\beta +\zeta _{{\textbf{C}}}+\nu +\varphi _{3}){{\textbf{I}}}_{{\textbf{C}}},\\ \dot{{{\textbf{L}}}_{{\textbf{T}}{\textbf{C}}}}=\lambda \psi _{{\textbf{C}}}{{\textbf{L}}}_{{\textbf{T}}}+\epsilon \psi _{{\textbf{T}}}{{\textbf{E}}}_{{\textbf{C}}}-(\beta +\zeta _{{\textbf{C}}}+\rho +\eta ){{\textbf{L}}}_{{\textbf{T}}{\textbf{C}}},\\ \dot{{{\textbf{I}}}_{{\textbf{T}}{\textbf{C}}}}=\rho {{\textbf{L}}}_{{\textbf{T}}{\textbf{C}}}+\varsigma _{3}{{\textbf{I}}}_{{\textbf{T}}}+\nu {{\textbf{I}}}_{{\textbf{C}}}-(\beta +\zeta _{{\textbf{T}}{\textbf{C}}}+\xi ){{\textbf{I}}}_{{\textbf{T}}{\textbf{C}}},\\ \dot{{{\textbf{R}}}}=\varpi {{\textbf{L}}}_{{\textbf{T}}}+\varphi _{2}{{\textbf{E}}}_{{\textbf{C}}}+\delta {{\textbf{I}}}_{{\textbf{T}}}+\varphi _{3}{{\textbf{I}}}_{{\textbf{C}}}+(1-(\varsigma _{1}+\varsigma _{2}))\eta {{\textbf{L}}}_{{\textbf{T}}{\textbf{C}}}+(1-(\theta _{1}+\theta _{2}))\xi {{\textbf{I}}}_{{\textbf{T}}{\textbf{C}}}-\beta {\textbf{R}},\end{array}\right. } \end{aligned}$$

where $\psi _{{\textbf{T}}}=\frac{\alpha _{1}}{{\textbf{N}}(\tau )}\big ({{\textbf{I}}}_{{\textbf{T}}}(\tau )+{{\textbf{I}}}_{{\textbf{T}}{\textbf{C}}}(\tau )\big )$ and $\psi _{{\textbf{C}}}=\frac{\alpha _{2}}{{\textbf{N}}(\tau )}\big ({{\textbf{E}}}_{{\textbf{C}}}(\tau )+{{\textbf{I}}}_{{\textbf{C}}}(\tau )+{{\textbf{L}}}_{{\textbf{T}}{\textbf{C}}}(\tau )+{{\textbf{I}}}_{{\textbf{T}}{\textbf{C}}}(\tau )\big ),$ containing positive initial conditions (ICs) ${{\textbf{S}}}(0)\ge 0,~{{\textbf{L}}}_{{\textbf{T}}}\ge 0,~{{\textbf{I}}}_{{\textbf{T}}}\ge 0,~{{\textbf{E}}}_{{\textbf{C}}}\ge 0,~{{\textbf{I}}}_{{\textbf{C}}}\ge 0,~{{\textbf{L}}}_{{\textbf{T}}{\textbf{C}}}\ge 0,~{{\textbf{I}}}_{{\textbf{T}}{\textbf{C}}}\ge 0,~{{\textbf{R}}}\ge 0.$

**Table 1:** Description of model’s parameters.

Symbols	Description
$\beta$	People’ spontaneous mortality rate
$\rho$	Rate of transmission of COVID-19 andTB exposure within the contaminated group
$\varphi _{1}$	Transmission rate of infection among those inoculated to COVID-19
$\varphi _{3}$	Probability of recuperation for a COVID-19 influenced person
$\epsilon$	Percentage of TB exposure in people subjected to COVID-19
$\varpi$	Recuperation percentage of inactive TB infections
$\nu$	Percentage of COVID-19 afflicted people who have TB disease
$\varsigma _{3}$	COVID-19 contamination incidence among TB patients
$\varphi _{2}$	Probability of recuperation for those subjected to COVID-19
$\eta$	Probability at which people exit the ${{\textbf{L}}}_{{\textbf{T}}{\textbf{C}}}$ group
$\varsigma _{1}$	TB healing rate for ${{\textbf{L}}}_{{\textbf{T}}{\textbf{C}}}$ of long-term care residents
$\varsigma _{2}$	Percentage of ${{\textbf{L}}}_{{\textbf{T}}{\textbf{C}}}$ patients recuperating with COVID-19
$\xi$	Proportion at which people quit the affected group ${{\textbf{I}}}_{{\textbf{T}}{\textbf{C}}}$
$\theta _{1}$	${{\textbf{I}}}_{{\textbf{T}}{\textbf{C}}}$ patients’ rate of TB recurrence
$\theta _{2}$	COVID-19 recuperation percentage among ${{\textbf{I}}}_{{\textbf{T}}{\textbf{C}}}$ participants
$\lambda$	Percentage of people suffering from TB who also get COVID-19
$\delta$	Recoverability percentage of TB patients
$\zeta _{{\textbf{T}}}$	Mortality rate from TB
$\zeta _{{\textbf{C}}}$	Mortality rate from COVID-19 infection
$\zeta _{{\textbf{T}}{\textbf{C}}}$	Mortality incidence as a result of both infections co-occurring
$\nabla$	Recruiting rate for those who are vulnerable
$\alpha _{1}$	Prevalence of TB infection propagation
$\alpha _{2}$	Rate of COVID-19 propagation
$\mu$	Percentage of people confronted with TB who get the disease
$\psi _{{\textbf{T}}}$	Intensity transmission for TB (the likelihood of contracting a virus from a TB sick person)
$\psi _{{\textbf{C}}}$	COVID-19 intensity of illness: the likelihood of contracting the virus from a person who has COVID-19 illness

Table 1 provides a description of the system’s characteristics.

To help readers that are acquainted with fractional calculus, we provide the related summary herein (see^21–23^ comprehensive discussion on fractional calculus).

$$\begin{aligned} \,_{0}^{c}{\textbf{D}}_{\tau }^{\omega } {\mathcal {G}}(\tau )=\frac{1}{\Gamma (1-\omega )}\int \limits _{0}^{\tau }{\mathcal {G}}^{\prime }({\textbf{q}})(\tau -{\textbf{q}})^{\omega }d{\textbf{q}},~~\omega \in (0,1]. \end{aligned}$$

The index kernel is involved in the Caputo fractional derivative (CFD). Whenever experimenting with a particular integral transform, such as the Laplace transform^36,37^, the CFD accommodates regular ICs.

$$\begin{aligned} \,_{0}^{CF}{\textbf{D}}_{\tau }^{\omega } {\mathcal {G}}(\tau )=\frac{\bar{{\mathcal {M}}}(\omega )}{1-\omega }\int \limits _{0}^{\tau }{\mathcal {G}}^{\prime }({\textbf{q}})\exp \bigg [-\frac{\omega }{1-\omega }(\tau -{\textbf{q}})\bigg ]d{\textbf{q}},~~\omega \in (0,1], \end{aligned}$$

where $\bar{{\mathcal {M}}}(\omega )$ indicates the normalization function $\bar{{\mathcal {M}}}(0)=\bar{{\mathcal {M}}}(1)=1.$

The non-singular kernel of the Caputo-Fabrizio fractional derivative (CFFD) operator has drawn the attention of numerous researchers. Furthermore, representing an assortment of prevalent issues that obey the exponential decay memory is best suited to utilize the CFFD operator^38^. With the passage of time, developing a mathematical model using the CFFD became a remarkable field of research. In recent times, several mathematicians have been busy with the development and simulation of CFFD DEs^39^.

The ABC fractional derivative operator is described as follows:

$$\begin{aligned} \,_{0}^{ABC}{\textbf{D}}_{\tau }^{\omega } {\mathcal {G}}(\tau )=\frac{ABC(\omega )}{1-\omega }\int \limits _{0}^{\tau }{\mathcal {G}}^{\prime }({\textbf{q}})E_{\omega }\bigg [-\frac{\omega }{1-\omega }(\tau -{\textbf{q}})^{\omega }\bigg ]d{\textbf{q}},~~\omega \in (0,1], \end{aligned}$$

where $ABC(\omega )=1-\omega +\frac{\omega }{\Gamma (\omega )}$ represents the normalization function.

The memory utilized in Atangana–Baleanu–Caputo fractional derivative (ABCFD) can be found intuitively within the index-law analogous for an extended period as well as exponential decay in a number of scientific concerns^40,41^. The broad scope of the connection and the non-power-law nature of the underlying tendency are the driving forces behind the selection of this version. The impact of the kernel, considered crucial in the dynamic Baggs–Freedman framework, was fully produced by the GML function^42^.

To far better perceive the propagation of TB and COVID-19, we indicate a dynamic mechanism (2) that includes the co-infection within the context of CFD, CFFD and ABCFD, respectively. This is because FO algorithms possess inherited properties that characterize the local/non-local and singular/non-singular dynamics of natural phenomena, presented as follows:

3 $$\begin{aligned} {\left\{ \begin{array}{ll} \,^{c}{\textbf{D}}_{\tau }^{\omega }{\textbf{S}}=\nabla -(\psi _{{\textbf{T}}}+\psi _{{\textbf{C}}}+\beta ){\textbf{S}},\\ \,^{c}{\textbf{D}}_{\tau }^{\omega }{{\textbf{L}}}_{{\textbf{T}}}=\psi _{{\textbf{T}}}{\textbf{S}}-(\beta +\mu +\lambda \psi _{{\textbf{C}}}+\varpi ){{\textbf{L}}}_{{\textbf{T}}},\\ \,^{c}{\textbf{D}}_{\tau }^{\omega }{{\textbf{I}}}_{{\textbf{T}}}=\mu {{\textbf{L}}}_{{\textbf{T}}}+\varsigma _{2}\eta {{\textbf{L}}}_{{\textbf{T}}{\textbf{C}}}+\theta _{2}\xi {{\textbf{I}}}_{{\textbf{T}}{\textbf{C}}}-(\beta +\varsigma _{3}+\zeta _{{\textbf{T}}}+\delta ){{\textbf{I}}}_{{\textbf{T}}},\\ \,^{c}{\textbf{D}}_{\tau }^{\omega }{{\textbf{E}}}_{{\textbf{C}}}=\psi _{{\textbf{C}}}{\textbf{S}}-(\beta +\epsilon \psi _{{\textbf{T}}}+\varphi _{1}+\varphi _{2}){{\textbf{E}}}_{{\textbf{C}}},~~~~~~~~~~~~~~~~~~~~~~~~~~~~~~~~~~~~~~~\intercal _{1}\le \tau \le \intercal _{2},\\ \,^{c}{\textbf{D}}_{\tau }^{\omega }{{\textbf{I}}}_{{\textbf{C}}}=\varphi _{1} {{\textbf{E}}}_{{\textbf{C}}}+\rho \eta {{\textbf{L}}}_{{\textbf{T}}{\textbf{C}}}+\theta _{1}\xi {{\textbf{I}}}_{{\textbf{T}}{\textbf{C}}}-(\beta +\zeta _{{\textbf{C}}}+\nu +\varphi _{3}){{\textbf{I}}}_{{\textbf{C}}},\\ \,^{c}{\textbf{D}}_{\tau }^{\omega }{{\textbf{L}}}_{{\textbf{T}}{\textbf{C}}}=\lambda \psi _{{\textbf{C}}}{{\textbf{L}}}_{{\textbf{T}}}+\epsilon \psi _{{\textbf{T}}}{{\textbf{E}}}_{{\textbf{C}}}-(\beta +\zeta _{{\textbf{C}}}+\rho +\eta ){{\textbf{L}}}_{{\textbf{T}}{\textbf{C}}},\\ \,^{c}{\textbf{D}}_{\tau }^{\omega }{{\textbf{I}}}_{{\textbf{T}}{\textbf{C}}}=\rho {{\textbf{L}}}_{{\textbf{T}}{\textbf{C}}}+\varsigma _{3}{{\textbf{I}}}_{{\textbf{T}}}+\nu {{\textbf{I}}}_{{\textbf{C}}}-(\beta +\zeta _{{\textbf{T}}{\textbf{C}}}+\xi ){{\textbf{I}}}_{{\textbf{T}}{\textbf{C}}},\\ \,^{c}{\textbf{D}}_{\tau }^{\omega }{{\textbf{R}}}=\varpi {{\textbf{L}}}_{{\textbf{T}}}+\varphi _{2}{{\textbf{E}}}_{{\textbf{C}}}+\delta {{\textbf{I}}}_{{\textbf{T}}}+\varphi _{3}{{\textbf{I}}}_{{\textbf{C}}}+(1-(\varsigma _{1}+\varsigma _{2}))\eta {{\textbf{L}}}_{{\textbf{T}}{\textbf{C}}}+(1-(\theta _{1}+\theta _{2}))\xi {{\textbf{I}}}_{{\textbf{T}}{\textbf{C}}}-\beta {\textbf{R}},\end{array}\right. } \end{aligned}$$

4 $$\begin{aligned} {\left\{ \begin{array}{ll} \,^{CF}{\textbf{D}}_{\tau }^{\omega }{\textbf{S}}=\nabla -(\psi _{{\textbf{T}}}+\psi _{{\textbf{C}}}+\beta ){\textbf{S}},\\ \,^{CF}{\textbf{D}}_{\tau }^{\omega }{{\textbf{L}}}_{{\textbf{T}}}=\psi _{{\textbf{T}}}{\textbf{S}}-(\beta +\mu +\lambda \psi _{{\textbf{C}}}+\varpi ){{\textbf{L}}}_{{\textbf{T}}},\\ \,^{CF}{\textbf{D}}_{\tau }^{\omega }{{\textbf{I}}}_{{\textbf{T}}}=\mu {{\textbf{L}}}_{{\textbf{T}}}+\varsigma _{2}\eta {{\textbf{L}}}_{{\textbf{T}}{\textbf{C}}}+\theta _{2}\xi {{\textbf{I}}}_{{\textbf{T}}{\textbf{C}}}-(\beta +\varsigma _{3}+\zeta _{{\textbf{T}}}+\delta ){{\textbf{I}}}_{{\textbf{T}}},\\ \,^{CF}{\textbf{D}}_{\tau }^{\omega }{{\textbf{E}}}_{{\textbf{C}}}=\psi _{{\textbf{C}}}{\textbf{S}}-(\beta +\epsilon \psi _{{\textbf{T}}}+\varphi _{1}+\varphi _{2}){{\textbf{E}}}_{{\textbf{C}}},~~~~~~~~~~~~~~~~~~~~~~~~~~~~~~~~~~~~~\intercal _{1}\le \tau \le \intercal _{2},\\ \,^{CF}{\textbf{D}}_{\tau }^{\omega }{{\textbf{I}}}_{{\textbf{C}}}=\varphi _{1} {{\textbf{E}}}_{{\textbf{C}}}+\rho \eta {{\textbf{L}}}_{{\textbf{T}}{\textbf{C}}}+\theta _{1}\xi {{\textbf{I}}}_{{\textbf{T}}{\textbf{C}}}-(\beta +\zeta _{{\textbf{C}}}+\nu +\varphi _{3}){{\textbf{I}}}_{{\textbf{C}}},\\ \,^{CF}{\textbf{D}}_{\tau }^{\omega }{{\textbf{L}}}_{{\textbf{T}}{\textbf{C}}}=\lambda \psi _{{\textbf{C}}}{{\textbf{L}}}_{{\textbf{T}}}+\epsilon \psi _{{\textbf{T}}}{{\textbf{E}}}_{{\textbf{C}}}-(\beta +\zeta _{{\textbf{C}}}+\rho +\eta ){{\textbf{L}}}_{{\textbf{T}}{\textbf{C}}},\\ \,^{CF}{\textbf{D}}_{\tau }^{\omega }{{\textbf{I}}}_{{\textbf{T}}{\textbf{C}}}=\rho {{\textbf{L}}}_{{\textbf{T}}{\textbf{C}}}+\varsigma _{3}{{\textbf{I}}}_{{\textbf{T}}}+\nu {{\textbf{I}}}_{{\textbf{C}}}-(\beta +\zeta _{{\textbf{T}}{\textbf{C}}}+\xi ){{\textbf{I}}}_{{\textbf{T}}{\textbf{C}}},\\ \,^{CF}{\textbf{D}}_{\tau }^{\omega }{{\textbf{R}}}=\varpi {{\textbf{L}}}_{{\textbf{T}}}+\varphi _{2}{{\textbf{E}}}_{{\textbf{C}}}+\delta {{\textbf{I}}}_{{\textbf{T}}}+\varphi _{3}{{\textbf{I}}}_{{\textbf{C}}}+(1-(\varsigma _{1}+\varsigma _{2}))\eta {{\textbf{L}}}_{{\textbf{T}}{\textbf{C}}}+(1-(\theta _{1}+\theta _{2}))\xi {{\textbf{I}}}_{{\textbf{T}}{\textbf{C}}}-\beta {\textbf{R}},\end{array}\right. } \end{aligned}$$

5 $$\begin{aligned} {\left\{ \begin{array}{ll} \,^{ABC}{\textbf{D}}_{\tau }^{\omega }{\textbf{S}}=\nabla -(\psi _{{\textbf{T}}}+\psi _{{\textbf{C}}}+\beta ){\textbf{S}},\\ \,^{ABC}{\textbf{D}}_{\tau }^{\omega }{{\textbf{L}}}_{{\textbf{T}}}=\psi _{{\textbf{T}}}{\textbf{S}}-(\beta +\mu +\lambda \psi _{{\textbf{C}}}+\varpi ){{\textbf{L}}}_{{\textbf{T}}},\\ \,^{ABC}{\textbf{D}}_{\tau }^{\omega }{{\textbf{I}}}_{{\textbf{T}}}=\mu {{\textbf{L}}}_{{\textbf{T}}}+\varsigma _{2}\eta {{\textbf{L}}}_{{\textbf{T}}{\textbf{C}}}+\theta _{2}\xi {{\textbf{I}}}_{{\textbf{T}}{\textbf{C}}}-(\beta +\varsigma _{3}+\zeta _{{\textbf{T}}}+\delta ){{\textbf{I}}}_{{\textbf{T}}},\\ \,^{ABC}{\textbf{D}}_{\tau }^{\omega }{{\textbf{E}}}_{{\textbf{C}}}=\psi _{{\textbf{C}}}{\textbf{S}}-(\beta +\epsilon \psi _{{\textbf{T}}}+\varphi _{1}+\varphi _{2}){{\textbf{E}}}_{{\textbf{C}}},~~~~~~~~~~~~~~~~~~~~~~~~~~~~~~~~~~~\intercal _{1}\le \tau \le \intercal _{2},\\ \,^{ABC}{\textbf{D}}_{\tau }^{\omega }{{\textbf{I}}}_{{\textbf{C}}}=\varphi _{1} {{\textbf{E}}}_{{\textbf{C}}}+\rho \eta {{\textbf{L}}}_{{\textbf{T}}{\textbf{C}}}+\theta _{1}\xi {{\textbf{I}}}_{{\textbf{T}}{\textbf{C}}}-(\beta +\zeta _{{\textbf{C}}}+\nu +\varphi _{3}){{\textbf{I}}}_{{\textbf{C}}},\\ \,^{ABC}{\textbf{D}}_{\tau }^{\omega }{{\textbf{L}}}_{{\textbf{T}}{\textbf{C}}}=\lambda \psi _{{\textbf{C}}}{{\textbf{L}}}_{{\textbf{T}}}+\epsilon \psi _{{\textbf{T}}}{{\textbf{E}}}_{{\textbf{C}}}-(\beta +\zeta _{{\textbf{C}}}+\rho +\eta ){{\textbf{L}}}_{{\textbf{T}}{\textbf{C}}},\\ \,^{ABC}{\textbf{D}}_{\tau }^{\omega }{{\textbf{I}}}_{{\textbf{T}}{\textbf{C}}}=\rho {{\textbf{L}}}_{{\textbf{T}}{\textbf{C}}}+\varsigma _{3}{{\textbf{I}}}_{{\textbf{T}}}+\nu {{\textbf{I}}}_{{\textbf{C}}}-(\beta +\zeta _{{\textbf{T}}{\textbf{C}}}+\xi ){{\textbf{I}}}_{{\textbf{T}}{\textbf{C}}},\\ \,^{ABC}{\textbf{D}}_{\tau }^{\omega }{{\textbf{R}}}=\varpi {{\textbf{L}}}_{{\textbf{T}}}+\varphi _{2}{{\textbf{E}}}_{{\textbf{C}}}+\delta {{\textbf{I}}}_{{\textbf{T}}}+\varphi _{3}{{\textbf{I}}}_{{\textbf{C}}}+(1-(\varsigma _{1}+\varsigma _{2}))\eta {{\textbf{L}}}_{{\textbf{T}}{\textbf{C}}}+(1-(\theta _{1}+\theta _{2}))\xi {{\textbf{I}}}_{{\textbf{T}}{\textbf{C}}}-\beta {\textbf{R}}.\end{array}\right. } \end{aligned}$$

The arrangement of this article is as follows: In “Codynamics model and preliminaries” section, explanations for fractional calculus, along with several key notions and model (2) details, are provided. Moreover, a detailed analysis of the FO co-infection system’s (3) equilibrium stability is presented in “Codynamics model and preliminaries” section. In “Stochastic configuration of codynamics of TB-COVID-19 model” section, a probabilistic form of the TB and COVID-19 models’ (28) codynamics is proposed and a detailed description of the unique global positive solution for each positive initial requirement is presented. The dynamical characteristics of the mechanism’s appropriate conditions for the presence of the distinctive stationary distribution are provided. The P.D.F enclosing a quasi-stable equilibrium of the probabilistic COVID-19 framework is presented in “Stochastic COVID-19 model without TB infection” section. Numerous numerical simulations in view of piecewise fractional derivative operators are presented in “Numerical solutions of co-dynamics model using random perturbations” section to validate the diagnostic findings we obtained in “Stochastic configuration of codynamics of TB-COVID-19 model” and “Stochastic COVID-19 model without TB infection” sections. In conclusion, we conceal our findings to conclude this study.

### Positivity and boundedness

Since we interact with living communities, each approach ought to be constructive and centred on a workable area. We utilized the subsequent hypothesis that guarantees these.

#### Theorem 1

Assume that the set ${\tilde{\Xi }}:=\Big ({{\textbf{S}}},{{\textbf{L}}}_{{\textbf{T}}},{{\textbf{I}}}_{{\textbf{T}}},{{\textbf{E}}}_{{\textbf{C}}},{{\textbf{I}}}_{{\textbf{C}}},{{\textbf{L}}}_{{\textbf{T}}{\textbf{C}}},{{\textbf{I}}}_{{\textbf{T}}{\textbf{C}}},{{\textbf{R}}}\Big )$ is a positive invariant set for the suggested FO model (3).

#### Proof

In order to demonstrate whether the solution to a set of equations (3) is positive, then (3) yields

6 $$\begin{aligned} {\left\{ \begin{array}{ll} \,_{0}^{c}{\textbf{D}}_{\tau }^{\omega }{{\textbf{S}}}\big \vert _{{{\textbf{S}}}=0}=\nabla \ge 0,\\ \,_{0}^{c}{\textbf{D}}_{\tau }^{\omega }{{{\textbf{L}}}_{{\textbf{T}}}}\big \vert _{{{\textbf{L}}}_{{\textbf{T}}}}=\psi _{{\textbf{T}}}{{\textbf{S}}}\ge 0,\\ \,_{0}^{c}{\textbf{D}}_{\tau }^{\omega }{{{\textbf{I}}}_{{\textbf{T}}}}\big \vert _{{{\textbf{I}}}_{{\textbf{T}}}=0}=\mu {{\textbf{L}}}_{{\textbf{T}}}\ge 0,\\ \,_{0}^{c}{\textbf{D}}_{\tau }^{\omega }{{{\textbf{E}}}_{{\textbf{C}}}}\big \vert _{{{\textbf{E}}}_{{\textbf{C}}}=0}=\psi _{{\textbf{C}}}{{\textbf{S}}}\ge 0,\\ \,_{0}^{c}{\textbf{D}}_{\tau }^{\omega }{{{\textbf{I}}}_{{\textbf{C}}}}\big \vert _{{{\textbf{I}}}_{{\textbf{C}}}=0}=\varphi _{1}{{\textbf{E}}}_{{\textbf{C}}}\ge 0,\\ \,_{0}^{c}{\textbf{D}}_{\tau }^{\omega }{{{\textbf{L}}}_{{\textbf{T}}{\textbf{C}}}}\big \vert _{{{\textbf{L}}}_{{\textbf{T}}{\textbf{C}}}=0}=\lambda \psi _{{\textbf{C}}}{{\textbf{L}}}_{{\textbf{T}}}+\epsilon \psi _{{\textbf{T}}}{{\textbf{E}}}_{{\textbf{C}}}\ge 0,\\ \,_{0}^{c}{\textbf{D}}_{\tau }^{\omega }{{{\textbf{I}}}_{{\textbf{T}}{\textbf{C}}}}\big \vert _{{{\textbf{I}}}_{{\textbf{T}}{\textbf{C}}}=0}=\rho {{\textbf{L}}}_{{\textbf{T}}{\textbf{C}}}+\varsigma _{3}{{\textbf{I}}}_{{\textbf{T}}}+\nu {{\textbf{I}}}_{{\textbf{C}}}\ge 0,\\ \,_{0}^{c}{\textbf{D}}_{\tau }^{\omega }{{\textbf{R}}}\big \vert _{{\textbf{R}}=0}=\varpi {{\textbf{L}}}_{{\textbf{T}}}+\varphi _{2}{{\textbf{E}}}_{{\textbf{C}}}+\delta {{\textbf{I}}}_{{\textbf{T}}}+\varphi _{3}{{\textbf{I}}}_{{\textbf{C}}}+(1-(\varsigma _{1}+\varsigma _{2}))\eta {L_{{\textbf{T}}{\textbf{C}}}}+(1-(\theta _{1}+\theta _{2}))\xi {{{\textbf{I}}}_{{\textbf{T}}{\textbf{C}}}}\ge 0. \end{array}\right. } \end{aligned}$$

Therefore, the outcomes related to the FO model (3) are positive. Finally, the variation in the entire community is described by

$$\begin{aligned} \,_{0}^{c}{\textbf{D}}_{\tau }^{\omega }{\tilde{\Xi }}{} & {} \le \nabla +\zeta _{{\textbf{T}}}{{\textbf{I}}}_{{\textbf{T}}}-\zeta _{{\textbf{C}}}({{\textbf{I}}}_{{\textbf{C}}}+{{\textbf{L}}}_{{\textbf{T}}{\textbf{C}}})-\zeta _{{\textbf{T}}{\textbf{C}}}{{\textbf{I}}}_{{\textbf{T}}{\textbf{C}}}-\beta {\textbf{N}}\nonumber \\{} & {} \le \nabla -\beta {{\textbf{N}}}. \end{aligned}$$

Addressing the variant previously mentioned, we get

$$\begin{aligned} {\tilde{\Xi }}(\tau )\le \bigg ({\tilde{\Xi }}(0)-\frac{\nabla }{\beta }\bigg )E_{\omega }\bigg (-\beta \tau ^{\omega }\bigg )+\frac{\nabla }{\beta }. \end{aligned}$$

Consequently, we derive the GML function’s asymptotic operation^43^ as

$$\begin{aligned} {\tilde{\Xi }}(\tau )\le \frac{\nabla }{\beta }. \end{aligned}$$

Adopting the same procedure for other systems of equations in the model (3), which indicates that the closed set ${\tilde{\Xi }}$ is a positive invariant domain for the FO system (3).$\square$

- Assuming that every requirement is non-negative throughout time $\tau$, we exhibit that the outcomes remain non-negative and bounded in the proposed region, $\Pi$. We’ll look at the co-infection model (3) ${\tilde{\Xi }}:=\Big ({{\textbf{S}}},{{\textbf{L}}}_{{\textbf{T}}},{{\textbf{I}}}_{{\textbf{T}}},{{\textbf{E}}}_{{\textbf{C}}},{{\textbf{I}}}_{{\textbf{C}}},{{\textbf{L}}}_{{\textbf{T}}{\textbf{C}}},{{\textbf{I}}}_{{\textbf{T}}{\textbf{C}}},{{\textbf{R}}}\Big )$ spreads in the domain, which is described as $\Pi :=\Big \{{\tilde{\Xi }}\in \Re _{+}^{8}:0\le {\textbf{N}}\le \frac{\nabla }{\beta }\Big \}.$
- According to the afflicted categories in co-infection model (3), disease-free equilibrium (DFE) and endemic equilibrium (EE) are the biologically significant steady states of FO model (3). We establish the fractional derivative to get the immune-to-infection steady state as $\,_{0}^{c}{\textbf{D}}_{\tau }^{\omega }{{\textbf{S}}},~\,_{0}^{c}{\textbf{D}}_{\tau }^{\omega }{{\textbf{L}}}_{{\textbf{T}}},~\,_{0}^{c}{\textbf{D}}_{\tau }^{\omega }{{\textbf{I}}}_{{\textbf{T}}},\,_{0}^{c}{\textbf{D}}_{\tau }^{\omega }{{\textbf{E}}}_{{\textbf{C}}},~\,_{0}^{c}{\textbf{D}}_{\tau }^{\omega }{{\textbf{I}}}_{{\textbf{C}}}, \,_{0}^{c}{\textbf{D}}_{\tau }^{\omega }{{\textbf{L}}}_{{\textbf{T}}{\textbf{C}}},~\,_{0}^{c}{\textbf{D}}_{\tau }^{\omega }{{\textbf{I}}}_{{\textbf{T}}{\textbf{C}}},~\,_{0}^{c}{\textbf{D}}_{\tau }^{\omega }{{\textbf{R}}},$ to zero of the FO model (3) have no infection, and get
  $$\begin{aligned} {\mathcal {E}}_{0}=\Big (\frac{\nabla }{\beta },0,0,0,0,0,0,0\Big ). \end{aligned}$$
- The dominating eigenvalue of the matrix ${\textbf{F}}{\textbf{G}}^{-1}$ correlates with the basic reproductive quantity ${\mathbb {R}}_{0}^{CT}$ of structure (3), in accordance with the next generation matrix approach^44^. Thus, we find
  $$\begin{aligned}{\mathcal {F}}= \begin{pmatrix} \psi _{{\textbf{T}}}{{\textbf{S}}}\\ 0\\ \psi _{{\textbf{C}}}{\textbf{S}}\\ 0\\ 0\\ 0 \end{pmatrix},~~~\Phi =\begin{pmatrix} (\beta +\mu +\lambda \psi _{{\textbf{C}}}+\varpi ){{\textbf{L}}}_{{\textbf{T}}}\\ -\theta _{2}\xi {{\textbf{I}}}_{{\textbf{T}}{\textbf{C}}}-\varsigma _{2}\eta {{\textbf{L}}}_{{\textbf{T}}{\textbf{C}}}-\mu {{\textbf{L}}}_{{\textbf{T}}}+(\beta +\varsigma _{3}+\zeta _{{\textbf{T}}}+\delta ){{\textbf{I}}}_{{\textbf{T}}}\\ (\beta +\epsilon \psi _{{\textbf{T}}}+\varphi _{1}+\varphi _{2}){{\textbf{E}}}_{{\textbf{C}}}\\ -\varphi _{1}{{\textbf{E}}}_{{\textbf{C}}}-\varsigma _{1}\eta {{\textbf{L}}}_{{\textbf{T}}{\textbf{C}}}-\theta _{1}\xi {{\textbf{I}}}_{{\textbf{T}}{\textbf{C}}}+(\beta +\nu +\zeta _{{\textbf{C}}}+\varphi _{3}){{\textbf{I}}}_{{\textbf{C}}}\\ -\lambda \psi _{{\textbf{C}}}{{\textbf{L}}}_{{\textbf{T}}}-\epsilon \psi _{{\textbf{T}}}{{\textbf{E}}}_{{\textbf{C}}}+(\beta +\zeta _{{\textbf{C}}}+\rho +\eta ){{\textbf{L}}}_{{\textbf{T}}{\textbf{C}}}\\ -\rho {{\textbf{L}}}_{{\textbf{T}}{\textbf{C}}}-\varsigma _{3}{{\textbf{I}}}_{{\textbf{T}}}-\nu {{\textbf{I}}}_{{\textbf{C}}}+(\beta +\zeta _{{\textbf{T}}{\textbf{C}}}+\xi ) \end{pmatrix}. \end{aligned}$$

The next generation matrix at DEF can then be obtained by using the Jacobian of ${\textbf{F}}$ and ${\textbf{G}}$ examined at ${\mathcal {E}}_{0}$ as

$$\begin{aligned} {\textbf{F}}{\textbf{G}}^{-1}=\begin{pmatrix} \frac{\mu {\mathcal {K}}_{1}}{(\beta +\mu +\varpi ){\mathcal {K}}_{7}}&{}\frac{{\mathcal {K}}_{1}}{{\mathcal {K}}_{7}}&{}\frac{\varphi _{1}{\mathcal {K}}_{3}}{(\beta +\omega +\varphi _{2}){\mathcal {K}}_{7}}&{}\frac{{\mathcal {K}}_{3}}{{\mathcal {K}}_{7}}&{}\frac{{\mathcal {K}}_{5}}{(\beta +\zeta _{{\textbf{C}}}+\rho +\eta ){\mathcal {K}}_{7}}&{}\frac{-\alpha _{1}(\beta +\nu +\zeta _{{\textbf{C}}}+\varphi _{3})(\beta +\varsigma _{3}+\zeta _{{\textbf{T}}}\delta +\theta _{2}\xi )}{{\mathcal {K}}_{7}}\\ 0&{}0&{}0&{}0&{}0&{}0\\ \frac{\mu {\mathcal {K}}_{2}}{(\beta +\mu +\varpi ){\mathcal {K}}_{7}}&{}\frac{{\mathcal {K}}_{2}}{{\mathcal {K}}_{7}}&{}\frac{\varphi _{1}{\mathcal {K}}_{4}}{(\beta +\omega +\varphi _{2}){\mathcal {K}}_{7}}&{}\frac{-\alpha _{2}(\beta +\varsigma _{3}+\zeta _{{\textbf{T}}}+\delta )(\beta +\xi +\zeta _{{\textbf{T}}{\textbf{C}}}+\nu )-\theta _{2}\varsigma _{3}\xi }{{\mathcal {K}}_{7}}&{}\frac{{\mathcal {K}}_{6}}{(\beta +\varphi _{1}+\varphi _{2}){\mathcal {K}}_{7}}&{}\frac{-\alpha _{2}(\beta +\varsigma _{3}+\zeta _{{\textbf{T}}}+\delta )(\beta +\zeta _{{\textbf{C}}}+\rho +\eta )}{{\mathcal {K}}_{7}}\\ 0&{}0&{}0&{}0&{}0&{}0\\ 0&{}0&{}0&{}0&{}0&{}0\\ 0&{}0&{}0&{}0&{}0&{}0 \end{pmatrix}, \end{aligned}$$

where

$$\begin{aligned} {\mathcal {K}}_{\kappa }= {\left\{ \begin{array}{ll} -\alpha _{1}\big ((\beta +\nu +\zeta _{{\textbf{C}}}+\varphi _{3})(\beta +\xi +\zeta _{{\textbf{T}}{\textbf{C}}})+(\beta +\nu +\zeta _{{\textbf{C}}}+\varphi _{3})\varsigma _{3}-\theta _{1}\nu \xi \big ),~~~~\kappa =1,\\ -\alpha _{2}\varsigma _{3}(\theta _{1}\xi +\beta +\nu +\zeta _{{\textbf{C}}}+\varphi _{3}),~~~~\kappa =2,\\ -\alpha _{1}\nu (\theta _{2}\xi +\beta +\varsigma _{3}+\zeta _{{\textbf{T}}}+\delta ),~~~~\kappa =3,\\ -\alpha _{2}(\beta +\varsigma _{3}+\zeta _{{\textbf{T}}}+\delta )\big ((\beta +\nu +\varphi _{3}+\zeta _{{\textbf{C}}})(\beta +\xi +\zeta _{{\textbf{T}}{\textbf{C}}})+\varphi _{1}(\beta +\xi +\zeta _{{\textbf{T}}{\textbf{C}}})+\nu \varphi _{1}-\theta _{1}\nu \xi \big )\\ \qquad -\theta _{2}\varsigma _{3}\xi (\varphi _{1}+\beta +\nu +\zeta _{{\textbf{C}}}+\varphi _{3}),~~~~\kappa =4,\\ \nu \rho \xi (\theta _{2}\varsigma _{1}-\theta _{1}\varsigma _{2})+(\beta +\nu +\zeta _{{\textbf{C}}}+\varphi _{3})\big (-\alpha _{1}\rho (\nu \xi +\beta +\varsigma _{3}+\zeta _{{\textbf{T}}}+\delta )+\eta \varsigma _{2}(\beta +\xi +\varsigma _{3}+\zeta _{{\textbf{T}}{\textbf{C}}})\big )\\ \qquad +\varsigma _{1}\nu \eta (\beta +\varsigma _{3}+\zeta _{{\textbf{T}}}+\delta ),~~~~\kappa =5,\\ -\alpha _{2}\big ((\beta +\varsigma _{3}+\zeta _{{\textbf{T}}}+\delta )(\beta +\nu +\zeta _{C{1}}+\varphi _{3})(\beta +\xi +\zeta _{{\textbf{T}}{\textbf{C}}}+\rho )+\theta _{1}\xi (\rho -\nu )(\beta +\varsigma _{3}+\zeta _{{\textbf{T}}}+\delta )\\ \qquad +(\beta +\varsigma _{3}+\zeta _{{\textbf{T}}}+\delta )\eta \varsigma _{1}(\beta +\xi +\zeta _{{\textbf{T}}{\textbf{C}}}+\nu )+\varsigma _{2}\eta \varsigma _{3}(\beta +\nu +\zeta _{{\textbf{C}}}+\varphi _{3}+\theta _{1}\xi )\\ \qquad -\theta _{2}\varsigma _{3}\xi (\varsigma _{1}\eta +\beta +\nu +\zeta _{{\textbf{C}}}+\varphi _{3})\big ),~~~\kappa =6,\\ \theta _{1}\nu \xi (\beta +\varsigma _{3}+\zeta _{{\textbf{T}}}+\delta )+(\beta +\zeta _{{\textbf{C}}}+\rho +\eta )\big (\theta _{2}\varsigma _{3}\xi -(\beta +\varsigma _{3}+\zeta _{{\textbf{T}}}+\delta )(\beta =\xi +\zeta _{{\textbf{T}}{\textbf{C}}})\big ),~~~\kappa =7. \end{array}\right. } \end{aligned}$$

The fundamental reproducing quantity of the pairing system is shown by the highest spectral radius of the subsequent generation’s matrix. It is evident that the matrix ${\textbf{F}}{\textbf{G}}^{-1}$ has four eigenvalues that are equivalent to zero. The truncated matrix yields the additional eigenvalues as

$$\begin{pmatrix}\frac{\mu {\mathcal {K}}_{1}}{(\beta +\mu +\varpi ){\mathcal {K}}}&\frac{\varphi _{1}{\mathcal {K}}_{3}}{(\beta +\varphi _{1}+\varphi _{2}){\mathcal {K}}} \frac{\mu {\mathcal {K}}_{2}}{(\beta +\mu +\varpi ){\mathcal {K}}}&\frac{{\mathcal {K}}_{4}}{(\beta +\varphi _{1}+\varphi _{2}){\mathcal {K}}}\end{pmatrix}.$$

Consequently, by calculating the eigenvalues of ${\textbf{F}}{\textbf{G}}^{-1}$, it is possible to simply determine that

$$\begin{aligned} \tilde{\delta _{1}}{} & {} =\frac{(\beta +\mu +\varpi ){\textbf{Q}}_{4}+\mu (\beta +\varphi _{1}+\varphi _{2}){\textbf{Q}}_{1}-\nabla _{1}^{2}}{2(\beta +\mu +\varpi )(\beta +\varphi _{1}+\varphi _{2})\big ( \theta _{1}\nu \xi (\beta +\varsigma _{3}+\zeta _{{\textbf{T}}}+\delta )+(\beta +\zeta _{{\textbf{C}}}+\rho +\eta )\big (\theta _{2}\varsigma _{3}\xi -(\beta +\varsigma _{3}+\zeta _{{\textbf{T}}}+\delta )(\beta +\xi +\zeta 
_{{\textbf{T}}{\textbf{C}}})\big )\big )},\nonumber \\ \tilde{\delta _{2}}{} & {} =\frac{(\beta +\mu +\varpi ){\textbf{Q}}_{4}+\mu (\beta +\varphi _{1}+\varphi _{2}){\textbf{Q}}_{1}+\nabla _{1}^{2}}{2(\beta +\mu +\varpi )(\beta +\varphi _{1}+\varphi _{2})\big ( \theta _{1}\nu \xi (\beta +\varsigma _{3}+\zeta _{{\textbf{T}}}+\delta )+(\beta +\zeta _{{\textbf{C}}}+\rho +\eta )\big (\theta _{2}\varsigma _{3}\xi -(\beta +\varsigma _{3}+\zeta _{{\textbf{T}}}+\delta )(\beta +\xi +\zeta _{{\textbf{T}}{\textbf{C}}})\big )\big )}, \end{aligned}$$

where

$$\begin{aligned} \nabla _{1}=\sqrt{\mu ^{2}{\mathcal {K}}_{1}^{2}(\beta +\varphi _{1}+\varphi _{2})^{2}-2\mu (\beta +\mu +\varpi )(\beta +\varphi _{1}+\varphi _{2}){\textbf{Q}}_{1}{\textbf{Q}}_{2}+4\varphi _{1}\mu (\beta +\mu +\varpi )(\beta +\varphi _{1}+\varphi _{2}){\mathcal {K}}_{2}^{2}+(\beta +\mu +\varpi )^{2}{\mathcal {K}}_{4}^{2}}. \end{aligned}$$

Therefore, the co-dynamics structure’s (3) fundamental reproductive quantity ${\mathbb {R}}_{0}$ is provided by ${\mathbb {R}}_{0}^{CT}=\max \{{\mathbb {R}}_{0}^{C},{\mathbb {R}}_{0}^{T}\}.$

Here, we shall then demonstrate how transmission persists in the FO mechanism. It explains how widespread the virus is within the framework. From the viewpoint of biology, the virus continues in the bloodstream if the infectious proportion is elevated for a sufficiently long time $\tau$.

However, the linearization technique is used to examine the local stabilization of the codynamics algorithm’s DFE state. At the DFE state ${\mathcal {E}}_{0},$ the Jacobean matrix of system (3) is displayed as

7 $$\begin{aligned} {\mathcal {J}}_{{\mathcal {E}}_{0}}=\begin{pmatrix} -\beta &{}0&{}-\alpha _{1}&{}-\alpha _{2}&{}-\alpha _{2}&{}-\alpha _{2}&{}-(\alpha _{1}+\alpha _{2})&{}0\\ 0&{}-(\beta +\mu +\varpi )&{}\alpha _{1}&{}0&{}0&{}0&{}\alpha _{1}&{}0\\ 0&{}\mu &{}-(\beta +\varsigma _{3}+\zeta _{{\textbf{T}}}+\delta )&{}0&{}0&{}\varsigma _{2}\eta &{}\theta _{2}\xi &{}0\\ 0&{}0&{}0&{}\alpha _{2}-\beta -\varphi _{1}-\varphi _{2}&{}\alpha _{2}&{}\alpha _{2}&{}\alpha _{2}&{}0\\ 0&{}0&{}0&{}\varphi _{1}&{}-\beta -\nu -\zeta _{{\textbf{C}}}-\varphi _{3}&{}\varsigma _{1}\eta &{}\theta _{1}\xi &{}0\\ 0&{}0&{}0&{}0&{}0&{}-(\beta +\zeta _{{\textbf{C}}}+\rho +\eta )&{}0&{}0\\ 0&{}0&{}\varsigma _{3}&{}0&{}\nu &{}\rho &{}-(\beta +\xi +\zeta _{{\textbf{T}}{\textbf{C}}})&{}0\\ 0&{}\varpi &{}\delta &{}\varphi _{2}&{}\varphi _{3}&{}(1-(\varsigma _{1}+\varsigma _{2}))\eta &{}(1-(\theta _{1}+\theta _{2}))\eta &{}-\beta . \end{pmatrix} \end{aligned}$$

The analysis of ${\mathcal {E}}_{0}$’s localized temporal equilibrium relies upon the eigenvalues’ interpretation. Here, $\tilde{\delta _{1,2}}=-\beta$ and $\tilde{\delta _{3}}=-(\beta +\rho +\eta +\zeta _{{\textbf{C}}})$ are obtained by broadening the following polynomial $\vert {\mathcal {J}}_{{\mathcal {E}}_{0}}-\delta {\mathcal {I}}\vert =0$. Moreover, we get the additional $\delta$’s based on the simplified matrix’s $\vert {\mathcal {J}}_{{\mathcal {E}}_{0}}-\delta {\mathcal {I}}\vert =0$ described as

$$\begin{aligned}{} & {} {\mathcal {J}}-\delta {\mathcal {I}}_{5}\nonumber \\ {}{} & {} =\begin{pmatrix} \mu &{}-(\beta +\varsigma _{3}+\delta +\zeta _{{\textbf{T}}}+{\tilde{\delta }})&{}0&{}0&{}\theta _{2}\xi \\ 0&{}\varsigma _{3}&{}0&{}\nu &{}-({\tilde{\delta }}+\beta +\xi +\zeta _{{\textbf{C}}})\\ 0&{}0&{}\varphi _{1}&{}-({\tilde{\delta }}+\beta +\nu +\varphi _{3}+\zeta _{{\textbf{C}}})&{}\theta _{1}\xi \\ 0&{}0&{}0&{}\alpha _{2}\varphi _{1}+({\tilde{\delta }}+\beta +\nu +\varphi _{3}+\zeta _{{\textbf{C}}})(\alpha _{2}-{\tilde{\delta }}-\beta -\xi -\varphi _{1})/\varphi _{1}&{}\Im _{1}\\ 0&{}0&{}0&{}0&{}\Im _{2} \end{pmatrix}, \end{aligned}$$

where $\Im _{1}=\alpha _{2}\varphi _{1}+({\tilde{\delta }}+\beta +\nu +\varphi _{3}+\zeta _{{\textbf{C}}})(\alpha _{2}-{\tilde{\delta }}-\beta -\xi -\varphi _{1})/\varphi _{1},\Im _{2}=\alpha _{1}(\varsigma _{3}+({\tilde{\delta }}+\beta +\xi +\zeta _{{\textbf{C}}}))/\varsigma _{3}+\big (\beta +\varsigma _{3}+\zeta _{{\textbf{T}}}+\delta +{\tilde{\delta }}/\mu \big )\big (\theta _{2}\xi \varsigma _{3}-(\beta +\varsigma _{3}+\zeta _{{\textbf{T}}}+\delta +{\tilde{\delta }})(\beta +\xi +\zeta _{{\textbf{T}}{\textbf{C}}}+{\tilde{\delta }})/\varsigma _{3})\big ).$ After simple computations, the characteristic polynomial of the above matrix is presented as

8 $$\begin{aligned} {\textbf{U}}({\tilde{\delta }}){} & {} =-\mu \varsigma _{3}\varphi _{1} \frac{\alpha _{2}\varphi _{1}+({\tilde{\delta }}+\beta +\nu +\varphi _{3}+\zeta _{{\textbf{C}}})(\alpha _{2}-{\tilde{\delta }}-\beta -\xi -\varphi _{1})}{\varphi _{1}}\Bigg \{\frac{\alpha _{1}(\varsigma _{3}+({\tilde{\delta }}+\beta +\xi +\zeta _{{\textbf{C}}}))}{\varsigma _{3}}\\{} & {} \quad +\frac{(\beta +\varsigma _{3}+\zeta _{{\textbf{T}}}+\delta +{\tilde{\delta }}}{\mu }\frac{\theta _{2}\xi \varsigma _{3}-(\beta +\varsigma _{3}+\zeta _{{\textbf{T}}}+\delta +{\tilde{\delta }})(\beta +\xi +\zeta _{{\textbf{T}}{\textbf{C}}}+{\tilde{\delta }})}{\varsigma _{3}}\Bigg \}. \end{aligned}$$

In other words, the outcomes to the ${\textbf{U}}({\tilde{\delta }})$ are the eigenvalues:

9 $$\begin{aligned} {\textbf{U}}({\tilde{\delta }})={\tilde{\delta }}^{5}+{\textbf{d}}_{1}{\tilde{\delta }}^{4}+{\textbf{d}}_{2}{\tilde{\delta }}^{3}+{\textbf{d}}_{3}{\tilde{\delta }}^{2}+{\textbf{d}}_{4}{\tilde{\delta }}+{\textbf{d}}_{5}=0, \end{aligned}$$

where

10 $$\begin{aligned} {\textbf{d}}_{1}{} & {} =\alpha _{2}-\beta -\varphi _{1}-\varphi _{2},\nonumber \\ {\textbf{d}}_{2}{} & {} =\alpha _{2}\varphi _{1}+(\beta +\nu +\zeta _{{\textbf{C}}}+\varphi _{3})(\alpha _{2}-\beta -\varphi _{1}-\varphi _{2})-(\alpha _{2}-2\beta -\varphi _{1}-\varphi _{2}-\nu -\zeta _{{\textbf{C}}}-\varphi _{3})\nonumber \\{} & {} \quad \times (\beta +\varsigma _{3}+\zeta _{{\textbf{T}}}+\delta +\xi +\zeta _{{\textbf{T}}{\textbf{C}}}-\mu -\varpi )+\big (\mu \alpha _{1}+\theta _{2}\xi \varsigma _{3}-(\beta +\varsigma _{3}+\zeta _{{\textbf{T}}}+\delta )(\beta +\xi +\zeta _{{\textbf{T}}{\textbf{C}}})\nonumber \\{} & {} \quad -(\beta +\varsigma _{3}+\zeta _{{\textbf{T}}}+\delta )(\beta +\mu +\varpi )-(\beta +\mu +\varpi )(\beta +\xi +\zeta _{{\textbf{T}}{\textbf{C}}})\big ),\nonumber \\ {\textbf{d}}_{3}{} & {} =\mu \alpha _{1}(\varsigma _{3}+\beta +\xi +\zeta _{{\textbf{T}}{\textbf{C}}})+(\beta +\mu +\varpi )\big (\theta _{2}\xi \varsigma _{3}-(\beta +\varsigma _{3}+\zeta _{{\textbf{T}}}+\delta )(\beta +\xi +\zeta _{{\textbf{T}}{\textbf{C}}})\big )\nonumber \\{} & {} \quad -\big (\alpha _{2}\varphi _{1}+(\beta +\nu +\varphi _{3}+\zeta _{{\textbf{C}}})(\alpha _{2}-\beta -\varphi _{1}-\varphi _{2})\big )(\beta +\varsigma _{3}+\zeta _{{\textbf{T}}}+\delta +\varphi _{1}+\varphi _{2}-\xi -\zeta _{{\textbf{T}}{\textbf{C}}})\nonumber \\ {}{} & {} \quad -(\alpha _{2}-2\beta -\varphi _{1}-\varphi _{2}-\nu -\zeta _{{\textbf{C}}}-\varphi _{3})\big (\mu \alpha _{1}+\theta _{2}\xi \varsigma _{3}-(\beta +\varsigma _{3}+\zeta _{{\textbf{T}}}+\delta )(\beta +\xi +\zeta _{{\textbf{T}}{\textbf{C}}})\nonumber \\ {}{} & {} \quad -(\beta +\mu +\varpi )(2\beta +\varsigma _{3}+\zeta _{{\textbf{T}}}+\delta +\xi +\zeta _{{\textbf{T}}{\textbf{C}}})\big ),\nonumber \\{\textbf{d}}_{4}{} & {} = -\big (\alpha _{2}\varphi _{1}+(\beta +\nu +\zeta _{{\textbf{C}}}+\varphi _{3})(\alpha _{2}-\beta -\varphi _{1}-\varphi _{2})\big )\big (\mu \alpha _{1}+\theta _{2}\xi \varsigma _{3}-(\beta +\mu +\varpi )(2\beta +\varsigma _{3}+\zeta _{{\textbf{T}}}+\delta +\xi +\zeta _{{\textbf{T}}{\textbf{C}}})\big )\nonumber \\ {}{} & {} \quad -(\alpha _{2}-2\beta -\varphi _{1}-\varphi _{2}-\nu -\zeta _{{\textbf{C}}}-\varphi _{3})\big (\mu \alpha _{1}(\varsigma _{3}+\beta +\xi +\zeta _{{\textbf{T}}{\textbf{C}}})+(\beta +\mu +\varpi )\nonumber \\ {}{} & {} \quad \times (\theta _{2}\xi \varsigma _{3}-(\beta \varsigma _{3}+\zeta _{{\textbf{T}}}+\delta )(\beta +\xi +\zeta _{{\textbf{T}}{\textbf{C}}}))\big ),\nonumber \\{\textbf{d}}_{5}{} & {} =\big (\alpha _{2}\varphi _{1}+(\beta +\nu +\varphi _{3}+\zeta _{{\textbf{C}}})(\alpha _{2}-\beta -\varphi _{1}-\varphi _{2})\big )\big (\mu \alpha _{1}(\varsigma _{3}+\beta +\xi +\zeta _{{\textbf{T}}{\textbf{C}}})\nonumber \\ {}{} & {} \quad +(\beta +\mu +\varpi )(\theta _{2}\xi \varsigma _{3}-(\beta +\varsigma _{3}+\zeta _{{\textbf{T}}}+\delta )(\beta +\xi +\zeta _{{\textbf{T}}{\textbf{C}}}))\big ). \end{aligned}$$

Therefore, if the subsequent requirements apply, the roots of expression (10) exhibit negative real portions according to the Routh–Hurwitz stability specifications as

11 $$\begin{aligned} {\left\{ \begin{array}{ll} {\textbf{d}}_{\jmath }>0,~~\forall ~\jmath =1,...,5,~{\textbf{d}}_{1}{\textbf{d}}_{2}{\textbf{d}}_{3}>{\textbf{d}}_{3}^{2}+{\textbf{d}}_{1}^{2}{\textbf{d}}_{4},\\ ({\textbf{d}}_{1}{\textbf{d}}_{4}-{\textbf{d}}_{5})({\textbf{d}}_{1}{\textbf{d}}_{2}{\textbf{d}}_{3}-{\textbf{d}}_{3}^{2}-{\textbf{d}}_{1}^{2}{\textbf{d}}_{4})>{\textbf{d}}_{5}({\textbf{d}}_{1}{\textbf{d}}_{2}-{\textbf{d}}_{3})^{2}+{\textbf{d}}_{1}{\textbf{d}}_{5}^{2}. \end{array}\right. } \end{aligned}$$

**Figure 4:**
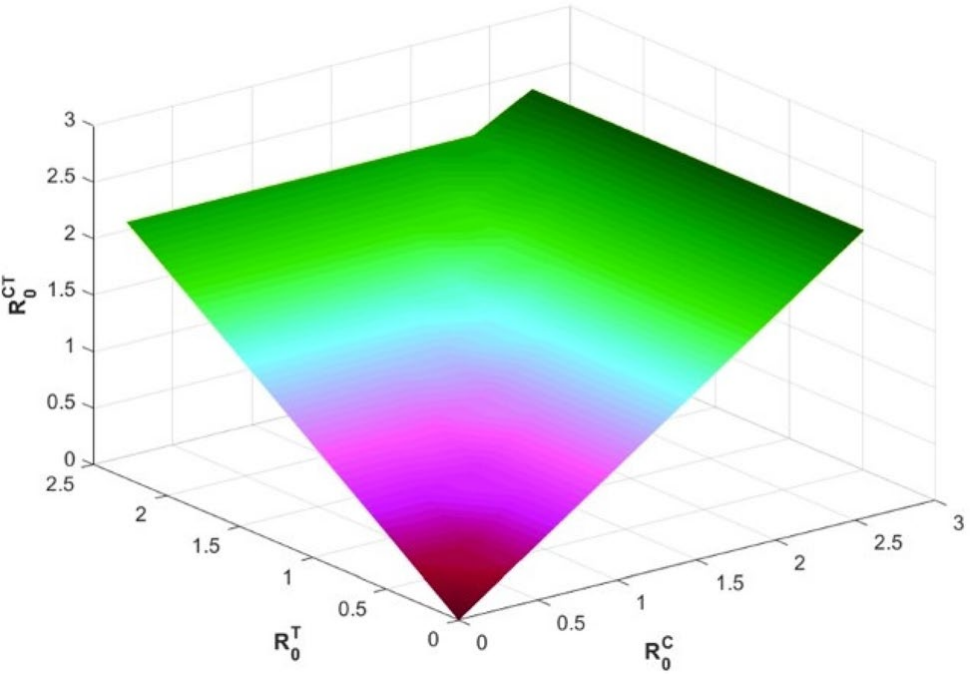
Evolution of the basic reproduction number ${\mathbb {R}}_{0}^{CT}$ with the aid of ${\mathbb {R}}_{0}^{C}$ and ${\mathbb {R}}_{0}^{T}$.

Figure 4 is illustrated by depicting in 3D evolution of the threshold parameter ${\mathbb {R}}_{0}^{CT}$ of model (3) as a function of ${\mathbb {R}}_{0}^{C}$ and ${\mathbb {R}}_{0}^{T}.$

The forthcoming result is established thanks to Theorem 2 in^44^.

#### Theorem 2

The DFE point of the FO codynamics model (3) is locally asymptotically stable if the prerequisite specified in formula (12) is satisfied.

### Existence and uniqueness of solutions

This section shows that there is only one solution for the system (3). Now, we demonstrate that the framework’s solution is distinctive. Initially, we construct framework (3) in the form of:

12 $$\begin{aligned} {\left\{ \begin{array}{ll} \,^{c}{\textbf{D}}_{\tau }^{\omega }{\textbf{S}}={\mathcal {Q}}_{1}\big (\tau ,{\textbf{S}}(\tau )\big ),\\ \,^{c}{\textbf{D}}_{\tau }^{\omega }{{\textbf{L}}}_{{\textbf{T}}}={\mathcal {Q}}_{2}\big (\tau ,{{\textbf{L}}}_{{\textbf{T}}}(\tau )\big ),\\ \,^{c}{\textbf{D}}_{\tau }^{\omega }{{\textbf{I}}}_{{\textbf{T}}}={\mathcal {Q}}_{3}\big (\tau ,{{\textbf{I}}}_{{\textbf{T}}}(\tau )\big ),\\ \,^{c}{\textbf{D}}_{\tau }^{\omega }{{\textbf{E}}}_{{\textbf{C}}}={\mathcal {Q}}_{4}\big (\tau ,{{\textbf{E}}}_{{\textbf{C}}}(\tau )\big ),\\ \,^{c}{\textbf{D}}_{\tau }^{\omega }{{\textbf{I}}}_{{\textbf{C}}}={\mathcal {Q}}_{5}\big (\tau ,{{\textbf{I}}}_{{\textbf{C}}}(\tau )\big ),\\ \,^{c}{\textbf{D}}_{\tau }^{\omega }{{\textbf{L}}}_{{\textbf{T}}{\textbf{C}}}={\mathcal {Q}}_{6}\big (\tau ,{{\textbf{L}}}_{{\textbf{T}}{\textbf{C}}}(\tau )\big ),\\ \,^{c}{\textbf{D}}_{\tau }^{\omega }{{\textbf{I}}}_{{\textbf{T}}{\textbf{C}}}={\mathcal {Q}}_{7}\big (\tau ,{{\textbf{I}}}_{{\textbf{T}}{\textbf{C}}}(\tau )\big ),\\ \,^{c}{\textbf{D}}_{\tau }^{\omega }{{\textbf{R}}}={\mathcal {Q}}_{8}\big (\tau ,{{\textbf{R}}}(\tau )\big ),\\ \end{array}\right. } \end{aligned}$$

where

13 $$\begin{aligned} {\left\{ \begin{array}{ll} {\mathcal {Q}}_{1}\big (\tau ,{\textbf{S}}(\tau )\big )=\nabla -(\psi _{{\textbf{T}}}+\psi _{{\textbf{C}}}+\beta ){\textbf{S}},\\ {\mathcal {Q}}_{2}\big (\tau ,{{\textbf{L}}}_{{\textbf{T}}}(\tau )\big )=\psi _{{\textbf{T}}}{\textbf{S}}-(\beta +\mu +\lambda \psi _{{\textbf{C}}}+\varpi ){{\textbf{L}}}_{{\textbf{T}}},\\ {\mathcal {Q}}_{3}\big (\tau ,{{\textbf{I}}}_{{\textbf{T}}}(\tau )\big )=\mu {{\textbf{L}}}_{{\textbf{T}}}+\varsigma _{2}\eta {{\textbf{L}}}_{{\textbf{T}}{\textbf{C}}}+\theta _{2}\xi {{\textbf{I}}}_{{\textbf{T}}{\textbf{C}}}-(\beta +\varsigma _{3}+\zeta _{{\textbf{T}}}+\delta ){{\textbf{I}}}_{{\textbf{T}}},\\ {\mathcal {Q}}_{4}\big (\tau ,{{\textbf{E}}}_{{\textbf{C}}}(\tau )\big )=\psi _{{\textbf{C}}}{\textbf{S}}-(\beta +\epsilon \psi _{{\textbf{T}}}+\varphi _{1}+\varphi _{2}){{\textbf{E}}}_{{\textbf{C}}},\\ {\mathcal {Q}}_{5}\big (\tau ,{{\textbf{I}}}_{{\textbf{C}}}(\tau )\big )=\varphi _{1} {{\textbf{E}}}_{{\textbf{C}}}+\rho \eta {{\textbf{L}}}_{{\textbf{T}}{\textbf{C}}}+\theta _{1}\xi {{\textbf{I}}}_{{\textbf{T}}{\textbf{C}}}-(\beta +\zeta _{{\textbf{C}}}+\nu +\varphi _{3}){{\textbf{I}}}_{{\textbf{C}}},\\ {\mathcal {Q}}_{6}\big (\tau ,{{\textbf{L}}}_{{\textbf{T}}{\textbf{C}}}(\tau )\big )=\lambda \psi _{{\textbf{C}}}{{\textbf{L}}}_{{\textbf{T}}}+\epsilon \psi _{{\textbf{T}}}{{\textbf{E}}}_{{\textbf{C}}}-(\beta +\zeta _{{\textbf{C}}}+\rho +\eta ){{\textbf{L}}}_{{\textbf{T}}{\textbf{C}}},\\ {\mathcal {Q}}_{7}\big (\tau ,{{\textbf{I}}}_{{\textbf{T}}{\textbf{C}}}(\tau )\big )=\rho {{\textbf{L}}}_{{\textbf{T}}{\textbf{C}}}+\varsigma _{3}{{\textbf{I}}}_{{\textbf{T}}}+\nu {{\textbf{I}}}_{{\textbf{C}}}-(\beta +\zeta _{{\textbf{T}}{\textbf{C}}}+\xi ){{\textbf{I}}}_{{\textbf{T}}{\textbf{C}}},\\ {\mathcal {Q}}_{8}\big (\tau ,{{\textbf{R}}}(\tau )\big )=\varpi {{\textbf{L}}}_{{\textbf{T}}}+\varphi _{2}{{\textbf{E}}}_{{\textbf{C}}}+\delta {{\textbf{I}}}_{{\textbf{T}}}+\varphi _{3}{{\textbf{I}}}_{{\textbf{C}}}+(1-(\varsigma _{1}+\varsigma _{2}))\eta {{\textbf{L}}}_{{\textbf{T}}{\textbf{C}}}+(1-(\theta _{1}+\theta _{2}))\xi {{\textbf{I}}}_{{\textbf{T}}{\textbf{C}}}-\beta {\textbf{R}}.\end{array}\right. } \end{aligned}$$

Integral transform applied to both sides of equations (14) yields

14 $$\begin{aligned} {\left\{ \begin{array}{ll} {\textbf{S}}(\tau )-{\textbf{S}}(0)=\frac{1}{\Gamma (\omega )}\int \limits _{0}^{\tau }(\tau -{\mathfrak {p}})^{\omega -1}{\mathcal {Q}}_{1}\big ({\mathfrak {p}},{\textbf{S}}\big )d{\mathfrak {p}},\\ {{\textbf{L}}}_{{\textbf{T}}}(\tau )-{{\textbf{L}}}_{{\textbf{T}}}(0)=\frac{1}{\Gamma (\omega )}\int \limits _{0}^{\tau }(\tau -{\mathfrak {p}})^{\omega -1}{\mathcal {Q}}_{2}\big ({\mathfrak {p}},{{\textbf{L}}}_{{\textbf{T}}}\big )d{\mathfrak {p}},\\ {{\textbf{I}}}_{{\textbf{T}}}(\tau )-{{\textbf{I}}}_{{\textbf{T}}}(0)=\frac{1}{\Gamma (\omega )}\int \limits _{0}^{\tau }(\tau -{\mathfrak {p}})^{\omega -1}{\mathcal {Q}}_{3}\big ({\mathfrak {p}},{{\textbf{I}}}_{{\textbf{T}}}\big )d{\mathfrak {p}},\\ {{\textbf{E}}}_{{\textbf{C}}}(\tau )-{{\textbf{E}}}_{{\textbf{C}}}(0)=\frac{1}{\Gamma (\omega )}\int \limits _{0}^{\tau }(\tau -{\mathfrak {p}})^{\omega -1}{\mathcal {Q}}_{4}\big ({\mathfrak {p}},{{\textbf{E}}}_{{\textbf{C}}}\big )d{\mathfrak {p}},\\ {{\textbf{I}}}_{{\textbf{C}}}(\tau )-{{\textbf{I}}}_{{\textbf{C}}}(0)=\frac{1}{\Gamma (\omega )}\int \limits _{0}^{\tau }(\tau -{\mathfrak {p}})^{\omega -1}{\mathcal {Q}}_{5}\big ({\mathfrak {p}},{{\textbf{E}}}_{{\textbf{C}}}\big )d{\mathfrak {p}},\\ {{\textbf{L}}}_{{\textbf{T}}{\textbf{C}}}(\tau )-{{\textbf{L}}}_{{\textbf{T}}{\textbf{C}}}(0)=\frac{1}{\Gamma (\omega )}\int \limits _{0}^{\tau }(\tau -{\mathfrak {p}})^{\omega -1}{\mathcal {Q}}_{6}\big ({\mathfrak {p}},{{\textbf{L}}}_{{\textbf{T}}{\textbf{C}}}\big )d{\mathfrak {p}},\\ {{\textbf{I}}}_{{\textbf{T}}{\textbf{C}}}(\tau )-{{\textbf{I}}}_{{\textbf{T}}{\textbf{C}}}(0)=\frac{1}{\Gamma (\omega )}\int \limits _{0}^{\tau }(\tau -{\mathfrak {p}})^{\omega -1}{\mathcal {Q}}_{7}\big ({\mathfrak {p}},{{\textbf{I}}}_{{\textbf{T}}{\textbf{C}}}\big )d{\mathfrak {p}},\\ {{\textbf{R}}}(\tau )-{{\textbf{R}}}(0)=\frac{1}{\Gamma (\omega )}\int \limits _{0}^{\tau }(\tau -{\mathfrak {p}})^{\omega -1}{\mathcal {Q}}_{8}\big ({\mathfrak {p}},{{\textbf{R}}}\big )d{\mathfrak {p}}.\\ \end{array}\right. } \end{aligned}$$

The kernels ${\mathcal {Q}}_{\iota },~(\iota =1,...,8)$ satisfies the Lipschitz condition and contraction, as demonstrated.

#### Theorem 3

${\mathcal {Q}}_{1}$ satisfies the Lipschitz condition and contraction if the following condition holds: $0\le \alpha _{1}(\sigma _{3}+\sigma _{7})+\alpha _{2}(\sigma _{4}+\sigma _{5}+\sigma _{2}+\sigma _{5})+\beta <1.$

#### Proof

For ${\textbf{S}}$ and $\mathbf {S_{1}},$ we have

$$\begin{aligned} \big \Vert {\mathcal {Q}}_{1}\big (\tau ,{{\textbf{S}}}\big )-{\mathcal {Q}}_{1}\big (\tau ,{{\textbf{S}}}_{1}\big )\big \Vert{} & {} =\big \Vert \big (\alpha _{1}({\textbf{I}}_{{\textbf{T}}}+{\textbf{I}}_{\textbf{TC}})+\alpha _{2}({\textbf{E}}_{{\textbf{C}}}+{\textbf{I}}_{{\textbf{C}}}+{\textbf{I}}_{\textbf{TC}}+{\textbf{L}}_{{\textbf{T}}})+\beta \big )\big ({{\textbf{S}}}(\tau )-{{\textbf{S}}}_{1}(\tau )\big )\big \Vert \nonumber \\ {}{} & {} \le \big (\alpha _{1}\big (\big \Vert {\textbf{I}}_{{\textbf{T}}}\big \Vert +\big \Vert {\textbf{I}}_{\textbf{TC}})\big \Vert \big )+\alpha _{2}\big (\big \Vert {\textbf{E}}_{{\textbf{C}}}\big \Vert +\big \Vert {\textbf{I}}_{{\textbf{C}}}\big \Vert +\big \Vert {\textbf{I}}_{\textbf{TC}}\big \Vert +\big \Vert {\textbf{L}}_{{\textbf{T}}}\big \Vert \big )+\beta \big )\big \Vert {{\textbf{S}}}(\tau )-{{\textbf{S}}}_{1}(\tau )\big \Vert . \end{aligned}$$

Suppose ${\mathcal {V}}_{1}=\alpha _{1}(\sigma _{3}+\sigma _{7})+\alpha _{2}(\sigma _{4}+\sigma _{5}+\sigma _{2}+\sigma _{5})+\beta$, where ${\textbf{I}}_{{\textbf{T}}}\le \sigma _{3},~{\textbf{I}}_{\textbf{TC}}\le \sigma _{7},~{\textbf{E}}_{{\textbf{C}}}\le \sigma _{4}~{\textbf{I}}_{{\textbf{C}}}\le \sigma _{5},~{\textbf{I}}_{\textbf{TC}}\le \sigma _{7},~{\textbf{L}}_{{\textbf{T}}}\le \sigma _{2}$ are a bounded functions. So, we have

15 $$\begin{aligned} \big \Vert {\mathcal {Q}}_{1}\big (\tau ,{{\textbf{S}}}\big )-{\mathcal {Q}}_{1}\big (\tau ,{{\textbf{S}}}_{1}\big )\big \Vert \le {\mathcal {V}}_{1}\big \Vert {{\textbf{S}}}(\tau )-{{\textbf{S}}}_{1}(\tau )\big \Vert . \end{aligned}$$

After obtaining the Lipschitz criterion for ${\mathcal {Q}}_{1}$, hence, ${\mathcal {Q}}_{1}$ is a contraction if $0\le \alpha _{1}(\sigma _{3}+\sigma _{7})+\alpha _{2}(\sigma _{4}+\sigma _{5}+\sigma _{2}+\sigma _{5})+\beta <1$.

In the same manner, ${\mathcal {Q}}_{\jmath }~(\jmath =2,..,7)$ satisfy the Lipschitz condition as follows:

$$\begin{aligned}{} & {} \big \Vert {\mathcal {Q}}_{2}\big (\tau ,{{\textbf{L}}}_{{\textbf{T}}}\big )-{\mathcal {Q}}_{2}\big (\tau ,{{{\textbf{L}}}_{{\textbf{T}}}}_{1}\big )\big \Vert \le {\mathcal {V}}_{2}\big \Vert {{\textbf{L}}}_{{\textbf{T}}}(\tau )-{{{\textbf{L}}}_{{\textbf{T}}}}_{1}(\tau )\big \Vert ,\nonumber \\ {}{} & {} \big \Vert {\mathcal {Q}}_{3}\big (\tau ,{{\textbf{I}}}_{{\textbf{T}}}\big )-{\mathcal {Q}}_{3}\big (\tau ,{{{\textbf{I}}}_{{\textbf{T}}}}_{1}\big )\big \Vert \le {\mathcal {V}}_{3}\big \Vert {{\textbf{I}}}_{{\textbf{T}}}(\tau )-{{{\textbf{I}}}_{{\textbf{T}}}}_{1}(\tau )\big \Vert ,\nonumber \\ {}{} & {} \big \Vert {\mathcal {Q}}_{4}\big (\tau ,{{\textbf{E}}}_{{\textbf{C}}}\big )-{\mathcal {Q}}_{4}\big (\tau ,{{{\textbf{E}}}_{{\textbf{C}}}}_{1}\big )\big \Vert \le {\mathcal {V}}_{4}\big \Vert {{\textbf{E}}}_{{\textbf{C}}}(\tau )-{{{\textbf{E}}}_{{\textbf{C}}}}_{1}(\tau )\big \Vert ,\nonumber \\ {}{} & {} \big \Vert {\mathcal {Q}}_{5}\big (\tau ,{{\textbf{I}}}_{{\textbf{C}}}\big )-{\mathcal {Q}}_{5}\big (\tau ,{{{\textbf{I}}}_{{\textbf{C}}}}_{1}\big )\big \Vert \le {\mathcal {V}}_{5}\big \Vert {{\textbf{L}}}_{{\textbf{T}}}(\tau )-{{{\textbf{I}}}_{{\textbf{C}}}}_{1}(\tau )\big \Vert ,\nonumber \\ {}{} & {} \big \Vert {\mathcal {Q}}_{6}\big (\tau ,{{\textbf{L}}}_{\textbf{TC}}\big )-{\mathcal {Q}}_{6}\big (\tau ,{{{\textbf{L}}}_{\textbf{TC}}}_{1}\big )\big \Vert \le {\mathcal {V}}_{6}\big \Vert {{\textbf{S}}}(\tau )-{{{\textbf{L}}}_{\textbf{TC}}}_{1}(\tau )\big \Vert ,\nonumber \\ {}{} & {} \big \Vert {\mathcal {Q}}_{7}\big (\tau ,{{\textbf{I}}}_{\textbf{TC}}\big )-{\mathcal {Q}}_{T}\big (\tau ,{{{\textbf{I}}}_{\textbf{TC}}}_{1}\big )\big \Vert \le {\mathcal {V}}_{7}\big \Vert {{\textbf{S}}}(\tau )-{{\textbf{S}}}_{1}(\tau )\big \Vert ,\nonumber \\ {}{} & {} \big \Vert {\mathcal {Q}}_{8}\big (\tau ,{{\textbf{r}}}\big )-{\mathcal {Q}}_{8}\big (\tau ,{{\textbf{R}}}_{1}\big )\big \Vert \le {\mathcal {V}}_{8}\big \Vert {{\textbf{R}}}(\tau )-{{\textbf{R}}}_{1}(\tau )\big \Vert ,\end{aligned}$$

where $${\mathcal {V}}_{2}=\psi _{{\textbf{T}}}\sigma _{1}-(\beta +\mu +\lambda \psi _{{\textbf{C}}}+\varpi ),~{\mathcal {V}}_{3}=\mu \sigma _{2}+\varsigma _{2}\eta \sigma _{6}+\theta _{2}\xi \sigma _{7}-(\beta +\varsigma _{3}+\zeta _{{\textbf{T}}}+\delta ),~{\mathcal {V}}_{4}=\psi _{{\textbf{C}}}\sigma _{1}-(\beta +\epsilon \psi _{{\textbf{T}}}+\varphi _{1}+\varphi _{2}),~{\mathcal {V}}_{5}=\varphi _{1}\sigma _{4}+\rho \eta \sigma _{6}+\theta _{1}\xi \sigma _{7}-(\beta +\zeta _{{\textbf{C}}}+\nu +\varphi _{3}),~{\mathcal {V}}_{6}=\lambda \psi _{{\textbf{C}}}\sigma _{2}+\epsilon \psi _{{\textbf{T}}}\sigma _{4}-(\beta +\zeta _{{\textbf{C}}}+\rho +\xi ),~{\mathcal {V}}_{7}=\rho \sigma _{6}+\varsigma _{3}\sigma _{3}+\nu \sigma _{5}-(\beta +\zeta _{\textbf{TC}}+\xi ),~{\mathcal {V}}_{8}=\varpi \sigma _{2}+\varphi _{2}\sigma _{4}+\delta \sigma _{3}+\varphi _{3}\sigma _{5}+(1-(\varsigma _{1}+\varsigma _{2}))\eta \sigma _{6}+(1-(\theta _{1}+\theta _{2}))\xi \sigma _{7}-\beta .$$

For $\jmath =2,...,8,$ we find $0\le {\mathcal {V}}_{\jmath }<1,$ then ${\mathcal {V}}_{\jmath }$ are contractions. Assume the following recursive pattern, as suggested by system (15):

$$\begin{aligned}{\left\{ \begin{array}{ll} \Theta _{1n}(\tau )={\textbf{S}}_{n}(\tau )-{\textbf{S}}_{n-1}(\tau )=\frac{1}{\Gamma (\omega )}\int \limits _{0}^{\tau }(\tau -{\mathfrak {p}})^{\omega -1}\big ({\mathcal {Q}}_{1}\big ({\mathfrak {p}},{\textbf{S}}_{n-1})-{\mathcal {Q}}_{1}\big ({\mathfrak {p}},{\textbf{S}}_{n-2})\big )d{\mathfrak {p}},\\ \Theta _{2n}(\tau )={{{\textbf{L}}}_{{\textbf{T}}}}_{n}(\tau )-{{{\textbf{L}}}_{{\textbf{T}}}}_{n-1}(\tau )=\frac{1}{\Gamma (\omega )}\int \limits _{0}^{\tau }(\tau -{\mathfrak {p}})^{\omega -1}\big ({\mathcal {Q}}_{2}\big ({\mathfrak {p}},{{{\textbf{L}}}_{{\textbf{T}}}}_{n-1})-{\mathcal {Q}}_{2}\big ({\mathfrak {p}},{{{\textbf{L}}}_{{\textbf{T}}}}_{n-2})\big )d{\mathfrak {p}},\\ \Theta _{3n}(\tau )={{{\textbf{I}}}_{{\textbf{T}}}}_{n}(\tau )-{{{\textbf{I}}}_{{\textbf{T}}}}_{n-1}(\tau )=\frac{1}{\Gamma (\omega )}\int \limits _{0}^{\tau }(\tau -{\mathfrak {p}})^{\omega -1}\big ({\mathcal {Q}}_{3}\big ({\mathfrak {p}},{{{\textbf{I}}}_{{\textbf{T}}}}_{n-1})-{\mathcal {Q}}_{3}\big ({\mathfrak {p}},{{{\textbf{I}}}_{{\textbf{T}}}}_{n-2})\big )d{\mathfrak {p}},\\ \Theta _{4n}(\tau )={{{\textbf{E}}}_{{\textbf{C}}}}_{n}(\tau )-{{{\textbf{E}}}_{{\textbf{C}}}}_{n-1}(\tau )=\frac{1}{\Gamma (\omega )}\int \limits _{0}^{\tau }(\tau -{\mathfrak {p}})^{\omega -1}\big ({\mathcal {Q}}_{4}\big ({\mathfrak {p}},{{{\textbf{E}}}_{{\textbf{C}}}}_{n-1})-{\mathcal {Q}}_{4}\big ({\mathfrak {p}},{{{\textbf{E}}}_{{\textbf{C}}}}_{n-2})\big )d{\mathfrak {p}},\\ \Theta _{5n}(\tau )={{{\textbf{I}}}_{{\textbf{C}}}}_{n}(\tau )-{{{\textbf{I}}}_{{\textbf{C}}}}_{n-1}(\tau )=\frac{1}{\Gamma (\omega )}\int \limits _{0}^{\tau }(\tau -{\mathfrak {p}})^{\omega -1}\big ({\mathcal {Q}}_{5}\big ({\mathfrak {p}},{{{\textbf{I}}}_{{\textbf{C}}}}_{n-1})-{\mathcal {Q}}_{5}\big ({\mathfrak {p}},{{{\textbf{I}}}_{{\textbf{C}}}}_{n-2})\big )d{\mathfrak {p}},\\ \Theta _{6n}(\tau )={{{\textbf{L}}}_{\textbf{TC}}}_{n}(\tau )-{{{\textbf{L}}}_{\textbf{TC}}}_{n-1}(\tau )=\frac{1}{\Gamma (\omega )}\int \limits _{0}^{\tau }(\tau -{\mathfrak {p}})^{\omega -1}\big ({\mathcal {Q}}_{6}\big ({\mathfrak {p}},{{{\textbf{L}}}_{\textbf{TC}}}_{n-1})-{\mathcal {Q}}_{6}\big ({\mathfrak {p}},{{{\textbf{L}}}_{\textbf{TC}}}_{n-2})\big )d{\mathfrak {p}},\\ \Theta _{7n}(\tau )={{{\textbf{I}}}_{\textbf{TC}}}_{n}(\tau )-{{{\textbf{I}}}_{\textbf{TC}}}_{n-1}(\tau )=\frac{1}{\Gamma (\omega )}\int \limits _{0}^{\tau }(\tau -{\mathfrak {p}})^{\omega -1}\big ({\mathcal {Q}}_{7}\big ({\mathfrak {p}},{{{\textbf{I}}}_{\textbf{TC}}}_{n-1})-{\mathcal {Q}}_{7}\big ({\mathfrak {p}},{{{\textbf{I}}}_{\textbf{TC}}}_{n-2})\big )d{\mathfrak {p}},\\ \Theta _{8n}(\tau )={{{\textbf{R}}}}_{n}(\tau )-{{{\textbf{R}}}}_{n-1}(\tau )=\frac{1}{\Gamma (\omega )}\int \limits _{0}^{\tau }(\tau -{\mathfrak {p}})^{\omega -1}\big ({\mathcal {Q}}_{8}\big ({\mathfrak {p}},{{{\textbf{R}}}}_{n-1})-{\mathcal {Q}}_{8}\big ({\mathfrak {p}},{{{\textbf{R}}}}_{n-2})\big )d{\mathfrak {p}}, \end{array}\right. } \end{aligned}$$

with $${{\textbf{S}}}(0)\ge 0,~{{\textbf{L}}}_{{\textbf{T}}}(0)\ge 0,~{{\textbf{I}}}_{{\textbf{T}}}(0)\ge 0,~{{\textbf{E}}}_{{\textbf{C}}}(0)\ge 0,~{{\textbf{I}}}_{{\textbf{C}}}(0)\ge 0,~{{\textbf{L}}}_{{\textbf{T}}{\textbf{C}}}(0)\ge 0,~{{\textbf{I}}}_{{\textbf{T}}{\textbf{C}}}(0)\ge 0,~{{\textbf{R}}}(0)\ge 0.$$

Throughout the above system, we compute the norm of its first equation and then

$$\begin{aligned} \big \Vert \Theta _{1n}(\tau )\big \Vert{} & {} =\big \Vert {\textbf{S}}_{n}(\tau )-{\textbf{S}}_{n-1}(\tau )\big \Vert =\bigg \Vert \frac{1}{\Gamma (\omega )}\int \limits _{0}^{\tau }(\tau -{\mathfrak {p}})^{\omega -1}\big ({\mathcal {Q}}_{1}\big ({\mathfrak {p}},{\textbf{S}}_{n-1})-{\mathcal {Q}}_{1}\big ({\mathfrak {p}},{\textbf{S}}_{n-2})\big )d{\mathfrak {p}}\bigg \Vert \nonumber \\ {}{} & {} \le \frac{1}{\Gamma (\omega )}\int \limits _{0}^{\tau }\big \Vert (\tau -{\mathfrak {p}})^{\omega -1}\big ({\mathcal {Q}}_{1}\big ({\mathfrak {p}},{\textbf{S}}_{n-1})-{\mathcal {Q}}_{1}\big ({\mathfrak {p}},{\textbf{S}}_{n-2})\big )\big \Vert d{\mathfrak {p}}. \end{aligned}$$

Therefore, (16) possesses Lipschitz’s condition, then we have

$$\begin{aligned} \big \Vert \Theta _{1n}(\tau )\big \Vert \le \frac{{\mathcal {V}}_{1}}{\Gamma (\omega )}\int \limits _{0}^{\tau }\big \Vert \Theta _{1(n-1)}({\mathfrak {p}})\big \Vert d{\mathfrak {p}}. \end{aligned}$$

Analogously, we find

16 $$\begin{aligned}{} & {} \big \Vert \Theta _{2n}(\tau )\big \Vert \le \frac{{\mathcal {V}}_{2}}{\Gamma (\omega )}\int \limits _{0}^{\tau }\big \Vert \Theta _{2(n-1)}({\mathfrak {p}})\big \Vert d{\mathfrak {p}},\nonumber \\ {}{} & {} \big \Vert \Theta _{3n}(\tau )\big \Vert \le \frac{{\mathcal {V}}_{3}}{\Gamma (\omega )}\int \limits _{0}^{\tau }\big \Vert \Theta _{3(n-1)}({\mathfrak {p}})\big \Vert d{\mathfrak {p}},\nonumber \\ {}{} & {} \big \Vert \Theta _{4n}(\tau )\big \Vert \le \frac{{\mathcal {V}}_{4}}{\Gamma (\omega )}\int \limits _{0}^{\tau }\big \Vert \Theta _{4(n-1)}({\mathfrak {p}})\big \Vert d{\mathfrak {p}},\nonumber \\ {}{} & {} \big \Vert \Theta _{5n}(\tau )\big \Vert \le \frac{{\mathcal {V}}_{5}}{\Gamma (\omega )}\int \limits _{0}^{\tau }\big \Vert \Theta _{5(n-1)}({\mathfrak {p}})\big \Vert d{\mathfrak {p}},\nonumber \\ {}{} & {} \big \Vert \Theta _{6n}(\tau )\big \Vert \le \frac{{\mathcal {V}}_{6}}{\Gamma (\omega )}\int \limits _{0}^{\tau }\big \Vert \Theta _{6(n-1)}({\mathfrak {p}})\big \Vert d{\mathfrak {p}},\nonumber \\ {}{} & {} \big \Vert \Theta _{7n}(\tau )\big \Vert \le \frac{{\mathcal {V}}_{7}}{\Gamma (\omega )}\int \limits _{0}^{\tau }\big \Vert \Theta _{7(n-1)}({\mathfrak {p}})\big \Vert d{\mathfrak {p}},\nonumber \\ {}{} & {} \big \Vert \Theta _{8n}(\tau )\big \Vert \le \frac{{\mathcal {V}}_{8}}{\Gamma (\omega )}\int \limits _{0}^{\tau }\big \Vert \Theta _{8(n-1)}({\mathfrak {p}})\big \Vert d{\mathfrak {p}}.\end{aligned}$$

As a consequence, we can write

$$\begin{aligned}{} & {} {\textbf{S}}_{n}(\tau )=\sum \limits _{\iota =1}^{\infty }\Theta _{1\iota }(\tau ),~~{{\textbf{L}}_{{\textbf{T}}}}_{n}(\tau )=\sum \limits _{\iota =1}^{\infty }\Theta _{2\iota }(\tau ),~~{{\textbf{I}}_{{\textbf{T}}}}_{n}(\tau )=\sum \limits _{\iota =1}^{\infty }\Theta _{3\iota }(\tau ),~~{{\textbf{E}}_{{\textbf{C}}}}_{n}(\tau )=\sum \limits _{\iota =1}^{\infty }\Theta _{4\iota }(\tau ),~~{{\textbf{I}}_{{\textbf{C}}}}_{n}(\tau )=\sum \limits _{\iota =1}^{\infty }\Theta _{5\iota }(\tau ),\nonumber \\ {}{} & {} ~~{{\textbf{L}}_{\textbf{TC}}}_{n}(\tau )=\sum \limits _{\iota =1}^{\infty }\Theta _{6\iota }(\tau ),~~{{\textbf{L}}_{\textbf{TC}}}_{n}(\tau )=\sum \limits _{\iota =1}^{\infty }\Theta _{7\iota }(\tau ),~~{\textbf{R}}_{n}(\tau )=\sum \limits _{\iota =1}^{\infty }\Theta _{8\iota }(\tau ). \end{aligned}$$

$$\square $$

#### Theorem 4

A system of solutions described by the codynamics model (3) exists if there exists $\tau _{1}$ such that $\Big (\frac{\tau _{1}{\mathcal {V}}_{\jmath }}{\Gamma (\omega )}\Big )<1,~(\jmath =1,...,8).$

#### Proof

By means of (16) and (17), we have

$$\begin{aligned}{} & {} \big \Vert \Theta _{1n}(\tau )\big \Vert \le \big \Vert {\textbf{S}}_{n}(0)\big \Vert \Big (\frac{{\mathcal {V}}_{1}\tau }{\Gamma (\omega )}\Big )^{n},~~ \big \Vert \Theta _{2n}(\tau )\big \Vert \le \big \Vert {{\textbf{L}}_{{\textbf{T}}}}_{n}(0)\big \Vert \Big (\frac{{\mathcal {V}}_{2}\tau }{\Gamma (\omega )}\Big )^{n},~~ \big \Vert \Theta _{3n}(\tau )\big \Vert \le \big \Vert {{\textbf{I}}_{{\textbf{T}}}}_{n}(0)\big \Vert \Big (\frac{{\mathcal {V}}_{3}\tau }{\Gamma (\omega )}\Big )^{n},\nonumber \\ {}{} & {} \big \Vert \Theta _{4n}(\tau )\big \Vert \le \big \Vert {{\textbf{E}}_{{\textbf{C}}}}_{n}(0)\big \Vert \Big (\frac{{\mathcal {V}}_{4}\tau }{\Gamma (\omega )}\Big )^{n},~~ \big \Vert \Theta _{5n}(\tau )\big \Vert \le \big \Vert {{\textbf{I}}_{{\textbf{C}}}}_{n}(0)\big \Vert \Big (\frac{{\mathcal {V}}_{5}\tau }{\Gamma (\omega )}\Big )^{n},~~ \big \Vert \Theta _{6n}(\tau )\big \Vert \le \big \Vert {{\textbf{L}}_{\textbf{TC}}}_{n}(0)\big \Vert \Big (\frac{{\mathcal {V}}_{6}\tau }{\Gamma (\omega )}\Big )^{n},\nonumber \\ {}{} & {} \big \Vert \Theta _{7n}(\tau )\big \Vert \le \big \Vert {{\textbf{I}}_{\textbf{TC}}}_{n}(0)\big \Vert \Big (\frac{{\mathcal {V}}_{7}\tau }{\Gamma (\omega )}\Big )^{n},~~ \big \Vert \Theta _{8n}(\tau )\big \Vert \le \big \Vert {{\textbf{R}}}_{n}(0)\big \Vert \Big (\frac{{\mathcal {V}}_{8}\tau }{\Gamma (\omega )}\Big )^{n}.\end{aligned}$$

Thus, the system is continuous and has a solution. Now we shall explain how the functions listed above may be used to construct a model solution (15). We make the assumption that

$$\begin{aligned}{} & {} {\textbf{S}}(\tau )-{\textbf{S}}(0)={{\textbf{S}}}_{n}(\tau )-{\tilde{\Theta }}_{1n}(\tau ),~~~{{\textbf{L}}}_{{\textbf{T}}}(\tau )-{{\textbf{L}}}_{{\textbf{T}}}(0)={{{\textbf{L}}}_{{\textbf{T}}}}_{n}(\tau )-{\tilde{\Theta }}_{2n}(\tau ),\nonumber \\{} & {} {{\textbf{I}}}_{{\textbf{T}}}(\tau )-{{\textbf{I}}}_{{\textbf{T}}}(0)={{{\textbf{I}}}_{{\textbf{T}}}}_{n}(\tau )-{\tilde{\Theta }}_{3n}(\tau ),~~~{{\textbf{E}}}_{{\textbf{C}}}(\tau )-{{\textbf{E}}}_{{\textbf{C}}}(0)={{{\textbf{E}}}_{{\textbf{C}}}}_{n}(\tau )-{\tilde{\Theta }}_{4n}(\tau ),\nonumber \\{} & {} {{\textbf{I}}}_{{\textbf{C}}}(\tau )-{{\textbf{I}}}_{{\textbf{C}}}(0)={{{\textbf{I}}}_{{\textbf{C}}}}_{n}(\tau )-{\tilde{\Theta }}_{5n}(\tau ),~~~{{\textbf{L}}}_{\textbf{TC}}(\tau )-{{\textbf{L}}}_{\textbf{TC}}(0)={{{\textbf{L}}}_{\textbf{TC}}}_{n}(\tau )-{\tilde{\Theta }}_{6n}(\tau ),\nonumber \\{} & {} {{\textbf{I}}}_{\textbf{TC}}(\tau )-{{\textbf{I}}}_{\textbf{TC}}(0)={{{\textbf{I}}}_{\textbf{TC}}}_{n}(\tau )-{\tilde{\Theta }}_{7n}(\tau ),~~~{{\textbf{R}}}(\tau )-{{\textbf{R}}}(0)={{{\textbf{R}}}}_{n}(\tau )-{\tilde{\Theta }}_{6n}(\tau ). \end{aligned}$$

Therefore, we have

$$\begin{aligned} \big \Vert {\tilde{\Theta }}_{1n}(\tau ) \big \Vert{} & {} =\bigg \Vert \frac{1}{\Gamma (\omega )}\int \limits _{0}^{\tau }\big ({\mathcal {Q}}_{1}({\mathfrak {p}},{\textbf{S}})-{\mathcal {Q}}_{1}({\mathfrak {p}},{\textbf{S}}_{n-1})\big )d{\mathfrak {p}}\bigg \Vert \nonumber \\ {}{} & {} \le \frac{1}{\Gamma (\omega )}\int \limits _{0}^{\tau }\big \Vert {\mathcal {Q}}_{1}({\mathfrak {p}},{\textbf{S}})-{\mathcal {Q}}_{1}({\mathfrak {p}},{\textbf{S}}_{n-1})\big \Vert d{\mathfrak {p}}\nonumber \\ {}{} & {} \le \frac{\tau {\mathcal {V}}_{1}}{\Gamma (\omega )}\big \Vert {\textbf{S}}-{\textbf{S}}_{n-1}\big \Vert . \end{aligned}$$

After recursive procedure, we have the following:

$$\begin{aligned} \big \Vert {\tilde{\Theta }}_{1n}(\tau )\big \Vert \le \bigg (\frac{\tau {\mathcal {V}}_{1}}{\Gamma (\omega )}\bigg )\mho . \end{aligned}$$

Thus, $$\big \Vert {\tilde{\Theta }}_{1n}(\tau )\big \Vert \mapsto 0~~~~as~~~n\mapsto \infty .$$

Similarly, we may establish that $\big \Vert {\tilde{\Theta }}_{\jmath n}(\tau )\big \Vert \mapsto 0,~~(\jmath =2,...,8)~as~n\mapsto \infty .$

To examine the uniqueness of the solution, we assume that there is another solution of the system, such as ${\textbf{S}}_{1}(\tau ),{{{\textbf{L}}}_{{\textbf{T}}}}_{1}(\tau ),~{{{\textbf{I}}}_{{\textbf{T}}}}_{1}(\tau ),~{{{\textbf{E}}}_{{\textbf{C}}}}_{1}(\tau ),~{{{\textbf{I}}}_{{\textbf{C}}}}_{1}(\tau ),~{{{\textbf{L}}}_{\textbf{TC}}}_{1}(\tau ),~{{{\textbf{I}}}_{\textbf{TC}}}_{1}(\tau )~ and~{{\textbf{R}}}_{1}(\tau ).$ Then

$$\begin{aligned} {\textbf{S}}(\tau )-{\textbf{S}}_{1}(\tau )=\frac{1}{\Gamma (\omega )}\int \limits _{0}^{\tau }\big ({\mathcal {Q}}_{1}({\mathfrak {p}},{\textbf{S}})-{\mathcal {Q}}_{1}({\mathfrak {p}},{\textbf{S}}_{n-1})\big )d{\mathfrak {p}}. \end{aligned}$$

After taking norm, we get

$$\begin{aligned} \big \Vert {\textbf{S}}(\tau )-{\textbf{S}}_{1}(\tau )\big \Vert \le \frac{1}{\Gamma (\omega )}\int \limits _{0}^{\tau }\big \Vert {\mathcal {Q}}_{1}({\mathfrak {p}},{\textbf{S}})-{\mathcal {Q}}_{1}({\mathfrak {p}},{\textbf{S}}_{n-1})\big \Vert d{\mathfrak {p}}. \end{aligned}$$

Utilizing the Lipschitz condition, we have

$$\begin{aligned} \big \Vert {\textbf{S}}(\tau )-{\textbf{S}}_{1}(\tau )\big \Vert \le \frac{\tau {\mathcal {V}}_{1}}{\Gamma (\omega )}\big \Vert {\textbf{S}}-{\textbf{S}}_{n-1}\big \Vert . \end{aligned}$$

Consequently, we have

17 $$\begin{aligned} \big \Vert {\textbf{S}}(\tau )-{\textbf{S}}_{1}(\tau )\big \Vert \Big (1-\frac{\tau {\mathcal {V}}_{1}}{\Gamma (\omega )}\Big )\le 0. \end{aligned}$$

$$\square $$

#### Theorem 5

The codynamics model (3) has a unique solution, provided that $\Big (1-\frac{\tau {\mathcal {V}}_{1}}{\Gamma (\omega )}\Big )>0.$

#### Proof

Assuming that condition (18) is vaild,

$$\begin{aligned} \big \Vert {\textbf{S}}(\tau )-{\textbf{S}}_{1}(\tau )\big \Vert \Big (1-\frac{\tau {\mathcal {V}}_{1}}{\Gamma (\omega )}\Big )\le 0. \end{aligned}$$

Then $\big \Vert {\textbf{S}}(\tau )-{\textbf{S}}_{1}(\tau )\big \Vert =0.$ Hence, we have ${\textbf{S}}(\tau )={\textbf{S}}_{1}(\tau ).$ Similarly, we can prove that ${{\textbf{L}}}_{{\textbf{T}}}(\tau )={{{\textbf{L}}}_{{\textbf{T}}}}_{1}(\tau ),~{{\textbf{I}}}_{{\textbf{T}}}(\tau )={{{\textbf{I}}}_{{\textbf{T}}}}_{1}(\tau ),~{{\textbf{E}}}_{{\textbf{C}}}(\tau )={{{\textbf{E}}}_{{\textbf{C}}}}_{1}(\tau ),~{{\textbf{I}}}_{{\textbf{C}}}(\tau )={{{\textbf{I}}}_{{\textbf{C}}}}_{1}(\tau ),~{{\textbf{L}}}_{\textbf{TC}}(\tau )={{{\textbf{L}}}_{\textbf{TC}}}_{1}(\tau ),~{{\textbf{I}}}_{\textbf{TC}}(\tau )={{{\textbf{I}}}_{\textbf{TC}}}_{1}(\tau ),~{{\textbf{R}}}(\tau )={{\textbf{R}}}_{1}(\tau ).$ $\square$

### Influence of TB on COVID-19

We started by describing the basic reproductive quantity, ${\mathbb {R}}_{0}^{{\mathbb {C}}}$, by means of ${\mathbb {R}}_{0}^{{\mathbb {T}}}$ (and vice versa), in order to examine the effect of TB illness on COVID-19 (and vice versa)^45^. By interpreting the value of $\beta$ as a component of ${\mathbb {R}}_{0}^{{\mathbb {T}}}$ using the formula (2), we get

18 $$\begin{aligned} {\mathbb {R}}_{0}^{{\mathbb {T}}}=\frac{\mu \alpha _{1}}{(\beta +\mu +\varpi )(\beta +\zeta _{{\textbf{T}}}+\delta )}. \end{aligned}$$

Now, we have

19 $$\begin{aligned} {\mathbb {R}}_{0}^{{\mathbb {C}}}=\frac{\alpha _{2}{\mathbb {R}}_{0}^{{\mathbb {T}}}\big ({\mathbb {R}}_{0}^{{\mathbb {T}}}(\zeta _{{\textbf{C}}}+\varphi _{1}+\varphi _{3})-{\mathcal {K}}_{8}/2+\sqrt{{\mathbb {R}}_{0}^{T}\big ({\mathbb {R}}_{0}^{T}{\mathcal {K}}_{8}^{2}+4\mu \alpha _{1}\big )/2\big )}}{\big (\sqrt{{\mathbb {R}}_{0}^{T}\big ({\mathbb {R}}_{0}^{T}{\mathcal {K}}_{8}^{2}+4\mu \alpha _{1}\big )/2}+(\varphi _{1}+\varphi _{3}){\mathbb {R}}_{0}^{{\mathbb {T}}}-{\mathcal {K}}_{8}{\mathbb {R}}_{0}^{{\mathbb {T}}}/2\big )\big (\sqrt{{\mathbb {R}}_{0}^{T}\big ({\mathbb {R}}_{0}^{T}{\mathcal {K}}_{8}^{2}+4\mu \alpha _{1}\big )/2}+(\zeta _{{\textbf{C}}}+\varphi _{3}){\mathbb {R}}_{0}^{{\mathbb {T}}}-{\mathcal {K}}_{8}{\mathbb {R}}_{0}^{{\mathbb {T}}}/2\big )}, \end{aligned}$$

where ${\mathcal {K}}_{8}=(\mu +\varpi +\zeta _{{\textbf{T}}}+\delta ).$ Furthermore, the $\frac{\partial {\mathbb {R}}_{0}^{{\mathbb {C}}}}{\partial {\mathbb {R}}_{0}^{{\mathbb {T}}}}>0,$ it also indicates that the COVID-19 outbreak is made worse by the spread of TB viruses.

#### Remark 1

The population’s TB proliferation possesses no noticeable influence with the propagation of COVID-19 provided $\frac{\partial {\mathbb {R}}_{0}^{{\mathbb {C}}}}{\partial {\mathbb {R}}_{0}^{{\mathbb {T}}}}=0$. On the other hand, the transmission of COVID-19 will be significantly adversely affected by the outbreak of TB if $\frac{\partial {\mathbb {R}}_{0}^{{\mathbb {C}}}}{\partial {\mathbb {R}}_{0}^{{\mathbb {T}}}}<0$.

Furthermore, by quantifying ${\mathbb {R}}_{0}^{{\mathbb {T}}}$ in the context of ${\mathbb {R}}_{0}^{{\mathbb {C}}}$ and determining the meaning of the partial derivative of ${\mathbb {R}}_{0}^{{\mathbb {C}}}$ with regard to ${\mathbb {R}}_{0}^{{\mathbb {T}}}$, the effect of COVID-19 of TB infections is able to be rectified.

### Analysis of COVID-19

When the infection of TB is disregarded, the deterministic model (2) becomes the subsequent system:

20 $$\begin{aligned} {\left\{ \begin{array}{ll} \dot{{\textbf{S}}}=\nabla -\frac{\alpha _{2}}{{\mathcal {N}}_{1}}({{\textbf{E}}}_{{\textbf{C}}}+{{\textbf{I}}}_{{\textbf{C}}})-\beta {{\textbf{S}}}={\widehat{\Phi }}_{1},\\ \dot{{{\textbf{E}}}_{{\textbf{C}}}}=\frac{\alpha _{2}}{{\mathcal {N}}_{1}}({{\textbf{E}}}_{{\textbf{C}}}+{{\textbf{I}}}_{{\textbf{C}}})-(\beta +\varphi _{1}+\varphi _{2}){{\textbf{E}}}_{{\textbf{C}}}={\widehat{\Phi }}_{2},\\ \dot{{{\textbf{I}}}_{{\textbf{C}}}}=\varphi _{1}{{\textbf{E}}}_{{\textbf{C}}}-(\beta +\zeta _{{\textbf{C}}}+\varphi _{3}){{\textbf{I}}}_{{\textbf{C}}}={\widehat{\Phi }}_{3},\\ \dot{{\textbf{R}}}=\varphi _{2}{{\textbf{E}}}_{{\textbf{C}}}+\varphi _{3}{{\textbf{I}}}_{{\textbf{C}}}-\beta {{\textbf{R}}}={\widehat{\Phi }}_{4}. \end{array}\right. } \end{aligned}$$

The basic reproduction number ${\mathbb {R}}_{0}^{{\mathbb {C}}}$ of the model (21) is presented as

21 $$\begin{aligned} {\mathbb {R}}_{0}^{{\mathbb {C}}}=\frac{\alpha _{2}(\beta +\zeta _{{\textbf{C}}}+\varphi _{3}+\varphi _{1})}{(\beta +\varphi _{1}+\varphi _{2})(\beta +\zeta _{{\textbf{C}}}+\varphi _{3})}. \end{aligned}$$

### Sensitivity analysis

The sensitivity analysis of the model parameters for the COVID-19 submodel, as stated in (21), is carried out in this subsection. The sensitivity of a parameter, $\varepsilon$ contemplate, is expressed as^46^ and indicates how the framework behaves in response to a slight variation in a parameter value as

$$\begin{aligned} {\textbf{S}}_{\varepsilon }=\frac{\partial {\mathbb {R}}_{0}^{{\mathbb {C}}}}{\partial \varepsilon }\frac{\varepsilon }{{\mathbb {R}}_{0}^{{\mathbb {C}}}}. \end{aligned}$$

In our case, the sensitivity analysis of each parameters for (21) becomes:

22 $$\begin{aligned} {\textbf{S}}_{\alpha _{2}}{} & {} =\frac{\partial {\mathbb {R}}_{0}^{{\mathbb {C}}}}{\partial \alpha _{2}}\frac{\alpha _{2}}{{\mathbb {R}}_{0}^{{\mathbb {C}}}}=1,\nonumber \\{\textbf{S}}_{\beta }{} & {} =\frac{\partial {\mathbb {R}}_{0}^{{\mathbb {C}}}}{\partial \beta }\frac{\beta }{{\mathbb {R}}_{0}^{{\mathbb {C}}}}=-\frac{\beta \big (\zeta _{{\textbf{C}}}(\zeta _{{\textbf{C}}}+2\beta +3\varphi _{3}+\varphi _{1})+\beta (\beta +4\varphi _{3}+2\varphi _{1})+\varphi _{3}(2\varphi _{3}+2\varphi _{2}+\varphi _{1})+\varphi _{1}(\varphi _{1}+\varphi _{2})\big )}{(\zeta _{{\textbf{C}}}+\beta +\varphi _{3})(\beta +\varphi _{2}+\varphi _{1})(\zeta _{{\textbf{C}}}+\beta +2\varphi _{3}+\varphi _{1})},\nonumber \\ {\textbf{S}}_{\zeta _{{\textbf{C}}}}{} & {} =\frac{\partial {\mathbb {R}}_{0}^{{\mathbb {C}}}}{\partial \zeta _{{\textbf{C}}}}\frac{\zeta _{{\textbf{C}}}}{{\mathbb {R}}_{0}^{{\mathbb {C}}}}=-\frac{\zeta _{{\textbf{C}}}\varphi _{1}}{(\zeta _{{\textbf{C}}}+\beta +\varphi _{3})(\zeta _{{\textbf{C}}}+\beta +\varphi _{3}+\varphi _{1})},\nonumber \\ {\textbf{S}}_{\varphi _{3}}{} & {} =-\frac{\partial {\mathbb {R}}_{0}^{{\mathbb {C}}}}{\partial \varphi _{3}}\frac{\varphi _{3}}{{\mathbb {R}}_{0}^{{\mathbb {C}}}}=-\frac{\varphi _{3}\varphi _{1}}{(\zeta _{{\textbf{C}}}+\beta +\varphi _{3})(\zeta _{{\textbf{C}}}+\beta +\varphi _{3}+\varphi _{1})},\nonumber \\ {\textbf{S}}_{\varphi _{1}}{} & {} =-\frac{\partial {\mathbb {R}}_{0}^{{\mathbb {C}}}}{\partial \varphi _{1}}\frac{\varphi _{1}}{{\mathbb {R}}_{0}^{{\mathbb {C}}}}=-\frac{\varphi _{1}(\zeta _{{\textbf{C}}}+\varphi _{3}-\varphi _{2})}{(\varphi _{2}+\beta +\varphi _{3})(\zeta _{{\textbf{C}}}+\beta +\varphi _{3}+\varphi _{1})},\nonumber \\ {\textbf{S}}_{\varphi _{2}}{} & {} =-\frac{\partial {\mathbb {R}}_{0}^{{\mathbb {C}}}}{\partial \varphi _{2}}\frac{\varphi _{2}}{{\mathbb {R}}_{0}^{{\mathbb {C}}}}=-\frac{\varphi _{2}}{(\varphi _{2}+\beta +\varphi _{3})}. \end{aligned}$$

Here, we notice that the dissemination of COVID-19 is boosted by the contact rate $\alpha _{1}$. Additionally, the transmission rate $\varphi _{1}$ from the unprotected group to the afflicted group has a positive effect on the dissemination of the virus if $\zeta _{{\mathbb {C}}}+\varphi _{3}-\varphi _{2}<0.$ In other words, the prevalence will rise as the values of these factors rise. The additional parameters $\beta ,\varphi _{3},\zeta _{{\mathbb {C}}}$ and $\varphi _{2}$ have adverse effects; therefore, raising their values will result in a drop in the frequency of COVID-19 infections. Nonetheless, the sensitivity analysis investigation does not take into account the immoral increase in the individual fatality rate as a means of controlling the spread of illness.

### Bifurcation analysis

In what follows, we investigate the solution behavior of (21) by taking $\alpha _{2}$ as the bifurcation parameter. Calculating the value of $\alpha _{2}$ from ${\mathbb {R}}_{0}^{{\mathbb {C}}}$, i.e, $\frac{\alpha _{2}(\beta +\varphi _{1}+\varphi _{2})}{(\beta +\zeta _{{\mathbb {C}}}+\varphi _{3}+\varphi _{1})}\Big (\frac{(\beta +\zeta _{{\mathbb {C}}}+\varphi _{1}+\varphi _{2})}{(\beta +\zeta _{{\mathbb {C}}}+\varphi _{3})}\Big )=1,$ we have $\alpha _{2}^{*}=\frac{(\beta +\varphi _{1}+\varphi _{2})(\beta +\zeta _{{\mathbb {C}}}+\varphi _{3})}{\beta +\zeta _{{\mathbb {C}}}+\varphi _{3}+\varphi _{1}}.$ By replacing $\alpha _{2}^{*}$, we can determine the eigenvalues of the Jacobin matrix at the DFE point, as per the outcome provided in Ref.^44^. Thus, substituting $\alpha _{2}=\alpha _{2}^{*}$ in (7), it gives zero eigenvalue. This means that the Jacobean matrix ${\mathcal {J}}_{{\mathcal {E}}_{0}}$ in (7) at $\alpha _{2}=\alpha _{2}^{*}$ has a left eigenvector (associated with the zero eigenvalue) which is calculated from $o^{{\mathbb {T}}}{\mathcal {J}}_{{\mathcal {E}}_{0}}$. Here, $o=\big [o_{1},o_{2},o_{3},o_{4}\big ]$, for which $o_{1}=0,~o_{2}=\frac{\zeta _{{\mathbb {C}}}+\beta +\varphi _{1}+\varphi _{3}}{\beta +\varphi _{2}+\varphi _{1}},~~o_{3}=1$ and $o_{4}=0.$

Likewise, $e^{{\mathbb {T}}}{\mathcal {J}}_{{\mathcal {E}}_{0}}=0$ can be used to determine the right eigenvector linked to the zero eigenvalue when $e=\big [e_{1},e_{2},e_{3},e_{4}\big ],$ for which $e_{1}=\frac{\beta (\zeta _{{\mathbb {C}}}+\varphi _{2}+\beta )+(\varphi _{2}+\varphi _{1})(\zeta _{{\mathbb {C}}}+\beta +\varphi _{3})}{\varphi _{2}(\zeta _{{\mathbb {C}}}+\beta +\varphi _{3})+\varphi _{1}\varphi _{3}},~e_{2}=\frac{\beta (\zeta _{{\mathbb {C}}}+\varphi _{2}+\beta )}{\varphi _{2}(\zeta _{{\mathbb {C}}}+\beta +\varphi _{3})+\varphi _{1}\varphi _{3}},~e_{3}=\frac{\beta \varphi _{2}}{\varphi _{2}(\zeta _{{\mathbb {C}}}+\beta +\varphi _{3})+\varphi _{1}\varphi _{3}}$ and $e_{4}=1.$

Now, suppose that ${\widehat{\Phi }}_{\ell }$ represents the right-hand side of the $\ell \text{th}$ equation in the COVID-19 submodel (21) and let $\varkappa _{\ell }$ denote the corresponding state variable for $\ell =1,...,4$.

Introduce

23 $$\begin{aligned} y_{1}=\sum \limits _{\ell ,\iota ,\jmath }^{n}o_{\ell }\omega _{\iota }\omega _{\jmath }\frac{\partial ^{2}{\widehat{\Phi }}_{\ell }}{\partial \varkappa _{\iota }\partial \varkappa _{\jmath }}(0,0)~~~~~~and~~~~~y_{2}=\sum \limits _{\ell ,\iota }^{n}o_{\ell }\omega _{\iota }\frac{\partial ^{2}{\widehat{\Phi }}_{\ell }}{\partial \varkappa _{\iota }\partial \varkappa _{1}}(0,0). \end{aligned}$$

The local dynamics of (21) near the bifurcation point $\alpha _{2}=\alpha ^{*}$ are then calculated by the signs of two associated constants $y_{1}$ and$y_{2}$ with $\varkappa _{1}=\alpha _{2}-\alpha ^{*}$. Note that, in ${\widehat{\Phi }}_{\ell }(0,0)$, the first zero corresponds to the DFE, ${\mathcal {E}}_{0}^{{\mathbb {C}}}$, for (21). In other words, ${\widehat{\Phi }}_{\ell }(0,\varkappa _{1}),$ for $\ell =1,...,4$ if and only if the right-hand sides of (21) are equal to zero at ${\mathcal {E}}_{0}^{{\mathbb {C}}}.$

Moreover, from $\varkappa _{1}=\alpha _{2}-\alpha ^{*}$, we have $\varkappa _{1}=0$ when $\alpha _{2}=\alpha ^{*}$, which is the second zero component in ${\widehat{\Phi }}_{\ell }(0,0)$. For the (21), the associated nonzero partial derivatives at the ${\mathcal {E}}_{0}^{{\mathbb {C}}}$ are

24 $$\begin{aligned}{} & {} \frac{\partial ^{2}{\widehat{\Phi }}_{1}}{\partial {\textbf{E}}_{{\textbf{C}}}^{2}}=-\frac{2\alpha _{2}^{*}\beta }{\nabla },~~~\frac{\partial ^{2}{\widehat{\Phi }}_{1}}{\partial {\textbf{E}}_{{\textbf{C}}}{\textbf{I}}_{{\textbf{C}}}}=-\frac{2\alpha _{2}^{*}\beta }{\nabla },~~~\frac{\partial ^{2}{\widehat{\Phi }}_{1}}{\partial {\textbf{E}}_{{\textbf{C}}}{\textbf{R}}}=\frac{2\alpha _{2}^{*}\beta }{\nabla },\nonumber \\ {}{} & {} \frac{\partial ^{2}{\widehat{\Phi }}_{1}}{\partial {\textbf{I}}_{{\textbf{C}}}^{2}}=-\frac{2\alpha _{2}^{*}\beta }{\nabla },~~~\frac{\partial ^{2}{\widehat{\Phi }}_{1}}{\partial {\textbf{E}}_{{\textbf{C}}}{\textbf{I}}_{{\textbf{C}}}}=-\frac{2\alpha _{2}^{*}\beta }{\nabla },~~~\frac{\partial ^{2}{\widehat{\Phi }}_{1}}{\partial {\textbf{I}}_{{\textbf{C}}}{\textbf{R}}}=\frac{2\alpha _{2}^{*}\beta }{\nabla },\nonumber \\ {}{} & {} \frac{\partial ^{2}{\widehat{\Phi }}_{2}}{\partial {\textbf{E}}_{{\textbf{C}}}^{2}}=\frac{2\alpha _{2}^{*}\beta }{\nabla },~~~\frac{\partial ^{2}{\widehat{\Phi }}_{2}}{\partial {\textbf{E}}_{{\textbf{C}}}{\textbf{I}}_{{\textbf{C}}}}=\frac{2\alpha _{2}^{*}\beta }{\nabla },~~~\frac{\partial ^{2}{\widehat{\Phi }}_{2}}{\partial {\textbf{I}}_{{\textbf{C}}}{\textbf{R}}}=\frac{-2\alpha _{2}^{*}\beta }{\nabla },\nonumber \\ {}{} & {} \frac{\partial ^{2}{\widehat{\Phi }}_{2}}{\partial {\textbf{I}}_{{\textbf{C}}}^{2}}=\frac{2\alpha _{2}^{*}\beta }{\nabla },~~~\frac{\partial ^{2}{\widehat{\Phi }}_{2}}{\partial {\textbf{I}}_{{\textbf{C}}}{\textbf{E}}_{{\textbf{C}}}}=\frac{2\alpha _{2}^{*}\beta }{\nabla },~~~\frac{\partial ^{2}{\widehat{\Phi }}_{2}}{\partial {\textbf{I}}_{{\textbf{C}}}{\textbf{R}}}=\frac{-2\alpha _{2}^{*}\beta }{\nabla }. \end{aligned}$$

Next, with the aforementioned expressions for $y_{1}$, it is evident that

25 $$\begin{aligned} y_{1}=\frac{\beta ^{2}(\beta +\varphi _{3}+\zeta _{{\mathbb {C}}})(\beta +\varphi _{3}+\zeta _{{\mathbb {C}}}+\varphi _{1}+\varphi _{3})}{\nabla \big (\varphi _{3}\varphi _{1}+\varphi _{1}(\beta +\varphi _{3}+\zeta _{{\mathbb {C}}})\big )}\Bigg \{\frac{2\beta (\beta +\varphi _{3}+\varphi _{1}+\zeta _{{\mathbb {C}}})}{\varphi _{2}(\beta +\varphi _{3}+\zeta _{{\mathbb {C}}})+\varphi _{1}\varphi _{3}}-1\Bigg \}. \end{aligned}$$

It can be demonstrated that the corresponding non-vanishing partial derivatives for the corresponding sign of $y_{2}$ are

26 $$\begin{aligned} \frac{\partial ^{2}{\widetilde{\Phi }}_{1}}{\partial {\textbf{E}}_{{\textbf{C}}}\partial \alpha _{2}}=-1,~\frac{\partial ^{2}{\widetilde{\Phi }}_{1}}{\partial {\textbf{I}}_{{\textbf{C}}}\partial \alpha _{2}}=-1,~\frac{\partial ^{2}{\widetilde{\Phi }}_{2}}{\partial {\textbf{E}}_{{\textbf{C}}}\partial \alpha _{2}}=1,~\frac{\partial ^{2}{\widetilde{\Phi }}_{2}}{\partial {\textbf{E}}_{{\textbf{C}}}\partial \alpha _{2}}=1. \end{aligned}$$

It is evident from the aforementioned statements as well that $y_{2}=\frac{\beta (\zeta _{{\mathbb {C}}+\beta +\varphi _{3}+\varphi _{1}})^{2}}{(\beta +\varphi _{2}+\varphi _{1})(\varphi _{2}(\beta +\varphi _{3}+\zeta _{{\mathbb {C}}})+\varPhi _{1}\varphi _{3})}.$

From the bifurcation coefficient $y_{1}$’s sign varies on the minimal value of recurrence that generates bistability $(\ell )$, and we find that $y_{2}$ is always positive from the estimates of $y_{1}$ and $y_{2}$. Therefore, a subsequent proposition is established by applying the result of Ref.^44^.

#### Proposition 1

The system (21) has a forward bifurcation if the minimal value $\ell =2\beta (\zeta _{{\mathbb {C}}}+\beta +\varphi _{1}+\varphi _{3})/\varphi _{2}(\beta +\varphi _{3}+\zeta _{{\mathbb {C}}}+\varphi _{1}\varphi _{3})$ of the virus infection that causes bistability is smaller than unity.

We then conclude with the following theorem

#### Theorem 6

If $${\mathbb {R}}_{0}^{{\mathbb {C}}}=1,$$ then

(i): The model (21) undergoes a backward bifurcation whenever $y_{2}>0$.
(ii): The model (21) undergoes a forward bifurcation whenever $y_{2}<0$.

**Figure 5:**
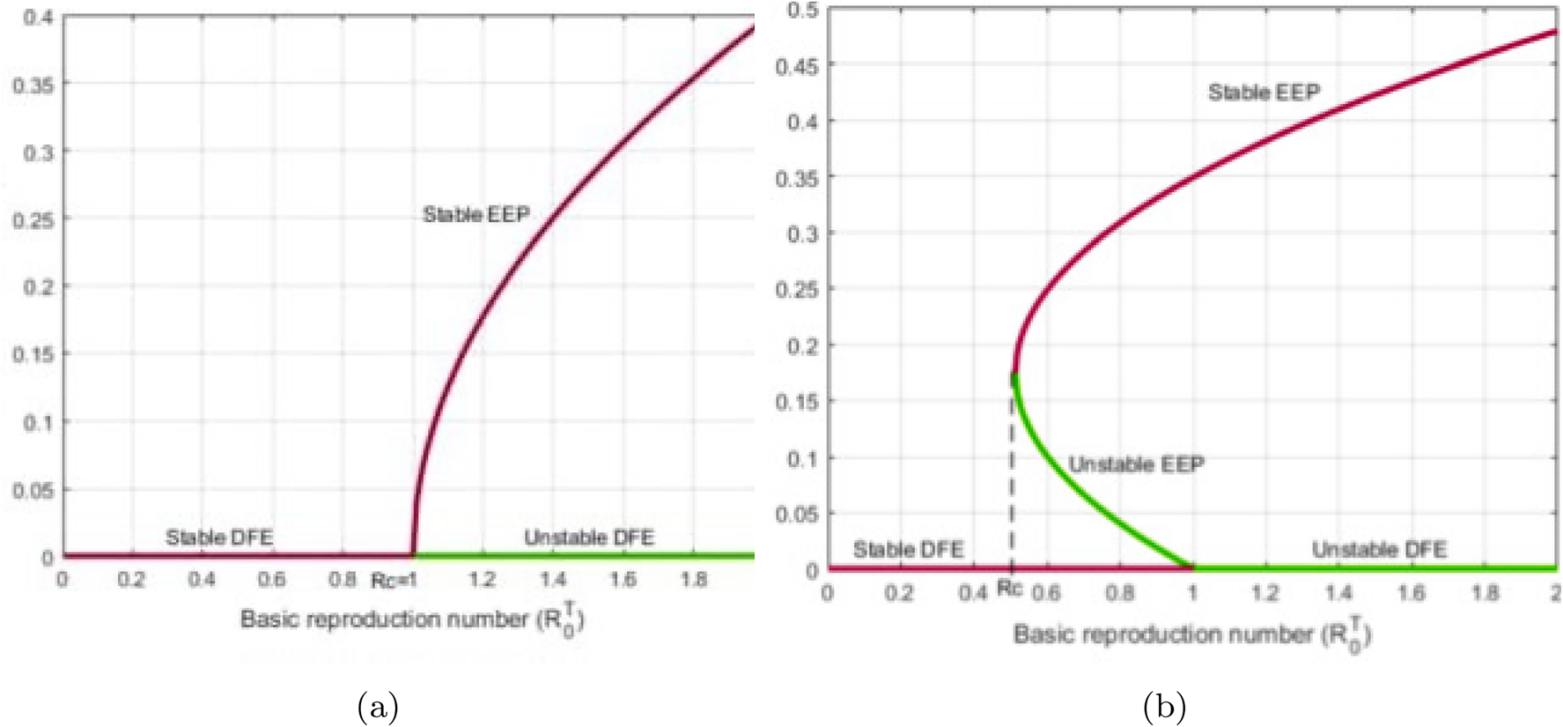
Simulation of the codynamics model (3) to illustrate the occurrence of **(a)** forward **(b)** backward bifurcation.

The bifurcation phenomenon is illustrated in Fig. 5a,b, where we have carried out a numerical simulation of the infection model (21). The system parameter values are presented in Table 2, the calculation gives $y_{2}=0.4321>0$ and $y_{1}=0.0032>0$, the backward bifurcation condition is then satisfied and we obtain Fig. 5a. Also, the forward bifurcation Fig. 5b is obtained for $\varkappa _{1}=\alpha _{2}-\alpha ^{*}.$ Here, the parameters we have used may not all be epidemiologically realistic (see^47^).

## Stochastic configuration of codynamics of TB-COVID-19 model

We examine how random interference affects the distinctiveness and presence of a stable dispersion, as well as the gradual disappearance of diseases, in the system (2). The formula that follows is a representation of the stochastic adaptation relating to the model (2) is

27 $$\begin{aligned} {\left\{ \begin{array}{ll} d{\textbf{S}}=\big [\nabla -(\psi _{{\textbf{T}}}+\psi _{{\textbf{C}}}+\beta ){\textbf{S}}\big ]d\tau +\wp _{1}{\textbf{S}}d{\mathbb {B}}_{1}(\tau ),\\ d{{\textbf{L}}}_{{\textbf{T}}}=\big [\psi _{{\textbf{T}}}{\textbf{S}}-(\beta +\mu +\lambda \psi _{{\textbf{C}}}+\varpi ){{\textbf{L}}}_{{\textbf{T}}}\big ]d\tau +\wp _{2}{{\textbf{L}}}_{{\textbf{T}}}d{\mathbb {B}}_{2}(\tau ),\\ d{{\textbf{I}}}_{{\textbf{T}}}=\big [\mu {{\textbf{L}}}_{{\textbf{T}}}+\varsigma _{2}\eta {{\textbf{L}}}_{{\textbf{T}}{\textbf{C}}}+\theta _{2}\xi {{\textbf{I}}}_{{\textbf{T}}{\textbf{C}}}-(\beta +\varsigma _{3}+\zeta _{{\textbf{T}}}+\delta ){{\textbf{I}}}_{{\textbf{T}}}\big ]d\tau +\wp _{3}{{\textbf{I}}}_{{\textbf{T}}}d{\mathbb {B}}_{3}(\tau ),\\ d{{\textbf{E}}}_{{\textbf{C}}}=\big [\psi _{{\textbf{C}}}{\textbf{S}}-(\beta +\epsilon \psi _{{\textbf{T}}}+\varphi _{1}+\varphi _{2}){{\textbf{E}}}_{{\textbf{C}}}\big ]d\tau +\wp _{4}{{\textbf{E}}}_{{\textbf{C}}}d{\mathbb {B}}_{4}(\tau ),~~~~~~~~~~~~~~~~~\intercal _{2}\le \tau \le \intercal ,\\ d{{\textbf{I}}}_{{\textbf{C}}}=\big [\varphi _{1} {{\textbf{E}}}_{{\textbf{C}}}+\rho \eta {{\textbf{L}}}_{{\textbf{T}}{\textbf{C}}}+\theta _{1}\xi {{\textbf{I}}}_{{\textbf{T}}{\textbf{C}}}-(\beta +\zeta _{{\textbf{C}}}+\nu +\varphi _{3}){{\textbf{I}}}_{{\textbf{C}}}\big ]d\tau +\wp _{5}{{\textbf{I}}}_{{\textbf{C}}}d{\mathbb {B}}_{5}(\tau ),\\ d{{\textbf{L}}}_{{\textbf{T}}{\textbf{C}}}=\big [\lambda \psi _{{\textbf{C}}}{{\textbf{L}}}_{{\textbf{T}}}+\epsilon \psi _{{\textbf{T}}}{{\textbf{E}}}_{{\textbf{C}}}-(\beta +\zeta _{{\textbf{C}}}+\rho +\eta ){{\textbf{L}}}_{{\textbf{T}}{\textbf{C}}} \big ]d\tau +\wp _{6}{{\textbf{L}}}_{{\textbf{T}}{\textbf{C}}}d{\mathbb {B}}_{6}(\tau ),\\ d{{\textbf{I}}}_{{\textbf{T}}{\textbf{C}}}=\big [\rho {{\textbf{L}}}_{{\textbf{T}}{\textbf{C}}}+\varsigma _{3}{{\textbf{I}}}_{{\textbf{T}}}+\nu {{\textbf{I}}}_{{\textbf{C}}}-(\beta +\zeta _{{\textbf{T}}{\textbf{C}}}+\xi ){{\textbf{I}}}_{{\textbf{T}}{\textbf{C}}} \big ]d\tau +\wp _{7}{{\textbf{I}}}_{{\textbf{T}}{\textbf{C}}}d{\mathbb {B}}_{7}(\tau ),\\ d{{\textbf{R}}}=\big [\varpi {{\textbf{L}}}_{{\textbf{T}}}+\varphi _{2}{{\textbf{E}}}_{{\textbf{C}}}+\delta {{\textbf{I}}}_{{\textbf{T}}}+\varphi _{3}{{\textbf{I}}}_{{\textbf{C}}}+(1-(\varsigma _{1}+\varsigma _{2}))\eta {{\textbf{L}}}_{{\textbf{T}}{\textbf{C}}}+(1-(\theta _{1}+\theta _{2}))\xi {{\textbf{I}}}_{{\textbf{T}}{\textbf{C}}}-\beta {\textbf{R}}\big ]d\tau \\ \quad \qquad +\wp _{8}{{\textbf{R}}}d{\mathbb {B}}_{8}(\tau ),\end{array}\right. } \end{aligned}$$

in which $\wp _{\jmath }$ indicate the variability in noise and ${{\mathbb {B}}_{\jmath }}(\tau ),~~(\jmath =1,...,8)$ are conventional one-dimensional autonomous Brownian movements. The additional parameters have the same relevance as they do in system (2).

In the sequel, let $(\Upsilon ,{\mathfrak {F}},\{{\mathfrak {F}}_{\tau }\}_{\tau \ge 0},{\mathbb {P}})$ be a complete probability space and its filtration $\{{\mathfrak {F}}_{\tau }\}_{\tau \ge 0}$ needs to fulfill the standard requirements (that is., it must be right continuous and comprise all ${\mathbb {P}}$-null sets), whilst ${{\mathbb {B}}}_{\jmath }(\tau ),~(\jmath =1,...,8)$ are stated on the complete probability space. In addition, take ${\mathbb {R}}_{+}=\big \{\Lambda \ge 0\big \},~{\mathbb {R}}_{+}^{8}=\big \{\Lambda =(\Lambda _{1},...,\Lambda _{8})\in {\mathbb {R}}^{8}:\Lambda _{\jmath }>0,~\jmath =1,...,8\big \}.$ For any matrix ${\mathbb {M}},$ its transpose is indicated by ${\mathbb {M}}^{\bar{T}}.$

Utilizing $\Lambda (\tau )=\big ({\textbf{S}}(\tau ),{{\textbf{L}}}_{{\textbf{T}}}(\tau ),{{\textbf{I}}}_{{\textbf{T}}}(\tau ),{{\textbf{E}}}_{{\textbf{C}}}(\tau ),{{\textbf{I}}}_{{\textbf{C}}}(\tau ),{{\textbf{L}}}_{{\textbf{T}}{\textbf{C}}}(\tau ),{{\textbf{I}}}_{{\textbf{T}}{\textbf{C}}}(\tau ),{\textbf{R}}(\tau )\big )^{\bar{T}}$ as the solution of model (28) supplemented by ICs $\Lambda (0)=\big ({\textbf{S}}(0),{{\textbf{L}}}_{{\textbf{T}}}(0),{{\textbf{I}}}_{{\textbf{T}}}(0),{{\textbf{E}}}_{{\textbf{C}}}(0),{{\textbf{I}}}_{{\textbf{C}}}(0),{{\textbf{L}}}_{{\textbf{T}}{\textbf{C}}}(0),{{\textbf{I}}}_{{\textbf{T}}{\textbf{C}}}(0),{\textbf{R}}(0)\big )^{\bar{T}}.$ Furthermore, we utilize ${{\textbf{z}}}_{1}\vee ..\vee {{\textbf{z}}}_{\kappa }$ to represent $\max \{{{\textbf{z}}}_{1}...{{\textbf{z}}}_{\kappa }\}$ and ${{\textbf{z}}}\wedge ...\wedge {{\textbf{z}}}_{\kappa }$ to show $\min \{{{\textbf{z}}}_{1}...{{\textbf{z}}}_{\kappa }\}.$

Firstly, we assert an outcome about the existence–uniqueness of a global non-negative solution for system (28).

### Theorem 7

Assume that there is a unique solution $\Lambda (\tau )\in {\mathbb {R}}_{+}^{8}$ of structure (28) on $[0,\infty )$ for any starting value $\Lambda (0)\in {\mathbb {R}}_{+}^{8}.$ It stays in $\in {\mathbb {R}}_{+}^{8}$ having probability 1 (a.s).

### Proof

Here, we overlook the initial portion of the explanation just to display the essential Lyapunov function because it is comparable to Theorem 2.1 in^34^.

Introducing a ${\mathbb {C}}^{2}$-functional $\Phi _{0}$ on ${\mathbb {R}}_{+}^{8}\mapsto {\mathbb {R}}_{+}$ by

28 $$\begin{aligned} \Phi _{0}(\Lambda ){} & {} =\Big [\big ({{\textbf{S}}}-\ell -\ell \ln \frac{{\textbf{S}}}{\ell }\Big )+({{\textbf{L}}}_{{\textbf{T}}}-1-\ln {{\textbf{L}}}_{{\textbf{T}}})+({{\textbf{I}}}_{{\textbf{T}}}-1-\ln {{\textbf{I}}}_{{\textbf{T}}})+({{\textbf{E}}}_{{\textbf{C}}}-1-\ln {{\textbf{E}}}_{{\textbf{C}}})\nonumber \\ {}{} & {} \quad +({{\textbf{I}}}_{{\textbf{C}}}-1-\ln {{\textbf{I}}}_{{\textbf{C}}})+({{\textbf{L}}}_{{\textbf{T}}{\textbf{C}}}-1-\ln {{\textbf{L}}}_{{\textbf{T}}{\textbf{C}}})+({{\textbf{I}}}_{{\textbf{T}}{\textbf{C}}}-1-\ln {{\textbf{I}}}_{{\textbf{T}}{\textbf{C}}})+({{\textbf{R}}}-1-\ln {{\textbf{R}}})\Big ],\nonumber \\ \end{aligned}$$

where the value of the non-negative constant $\ell$ will be obtained hereafter. When we implement Itô’s algorithm^48^ to $\Phi _{0}$, we obtain

29 $$\begin{aligned} d\Phi _{0}(\Lambda ){} & {} ={\mathcal {L}}\Phi _{0}(\Lambda )d\tau +\wp _{1}({\textbf{S}}-\ell )d{\mathbb {B}}_{1}(\tau )+\wp _{2}({{\textbf{L}}}_{{\textbf{T}}}-1)d{\mathbb {B}}_{2}(\tau )+\wp _{3}({{\textbf{I}}}_{{\textbf{T}}}-1)d{\mathbb {B}}_{3}(\tau )\nonumber \\ {}{} & {} \quad +\wp _{4}({{\textbf{E}}}_{{\textbf{C}}}-1)d{\mathbb {B}}_{4}(\tau )+\wp _{5}({{\textbf{I}}}_{{\textbf{C}}}-1)d{\mathbb {B}}_{5}(\tau )+\wp _{6}({{\textbf{L}}}_{{\textbf{T}}{\textbf{C}}}-1)d{\mathbb {B}}_{6}(\tau )\nonumber \\ {}{} & {} \quad +\wp _{7}({{\textbf{I}}}_{{\textbf{T}}{\textbf{C}}}-1)d{\mathbb {B}}_{7}(\tau )+\wp _{8}({{\textbf{R}}}-1)d{\mathbb {B}}_{8}(\tau ), \end{aligned}$$

where ${\mathcal {L}}\Phi _{0}:{\mathbb {R}}_{+}^{8}\mapsto {\mathbb {R}}$ is determined by

30 $$\begin{aligned} {\mathcal {L}}\Phi _{0}(\Lambda ){} & {} =\Big (1-\frac{\ell }{{\textbf{S}}}\Big )\Big [\nabla -(\psi _{{\textbf{T}}}+\psi _{{\textbf{C}}}+\beta ){\textbf{S}}\Big ]+\frac{\ell }{2}\wp _{1}^{2}\nonumber \\ {}{} & {} \quad +\Big (1-\frac{1}{{{\textbf{L}}}_{{\textbf{T}}}}\Big )\Big [\psi _{{\textbf{T}}}{\textbf{S}}-(\beta +\mu +\lambda \psi _{{\textbf{C}}}+\varpi ){{\textbf{L}}}_{{\textbf{T}}}\Big ]+\frac{1}{2}\wp _{2}^{2}\nonumber \\ {}{} & {} \quad + \Big (1-\frac{1}{{{\textbf{I}}}_{{\textbf{T}}}}\Big )\Big [\mu {{\textbf{L}}}_{{\textbf{T}}}+\varsigma _{2}\eta {{\textbf{L}}}_{{\textbf{T}}{\textbf{C}}}+\theta _{2}\xi {{\textbf{I}}}_{{\textbf{T}}{\textbf{C}}}-(\beta +\varsigma _{3}+\zeta _{{\textbf{T}}}+\delta ){{\textbf{I}}}_{{\textbf{T}}}\Big ]+\frac{1}{2}\wp _{3}^{2}\nonumber \\ {}{} & {} \quad + \Big (1-\frac{1}{{{\textbf{E}}}_{{\textbf{C}}}}\Big )\Big [\psi _{{\textbf{C}}}{{\textbf{S}}}-(\beta +\epsilon \psi _{{\textbf{T}}}+\varphi _{1}+\varphi _{2}){{\textbf{E}}}_{{\textbf{C}}}\Big ]+\frac{1}{2}\wp _{4}^{2}\nonumber \\ {}{} & {} \quad + \Big (1-\frac{1}{{{\textbf{I}}}_{{\textbf{C}}}}\Big )\Big [\varphi _{1}{{\textbf{E}}}_{{\textbf{C}}}+\rho \eta {{\textbf{L}}}_{{\textbf{T}}{\textbf{C}}}+\theta _{1}\xi {{\textbf{I}}}_{{\textbf{T}}{\textbf{C}}}-(\beta +\zeta _{{\textbf{C}}}+\nu +\varphi _{3}){{\textbf{I}}}_{{\textbf{C}}}\Big ]+\frac{1}{2}\wp _{5}^{2}\nonumber \\ {}{} & {} \quad + \Big (1-\frac{1}{{{\textbf{L}}}_{{\textbf{T}}{\textbf{C}}}}\Big )\Big [\lambda \psi _{{\textbf{C}}}{{\textbf{L}}}_{{\textbf{T}}}+\epsilon \psi _{{\textbf{T}}}{{\textbf{E}}}_{{\textbf{C}}}-(\beta +\zeta _{{\textbf{C}}}+\rho +\eta ){{\textbf{L}}}_{{\textbf{T}}{\textbf{C}}}\Big ]+\frac{1}{2}\wp _{6}^{2}\nonumber \\ {}{} & {} \quad + \Big (1-\frac{1}{{{\textbf{I}}}_{{\textbf{T}}{\textbf{C}}}}\Big )\Big [\rho {{\textbf{L}}}_{{\textbf{T}}{\textbf{C}}}+\varsigma _{3}{{\textbf{I}}}_{{\textbf{T}}}+\nu {{\textbf{I}}}_{{\textbf{C}}}-(\beta +\zeta _{{\textbf{T}}{\textbf{C}}}+\xi ){{\textbf{I}}}_{{\textbf{T}}{\textbf{C}}}\Big ]+\frac{1}{2}\wp _{7}^{2}\nonumber \\ {}{} & {} \quad + \Big (1-\frac{1}{{{\textbf{R}}}}\Big )\Big [\varpi {{\textbf{L}}}_{{\textbf{T}}}+\varphi _{2}{{\textbf{E}}}_{{\textbf{C}}}+\delta {{\textbf{I}}}_{{\textbf{T}}}+\varphi _{3}{{\textbf{I}}}_{{\textbf{C}}}+(1-(\varsigma _{1}+\varsigma _{2}))\eta {{\textbf{I}}}_{{\textbf{T}}{\textbf{C}}}+(1-(\theta _{1}+\theta _{2}))\xi {{\textbf{L}}}_{{\textbf{T}}{\textbf{C}}}-\beta {\textbf{R}}\Big ]+\frac{1}{2}\wp _{8}^{2}\nonumber \\ {}{} & {} \le \big (\nabla -\ell (\psi _{{\textbf{T}}}+\psi _{{\textbf{C}}}+\beta )\big )+(\beta +\mu +\lambda \psi _{{\textbf{C}}}+\varpi )+(\beta +\varsigma _{3}+\zeta _{{\textbf{T}}}+\delta )+(\beta +\epsilon \psi _{{\textbf{T}}}+\varphi _{1}+\varphi _{2})\nonumber \\ {}{} & {} \quad +(\beta +\zeta _{{\textbf{C}}}+\nu +\varphi _{3})+(\beta +\zeta _{{\textbf{C}}}+\rho +\eta )+(\beta +\zeta _{{\textbf{T}}{\textbf{C}}}+\xi )+\beta \nonumber \\ {}{} & {} \quad +\frac{1}{2}\big (\ell \wp _{1}^{2}+\wp _{2}^{2}+\wp _{3}^{2}+\wp _{4}^{2}+\wp _{5}^{2}+\wp _{6}^{2}+\wp _{7}^{2}+\wp _{8}^{2}\big ). \end{aligned}$$

Letting $\ell =\nabla /\psi _{{\textbf{T}}}+\psi _{{\textbf{C}}}+\beta .$ As a result, we have

31 $$\begin{aligned} {\mathcal {L}}\Phi _{0}(\Lambda ){} & {} \le (\beta +\mu +\lambda \psi _{{\textbf{C}}}+\varpi )+(\beta +\varsigma _{3}+\zeta _{{\textbf{T}}}+\delta )+(\beta +\epsilon \psi _{{\textbf{T}}}+\varphi _{1}+\varphi _{2})\nonumber \\ {}{} & {} \quad +(\beta +\zeta _{{\textbf{C}}}+\nu +\varphi _{3})+(\beta +\zeta _{{\textbf{C}}}+\rho +\eta )+(\beta +\zeta _{{\textbf{T}}{\textbf{C}}}+\xi )+\beta \nonumber \\ {}{} & {} \quad +\frac{1}{2}\big (\frac{\nabla }{\psi _{{\textbf{T}}}+\psi _{{\textbf{C}}}+\beta }\wp _{1}^{2}+\wp _{2}^{2}+\wp _{3}^{2}+\wp _{4}^{2}+\wp _{5}^{2}+\wp _{6}^{2}+\wp _{7}^{2}+\wp _{8}^{2}\big )\nonumber \\ {}{} & {} :={\mathcal {K}}, \end{aligned}$$

where the constant ${\mathcal {K}}$ is non-negative. According to Ref.^34^, we similarly exclude the remaining portion of the explanation. The documentation is now complete. $\square$

### Stationary distribution

Our primary concern with the stochastic outbreak framework is the virus’s permanence. In this portion, we employ a novel method to demonstrate that structure (28) has a unique ESD, depending on the hypothesis of Khasminskii^49^.

By developing appropriate Lyapunov functions, we will show adequate conditions for the development of a unique ESD. A key component of our major result’s explanation is the lemma that follows.

Assume that ${\mathcal {Y}}(\tau )$ is an ordinary time-homogeneous Markov phenomenon with ${\mathbb {R}}^{{\mathbb {S}}}$. Its stochastic DE is as follows:

32 $$\begin{aligned} d{\mathcal {Y}}(\tau )={\textbf{b}}({\textbf{y}})(\tau )+\sum \limits _{{\textbf{w}}=1}^{{\textbf{s}}}\eta _{{\textbf{w}}}({\mathcal {Y}})d{{\mathbb {B}}}_{{\textbf{w}}}(\tau ), \end{aligned}$$

and the diffusion matrix is $A_{1}(\varkappa )=\big ({a}_{\jmath \kappa }(\varkappa )\big )_{\jmath \ge 1, {\textbf{s}}\ge \kappa },~{a}_{\jmath \kappa }(\varkappa )=\sum \limits _{{\textbf{w}}=1}^{l}\eta _{{\textbf{w}}}^{\jmath }(\varkappa )\eta _{{\textbf{w}}}^{\kappa }(\varkappa ).$ Consider the differential operator L connected to (36) as follows:

33 $$\begin{aligned} {\mathcal {L}}=\sum \limits _{\jmath =1}^{{\textbf{s}}}{{\textbf{b}}}_{\jmath }(\varkappa )\frac{\partial }{\partial {\varkappa }_{\jmath }}+\frac{1}{2}\sum \limits _{\jmath ,\kappa =1}^{{\textbf{s}}}{\textbf{Q}}_{\jmath \kappa }(\varkappa )\frac{\partial ^{2}}{\partial {\varkappa }_{\jmath }\partial {\varkappa }_{\kappa }}. \end{aligned}$$

#### Lemma 1

(^49^) Let us suppose the subsequent characteristics of a bounded open region ${\mathcal {D}}_{\epsilon }\in {\mathbb {R}}^{{\mathbb {S}}}$ with a regular boundary:

(${\mathcal {{\textbf {H}}}}_{{\textbf {1}}}$):  In the region ${\mathcal {D}}_{\epsilon }\in {\mathbb {R}}^{{\mathbb {S}}}$ and some neighborhood therefore, the least significant eigenvalue of the diffusion matrix ${\textbf{Q}}(\varkappa )$ is bounded away from zero.

(${\mathcal {{\textbf {H}}}}_{{\textbf {2}}}$): $\exists$ a positive ${\mathbb {C}}^{2}$-function $\Phi$ so that ${\mathcal {L}}\Phi$ is negative for all ${\mathbb {R}}^{{\mathbb {S}}}\setminus {\mathcal {D}}_{\epsilon }.$

Then the Markov procedure ${\mathcal {Y}}(\tau )$ has a stationary distribution $\pi (.\,).$ Also, consider ${\mathcal {F}}(\varkappa )$ be a mapping which is positive in regard to the measure $\pi ,~~\forall ~\varkappa \in {\mathbb {R}}^{{\mathbb {S}}},$ ones obtain

34 $$\begin{aligned} {\mathbb {P}}\Bigg \{\lim _{{\textbf{T}}\mapsto \infty }\frac{1}{{\textbf{T}}}\int \limits _{0}^{{\textbf{T}}}{\mathcal {F}}\big ({\mathcal {Y}}(\tau )\big )d\tau =\int \limits _{{\mathbb {R}}^{{\mathbb {S}}}}{\mathcal {F}}(\varkappa )\varphi _{2}(d\varkappa )\Bigg \}=1. \end{aligned}$$

To begin with, we establish a few concepts for ease of use in later explanations. By resolving the subsequent (36) as

35 $$\begin{aligned} {\left\{ \begin{array}{ll} \nabla =\Big (\psi _{{\textbf{T}}}+\psi _{{\textbf{C}}}+\beta +\frac{\wp _{1}^{2}}{2}\Big )\tilde{{\textbf{S}}},\\ \psi _{{\textbf{T}}}\tilde{{\textbf{S}}}=\Big (\beta +\mu +\lambda \psi _{{\textbf{C}}}+\varpi +\frac{\wp _{2}^{2}}{2}\Big )\tilde{{{\textbf{L}}}_{{\textbf{T}}}},\\ \lambda \psi _{{\textbf{C}}}\tilde{{{\textbf{L}}}_{{\textbf{T}}}}=\Big (\beta +\zeta _{{\textbf{C}}}+\rho +\eta +\frac{\wp _{6}^{2}}{2}\Big )\tilde{{{\textbf{L}}}_{{\textbf{T}}{\textbf{C}}}},\\ \varpi \tilde{{{\textbf{L}}}_{{\textbf{T}}}}+(1-(\varsigma _{1}+\varsigma _{2}))\eta \tilde{{{\textbf{L}}}_{{\textbf{T}}{\textbf{C}}}}=\big (\beta +\frac{\wp _{8}^{2}}{2}\big ) {\textbf{R}},\end{array}\right. } \end{aligned}$$

we find

36 $$\begin{aligned} \tilde{{\textbf{S}}}{} & {} =\frac{\nabla }{\psi _{{\textbf{T}}}+\psi _{{\textbf{C}}}+\beta +\frac{\wp _{1}^{2}}{2}},\nonumber \\\tilde{{{\textbf{L}}}_{{\textbf{T}}}}{} & {} =\frac{\psi _{{\textbf{T}}}\nabla }{\bigg (\psi _{{\textbf{T}}}+\psi _{{\textbf{C}}}+\beta +\frac{\wp _{1}^{2}}{2}\bigg )\bigg (\beta +\mu +\lambda \psi _{{\textbf{C}}}+\varpi +\frac{\wp _{2}^{2}}{2}\bigg )},\nonumber \\\tilde{{{\textbf{L}}}_{{\textbf{T}}{\textbf{C}}}}{} & {} =\frac{\lambda \psi _{{\textbf{C}}}}{\bigg (\psi _{{\textbf{T}}}+\psi _{{\textbf{C}}}+\beta +\frac{\wp _{1}^{2}}{2}\bigg )\bigg (\beta +\mu +\lambda \psi _{{\textbf{C}}}+\varpi +\frac{\wp _{2}^{2}}{2}\bigg )\bigg (\beta +\zeta _{{\textbf{C}}}+\rho +\eta +\frac{\wp _{6}^{2}}{2}\bigg )},\nonumber \\ \tilde{{\textbf{R}}}{} & {} =\frac{\varpi \psi _{{\textbf{T}}}\nabla \bigg (\beta +\zeta _{{\textbf{C}}}+\rho +\eta +\frac{\wp _{6}^{2}}{2}\bigg )+\bigg (1-(\varsigma _{1}+\varsigma _{2})\eta \bigg )\lambda \psi _{{\textbf{C}}}}{\bigg (\psi _{{\textbf{T}}}+\psi _{{\textbf{C}}}+\beta +\frac{\wp _{1}^{2}}{2}\bigg )\bigg (\beta +\mu +\lambda \psi _{{\textbf{C}}}+\varpi +\frac{\wp _{2}^{2}}{2}\bigg )\bigg (\beta +\zeta _{{\textbf{C}}}+\rho +\eta +\frac{\wp _{6}^{2}}{2}\bigg )\bigg (\beta +\frac{\wp _{8}^{2}}{2}\bigg )}. \end{aligned}$$

Afterwards, by addressing the subsequent formula’s:

37 $$\begin{aligned} {\left\{ \begin{array}{ll} \theta _{2}\xi \tilde{{{\textbf{I}}}_{{\textbf{T}}{\textbf{C}}}}=\Big (\beta +\varsigma _{3}+\zeta _{{\textbf{T}}}+\delta +\frac{\wp _{3}^{2}}{2}\Big )\tilde{{{\textbf{I}}}_{{\textbf{T}}}},\\ \tilde{{{\textbf{E}}}_{{\textbf{C}}}}=1,\\ \varphi _{1} \tilde{{{\textbf{E}}}_{{\textbf{C}}}}+\theta _{1}\xi \tilde{{{\textbf{I}}}_{{\textbf{T}}{\textbf{C}}}}=\Big (\beta +\zeta _{{\textbf{C}}}+\nu +\varphi _{3}+\frac{\wp _{5}^{2}}{2}\Big )\tilde{{{\textbf{I}}}_{{\textbf{C}}}},\\ \varsigma _{3}\tilde{{{\textbf{I}}}_{{\textbf{T}}}}+\nu \tilde{{{\textbf{I}}}_{{\textbf{C}}}}=\Big (\beta +\zeta _{{\textbf{T}}{\textbf{C}}}+\xi +\frac{\wp _{7}^{2}}{2}\Big ), \end{array}\right. } \end{aligned}$$

which lead us

38 $$\begin{aligned} {\left\{ \begin{array}{ll} \tilde{{{\textbf{I}}}_{{\textbf{T}}}}=\frac{\theta _{2}\bigg (\varphi _{1} \bigg (G_{1}G_{3}(G_{1}G_{2}-\theta _{2}^{2}\xi ^{2}\varsigma _{3})-(G_{1}G_{3}-\theta _{1}\theta _{2}\xi ^{2}\nu )(G_{1}G_{2}-\theta _{2}^{2}\xi ^{2}\varsigma _{3})+\theta _{1}\theta _{2}^{3}\xi ^{4}\nu \bigg )\bigg )}{\theta _{1}G_{2} {\mathcal {F}}_{1}},\\ \tilde{{{\textbf{E}}}_{{\textbf{C}}}}=1,\\ \tilde{{{\textbf{I}}}_{{\textbf{C}}}}=\frac{\varphi _{1} G_{1}(G_{1}G_{2}-n^{2}\xi ^{2}\varsigma _{3})}{{\mathcal {F}}_{1}},\\ \tilde{{{\textbf{I}}}_{{\textbf{T}}{\textbf{C}}}}=\frac{\varphi _{1} \bigg (G_{1}G_{3}(G_{1}G_{2}-\theta _{2}^{2}\xi ^{2}\varsigma _{3})-(G_{1}G_{3}-\theta _{1}\theta _{2}\xi ^{2}\nu )(G_{1}G_{2}-\theta _{2}^{2}\xi ^{2}\varsigma _{3})+\theta _{1}\theta _{2}^{3}\xi ^{4}\nu \bigg )}{\theta _{1}\xi {\mathcal {F}}_{1}},\\ \end{array}\right. } \end{aligned}$$

where $$G_{1}=\beta +\zeta _{{\textbf{T}}{\textbf{C}}}+\xi +\frac{\wp _{7}^{2}}{2},$$ $$G_{2}=\beta +\varsigma _{3}+\zeta _{{\textbf{T}}}+\delta +\frac{\rho _{3}^{2}}{2},$$ $$G_{3}=\beta +\zeta _{{\textbf{C}}}+\nu +\varphi _{3}+\frac{\wp _{5}^{2}}{2},$$ with

$$\begin{aligned} {\mathcal {F}}_{1}=(G_{1}G_{3}-\theta _{1}\theta _{2}\xi ^{2}\nu )(G_{1}G_{2}-\theta _{2}^{2}\xi ^{2}\varsigma _{3})-\theta _{1}\theta _{2}^{3}\xi ^{4}\nu . \end{aligned}$$

Introduce

39 $$\begin{aligned} {\mathbb {R}}_{0}^{{\mathbb {S}}}=\frac{\psi _{{\textbf{C}}}\widetilde{{\textbf{S}}}}{{(\beta +\epsilon \psi _{{\textbf{T}}}+\varphi _{1}+\varphi _{2})}+\frac{\wp _{4}^{2}}{2}}.\end{aligned}$$

#### Theorem 8

If we suppose that ${\mathbb {R}}_{0}^{{\mathbb {S}}}>1$, then structure (28) permits a unique ESD, $\pi (.\,).$

#### Proof

It is necessary to validate assumptions $({\mathcal {H}}_{1})$ and $({\mathcal {H}}_{2})$ in Lemma 1 for the purpose of establishing Theorem 8.

To begin with, we create an appropriate Lyapunov function $\Phi$ and identify a closed set ${\mathcal {D}}_{\epsilon }\in {\mathbb {R}}_{+}^{8}$ that ensures $\sup \limits _{\varkappa \in {\mathbb {R}}_{+}^{8}\setminus {\mathcal {D}}_{\epsilon }}{\mathcal {L}}\Phi (\varkappa )$ is negative in order to ensure the efficacy of $({\mathcal {H}}_{2})$ in Lemma 1.

For this, let us suppose

$$\begin{aligned} \widetilde{{\textbf{S}}}=\frac{{\textbf{S}}}{\overline{{\textbf{S}}}},~~~\widetilde{{{\textbf{L}}}_{{\textbf{T}}}}=\frac{{{\textbf{L}}}_{{\textbf{T}}}}{\overline{{{\textbf{L}}}_{{\textbf{T}}}}},~~~\widetilde{{{\textbf{L}}}_{{\textbf{T}}{\textbf{C}}}}=\frac{{{\textbf{L}}}_{{\textbf{T}}{\textbf{C}}}}{\overline{{{\textbf{L}}}_{{\textbf{T}}{\textbf{C}}}}},~~~\widetilde{{{\textbf{R}}}}=\frac{{\textbf{R}}}{\overline{{{\textbf{R}}}}}. \end{aligned}$$

Implementing Itô’s technique to $-\ln {\textbf{S}},$ we find

40 $$\begin{aligned} {\mathcal {L}}(-\ln {\textbf{S}}){} & {} \le -\frac{\nabla }{{\textbf{S}}}+\big (\alpha _{1}({{\textbf{I}}}_{{\textbf{T}}}+{{\textbf{I}}}_{{\textbf{T}}{\textbf{C}}})+\alpha _{2}({{\textbf{E}}}_{{\textbf{C}}}+{{\textbf{I}}}_{{\textbf{C}}}+{{\textbf{L}}}_{{\textbf{T}}{\textbf{C}}}+{I}_{{\textbf{T}}{\textbf{C}}})+\beta \big )+\frac{\wp _{1}^{2}}{2}\nonumber \\ {}{} & {} =-\frac{\nabla }{\widetilde{{\textbf{S}}}\overline{{\textbf{S}}}}+\big (\alpha _{1}({{\textbf{I}}}_{{\textbf{T}}}+{{\textbf{I}}}_{{\textbf{T}}{\textbf{C}}})+\alpha _{2}({{\textbf{E}}}_{{\textbf{C}}}+{{\textbf{I}}}_{{\textbf{C}}}+{{\textbf{L}}}_{{\textbf{T}}{\textbf{C}}}+{I}_{{\textbf{T}}{\textbf{C}}})+\beta \big )+\frac{\wp _{1}^{2}}{2}\nonumber \\ {}{} & {} =-\frac{\nabla }{\overline{{\textbf{S}}}}+\alpha _{1}({{\textbf{I}}}_{{\textbf{T}}}+{{\textbf{I}}}_{{\textbf{T}}{\textbf{C}}})+\alpha _{2}({{\textbf{E}}}_{{\textbf{C}}}+{{\textbf{I}}}_{{\textbf{C}}}+{{\textbf{L}}}_{{\textbf{T}}{\textbf{C}}}+{I}_{{\textbf{T}}{\textbf{C}}})-\frac{\nabla }{\overline{{\textbf{S}}}}\Bigg (\frac{1}{\widetilde{{\textbf{S}}}}-1\Bigg ). \end{aligned}$$

Applying the variant $\ln \varkappa \le \varkappa -1~(\forall ~\varkappa >0),$ we have $\ln \frac{1}{\widetilde{{\textbf{S}}}}\le \frac{1-\widetilde{{\textbf{S}}}}{\widetilde{{\textbf{S}}}}.$

Again, considering (36), we have

41 $$\begin{aligned} {\mathcal {L}}(-\ln {\textbf{S}}){} & {} \le -\frac{\nabla }{\widetilde{{\textbf{S}}}}\ln \frac{1}{\widetilde{{\textbf{S}}}}+\alpha _{1}({{\textbf{I}}}_{{\textbf{T}}}+{{\textbf{I}}}_{{\textbf{T}}{\textbf{C}}})+\alpha _{2}({{\textbf{E}}}_{{\textbf{C}}}+{{\textbf{I}}}_{{\textbf{C}}}+{{\textbf{L}}}_{{\textbf{T}}{\textbf{C}}}+{I}_{{\textbf{T}}{\textbf{C}}})\nonumber \\ {}{} & {} = \frac{\nabla }{\widetilde{{\textbf{S}}}}\ln {\widetilde{{\textbf{S}}}}+\alpha _{1}({{\textbf{I}}}_{{\textbf{T}}}+{{\textbf{I}}}_{{\textbf{T}}{\textbf{C}}})+\alpha _{2}({{\textbf{E}}}_{{\textbf{C}}}+{{\textbf{I}}}_{{\textbf{C}}}+{{\textbf{L}}}_{{\textbf{T}}{\textbf{C}}}+{I}_{{\textbf{T}}{\textbf{C}}}). \end{aligned}$$

Adopting the similar technique to $-\ln {{\textbf{L}}}_{{\textbf{T}}},~-\ln {{\textbf{L}}}_{{\textbf{T}}{\textbf{C}}},$ and $-\ln {{\textbf{R}}},$ respectively, we have

42 $$\begin{aligned} {\mathcal {L}}(-\ln {{\textbf{L}}}_{{\textbf{T}}}){} & {} \le \frac{\psi _{{\textbf{T}}}\tilde{{\textbf{S}}}}{\widetilde{{{\textbf{I}}}_{{\textbf{T}}}}}\ln {{{\textbf{I}}}_{{\textbf{T}}}}-\frac{\lambda \alpha _{2}{\widetilde{{\textbf{L}}}_{{\textbf{T}}{\textbf{C}}}}}{\widetilde{{\textbf{L}}}_{{\textbf{T}}}}\ln \widetilde{{\textbf{L}}}_{{\textbf{T}}{\textbf{C}}}+\frac{\lambda \psi _{{\textbf{C}}}}{\widetilde{{\textbf{L}}}_{{\textbf{T}}}}\ln \widetilde{{\textbf{R}}},\nonumber \\ {\mathcal {L}}(-\ln {{\textbf{L}}}_{{\textbf{T}}{\textbf{C}}}){} & {} \le \frac{\lambda \psi _{{\textbf{C}}}\widetilde{{{\textbf{I}}}_{{\textbf{T}}}}}{\widetilde{{{\textbf{L}}}_{{\textbf{T}}{\textbf{C}}}}}\ln \widetilde{{{\textbf{I}}}_{{\textbf{T}}}}+\frac{(\beta +\zeta _{{\textbf{C}}}+\rho +\eta )}{\widetilde{{\textbf{L}}}_{{\textbf{T}}{\textbf{C}}}}\ln \widetilde{{\textbf{L}}}_{{\textbf{T}}{\textbf{C}}}-\lambda \alpha _{2}\beta \frac{\ln \widetilde{{\textbf{R}}}}{\widetilde{{\textbf{R}}}},\nonumber \\ {\mathcal {L}}(-\ln {{\textbf{R}}}){} & {} \le -\frac{\varpi \widetilde{{{\textbf{L}}}_{{\textbf{T}}}}}{\widetilde{{\textbf{R}}}}\ln \widetilde{{{\textbf{L}}}_{{\textbf{T}}}}-(1-(\varsigma _{1}+\varsigma _{2}))\eta \frac{\widetilde{{{\textbf{L}}}_{{\textbf{T}}{\textbf{C}}}}}{\widetilde{{\textbf{R}}}}\ln \widetilde{{{\textbf{L}}}_{{\textbf{T}}{\textbf{C}}}}-\beta \frac{\ln \widetilde{{\textbf{R}}}}{\widetilde{{\textbf{R}}}}.\end{aligned}$$

Now, introducing a ${\mathbb {C}}^{2}$-mapping $\Phi _{1}$ as

$$\begin{aligned} \Phi _{1}(\Lambda )=-\ln {\textbf{S}}-{a_{1}}\ln {{\textbf{L}}}_{{\textbf{T}}}-{a_{2}}\ln {{\textbf{L}}}_{{\textbf{T}}{\textbf{C}}}-{a_{3}}\ln {{\textbf{R}}}, \end{aligned}$$

so that

43 $$\begin{aligned} {\left\{ \begin{array}{ll} {a_{1}}\frac{\psi _{{\textbf{T}}}\widetilde{{\textbf{S}}}}{\widetilde{{{\textbf{L}}}_{{\textbf{T}}}}}-a_{2}\frac{\lambda \psi _{{\textbf{C}}}\widetilde{{{\textbf{L}}}_{{\textbf{T}}}}}{\widetilde{{{\textbf{L}}}_{{\textbf{T}}{\textbf{C}}}}}-a_{3}\frac{\varpi \widetilde{{{\textbf{L}}}_{{\textbf{T}}}}}{\widetilde{{\textbf{R}}}}=0,\\ a_{1}\frac{\lambda \alpha _{2}{\widetilde{{\textbf{L}}}_{{\textbf{T}}{\textbf{C}}}}}{\widetilde{{\textbf{L}}}_{{\textbf{T}}}}+a_{2}\frac{(\beta +\zeta _{{\textbf{C}}}+\rho +\eta )}{\widetilde{{\textbf{L}}}_{{\textbf{T}}{\textbf{C}}}}-a_{3}(1-(\varsigma _{1}+\varsigma _{2}))\eta \frac{\widetilde{{{\textbf{L}}}_{{\textbf{T}}{\textbf{C}}}}}{\widetilde{{\textbf{R}}}}=0,\\ -a_{2}\lambda \alpha _{2}\beta -a_{3}\frac{\beta }{\widetilde{{\textbf{R}}}}+\frac{\lambda \psi _{{\textbf{C}}}}{\widetilde{{\textbf{L}}}_{{\textbf{T}}}}=0, \end{array}\right. } \end{aligned}$$

where

$$\begin{aligned} a_{1}=\frac{\lambda \psi _{{\textbf{C}}}^{2}\widetilde{{\textbf{L}}}_{{\textbf{T}}}}{\beta \alpha _{2}\psi _{{\textbf{T}}}\widetilde{{\textbf{S}}}\widetilde{{\textbf{L}}}_{{\textbf{T}}{\textbf{C}}}}-\frac{\widetilde{{\textbf{L}}}_{{\textbf{T}}}(\widetilde{{\textbf{R}}}\widetilde{{\textbf{L}}}_{{\textbf{T}}}\psi _{{\textbf{C}}}-\varpi \alpha _{2}\widetilde{{\textbf{L}}}_{{\textbf{T}}}\widetilde{{\textbf{R}}}\widetilde{{\textbf{L}}}_{{\textbf{T}}{\textbf{C}}})}{\alpha _{2}\psi _{{\textbf{T}}}\widetilde{{\textbf{S}}}\widetilde{{\textbf{R}}}^{2}\widetilde{{\textbf{L}}}_{{\textbf{T}}{\textbf{C}}}}\bigg (\frac{\lambda ^{2}}{\beta \psi _{{\textbf{T}}}\widetilde{{\textbf{L}}}_{{\textbf{T}}}\widetilde{{\textbf{S}}}}+\frac{\widetilde{{\textbf{L}}}_{{\textbf{C}}}\widetilde{{\textbf{R}}}(\beta +\zeta _{{\textbf{C}}}+\rho +\eta )}{\widetilde{{\textbf{L}}}_{{\textbf{T}}{\textbf{C}}}\widetilde{{\textbf{L}}}_{{\textbf{T}}}\widetilde{{\textbf{R}}}\alpha _{2\beta }}/{\mathcal {F}}_{2}\bigg ), \\ a_{2}=\frac{\psi _{{\textbf{C}}}}{\widetilde{{\textbf{L}}}_{{\textbf{T}}}\alpha _{2}\beta }-\frac{\lambda ^{2}\alpha _{2}\widetilde{{\textbf{L}}}_{{\textbf{T}}{\textbf{C}}}\widetilde{{\textbf{R}}}+\widetilde{{\textbf{R}}}\widetilde{{\textbf{L}}}_{{\textbf{C}}}(\beta +\zeta _{{\textbf{C}}}+\rho +\eta )}{\lambda \alpha ^{2}\widetilde{{\textbf{L}}}_{{\textbf{T}}}\widetilde{{\textbf{L}}}_{{\textbf{T}}{\textbf{C}}}\beta ^{2}\widetilde{{\textbf{S}}}\psi _{{\textbf{T}}}\widetilde{{\textbf{R}}}{\mathcal {F}}_{2}}, \\ a_{3}=\frac{\lambda ^{2}}{\beta \psi _{{\textbf{T}}}\widetilde{{\textbf{L}}}_{{\textbf{T}}}\widetilde{{\textbf{S}}}}+\frac{\widetilde{{\textbf{L}}}_{{\textbf{C}}}\widetilde{{\textbf{R}}}(\beta +\zeta _{{\textbf{C}}}+\rho +\eta )}{\widetilde{{\textbf{L}}}_{{\textbf{T}}{\textbf{C}}}\widetilde{{\textbf{L}}}_{{\textbf{T}}}\widetilde{{\textbf{R}}}\alpha _{2\beta }}/{\mathcal {F}}_{2}, \end{aligned}$$

where $${\mathcal {F}}_{2}=\frac{\widetilde{{\textbf{L}}}_{{\textbf{T}}{\textbf{C}}}(1-(\varsigma _{1}+\varsigma _{2}))\eta }{\widetilde{{\textbf{R}}}}+\frac{\lambda \alpha _{2}\widetilde{{\textbf{L}}}_{{\textbf{T}}{\textbf{C}}}(\widetilde{{\textbf{L}}}_{{\textbf{T}}}\widetilde{{\textbf{R}}}\psi _{{\textbf{C}}}-\varpi \alpha _{2}\widetilde{{\textbf{R}}}\widetilde{{\textbf{L}}}_{{\textbf{T}}}\widetilde{{\textbf{L}}}_{{\textbf{T}}{\textbf{C}}})}{\psi _{{\textbf{T}}}\widetilde{{\textbf{S}}}}+\frac{\widetilde{{\textbf{L}}}_{{\textbf{T}}}(\beta +\zeta _{{\textbf{C}}}+\rho +\eta )}{\beta \lambda \alpha _{2}\widetilde{{\textbf{T}}}_{{\textbf{T}}{\textbf{C}}}\widetilde{{\textbf{R}}}}.$$

Implementing the Itô’s technique to $\Phi _{1}$ and considering (42)–(44), we have

44 $$\begin{aligned} {\mathcal {L}}\Phi _{1}{} & {} \le \frac{\nabla }{\widetilde{{\textbf{S}}}}\ln {\widetilde{{\textbf{S}}}}+\Big ({a_{1}}\frac{\psi _{{\textbf{T}}}\widetilde{{\textbf{S}}}}{\widetilde{{{\textbf{L}}}_{{\textbf{T}}}}}-a_{2}\frac{\lambda \psi _{{\textbf{C}}}\widetilde{{{\textbf{L}}}_{{\textbf{T}}}}}{\widetilde{{{\textbf{L}}}_{{\textbf{T}}{\textbf{C}}}}}-a_{3}\frac{\varpi \widetilde{{{\textbf{L}}}_{{\textbf{T}}}}}{\widetilde{{\textbf{R}}}}\Big )\ln \widetilde{{{\textbf{L}}}_{{\textbf{T}}}}\nonumber \\ {}{} & {} \quad +\Big (a_{1}\frac{\lambda \alpha _{2}{\widetilde{{\textbf{L}}}_{{\textbf{T}}{\textbf{C}}}}}{\widetilde{{\textbf{L}}}_{{\textbf{T}}}}+a_{2}\frac{(\beta +\zeta _{{\textbf{C}}}+\rho +\eta )}{\widetilde{{\textbf{L}}}_{{\textbf{T}}{\textbf{C}}}}-a_{3}(1-(\varsigma _{1}+\varsigma _{2}))\eta \frac{\widetilde{{{\textbf{L}}}_{{\textbf{T}}{\textbf{C}}}}}{\widetilde{{\textbf{R}}}}\Big )\ln \widetilde{{{\textbf{L}}}_{{\textbf{T}}{\textbf{C}}}}\nonumber \\ {}{} & {} \quad -\Big (a_{2}\lambda \alpha _{2}\beta +a_{3}\frac{\beta }{\widetilde{{\textbf{R}}}}-\frac{\lambda \psi _{{\textbf{C}}}}{\widetilde{{\textbf{L}}}_{{\textbf{T}}}}\Big )\ln \widetilde{{\textbf{R}}}+\alpha _{1}({{\textbf{I}}}_{{\textbf{T}}}+{{\textbf{I}}}_{{\textbf{T}}{\textbf{C}}})+\alpha _{2}({{\textbf{E}}}_{{\textbf{C}}}+{{\textbf{I}}}_{{\textbf{C}}}+{{\textbf{L}}}_{{\textbf{T}}{\textbf{C}}}+{I}_{{\textbf{T}}{\textbf{C}}})\nonumber \\ {}{} & {} =\frac{\nabla }{\widetilde{{\textbf{S}}}}\ln {\widetilde{{\textbf{S}}}}+\alpha _{1}({{\textbf{I}}}_{{\textbf{T}}}+{{\textbf{I}}}_{{\textbf{T}}{\textbf{C}}})+\alpha _{2}({{\textbf{I}}}_{{\textbf{C}}}+{I}_{{\textbf{T}}{\textbf{C}}}). \end{aligned}$$

Then, we describe a ${\mathbb {C}}^{2}$-function $\Phi _{2}$ as

45 $$\begin{aligned} \Phi _{2}(\Lambda )=-\ln {{\textbf{L}}}_{{\textbf{T}}}-{{\textbf{b}}_{1}}\ln {{\textbf{S}}}-{{\textbf{b}}_{2}}\ln {{\textbf{L}}}_{{\textbf{T}}{\textbf{C}}}-{{\textbf{b}}_{3}}\ln {{\textbf{R}}}, \end{aligned}$$

which leads to

46 $$\begin{aligned} {\left\{ \begin{array}{ll} {{\textbf{b}}_{1}}\frac{\nabla }{\widetilde{{\textbf{S}}}}-\frac{\lambda \psi _{{\textbf{C}}}\widetilde{{{\textbf{L}}}_{{\textbf{T}}}}}{\widetilde{{{\textbf{L}}}_{{\textbf{T}}{\textbf{C}}}}}=0,\\ {\textbf{b}}_{2}\frac{(\beta +\zeta _{{\textbf{C}}}+\rho +\eta ) }{\widetilde{{{\textbf{L}}}_{{\textbf{T}}{\textbf{C}}}}}-{\textbf{b}}_{3}(1-(\varsigma _{1}+\varsigma _{2}))\eta \frac{\widetilde{{{\textbf{L}}}_{{\textbf{T}}{\textbf{C}}}}}{\widetilde{{\textbf{R}}}}=0,\\ {\textbf{b}}_{2}\frac{\lambda \alpha _{2}\beta }{\widetilde{{\textbf{R}}}}-{\textbf{b}}_{3}\frac{\beta }{\widetilde{{\textbf{R}}}}+\frac{\lambda \psi _{{\textbf{C}}}}{\widetilde{{\textbf{L}}}_{{\textbf{T}}}}=0, \end{array}\right. } \end{aligned}$$

where

$$\begin{aligned} {\textbf{b}}_{1}=\frac{\lambda \psi _{{\textbf{C}}}\widetilde{{\textbf{S}}}\widetilde{{{\textbf{L}}}_{{\textbf{T}}}}}{\nabla \widetilde{{{\textbf{L}}}_{{\textbf{T}}{\textbf{C}}}}}, \end{aligned}$$

$$\begin{aligned} {\textbf{b}}_{2}=\frac{\lambda \psi _{{\textbf{C}}}(1-(\varsigma _{1}+\varsigma _{2}))\eta \widetilde{{\textbf{L}}}_{{\textbf{T}}{\textbf{C}}}^{2}\widetilde{{\textbf{R}}}}{\beta (\beta +\zeta _{{\textbf{C}}}+\rho +\eta )\widetilde{L_{{\textbf{T}}}}\widetilde{{\textbf{R}}}-\lambda \alpha _{2}\beta }, \\ {\textbf{b}}_{3}=\frac{\lambda \psi _{{\textbf{C}}}(\beta +\zeta _{{\textbf{C}}}+\rho +\eta )\widetilde{{\textbf{R}}}}{\beta (\beta +\zeta _{{\textbf{C}}}+\rho +\eta )\widetilde{L_{{\textbf{T}}}}\widetilde{{\textbf{R}}}-\lambda \alpha _{2}\beta }. \end{aligned}$$

Implementing the Itô’s technique to $\Phi _{2}$ and considering (45)–(47), we have

47 $$\begin{aligned} {\mathcal {L}}\Phi _{2}(\Lambda )\le \bigg (\frac{(\beta +\zeta _{{\textbf{C}}}+\rho +\eta )}{\widetilde{{\textbf{L}}}_{{\textbf{T}}{\textbf{C}}}}-{\textbf{b}}_{3}(1-(\varsigma _{1}+\varsigma _{2}))\eta \frac{\widetilde{{\textbf{L}}}_{{\textbf{T}}{\textbf{C}}}}{\widetilde{{\textbf{R}}}}\bigg )\ln \widetilde{{{\textbf{L}}}_{{\textbf{T}}{\textbf{C}}}}+{\textbf{b}}_{1}\alpha _{1}{{\textbf{I}}}_{{\textbf{T}}}+\alpha _{2}({{\textbf{I}}}_{{\textbf{C}}}+{{\textbf{I}}}_{{\textbf{T}}{\textbf{C}}}). \end{aligned}$$

Furthermore, we indicate

$$\begin{aligned} \widetilde{{{\textbf{E}}}_{{\textbf{C}}}}=\frac{{{\textbf{E}}}_{{\textbf{C}}}}{\overline{{{\textbf{E}}}_{{\textbf{C}}}}},~~~\widetilde{{{\textbf{I}}}_{{\textbf{T}}}}=\frac{{{\textbf{I}}}_{{\textbf{T}}}}{\overline{{{\textbf{I}}}_{{\textbf{T}}}}},~~~\widetilde{{{\textbf{I}}}_{{\textbf{C}}}}=\frac{{{\textbf{I}}}_{{\textbf{C}}}}{\overline{{{\textbf{I}}}_{{\textbf{C}}}}},~~~\widetilde{{{\textbf{I}}}_{{\textbf{T}}{\textbf{C}}}}=\frac{{{\textbf{I}}}_{{\textbf{T}}{\textbf{C}}}}{\overline{{{\textbf{I}}}_{{\textbf{T}}{\textbf{C}}}}}. \end{aligned}$$

Implementing Itô’s technique to $-\ln {{{\textbf{E}}}_{{\textbf{C}}}},$ one obtains

48 $$\begin{aligned} {\mathcal {L}}(-\ln {{\textbf{E}}}_{{\textbf{C}}}){} & {} \le -\frac{\psi _{{\textbf{C}}}{{\textbf{S}}}}{{{{\textbf{E}}}_{{\textbf{C}}}}}+{(\beta +\epsilon \psi _{{\textbf{T}}}+\varphi _{1}+\varphi _{2})}+\frac{\wp _{4}^{2}}{2}\nonumber \\ {}{} & {} \le -\frac{\psi _{{\textbf{C}}}{{\textbf{S}}}}{{{{\textbf{E}}}_{{\textbf{C}}}}}+{(\beta +\epsilon \psi _{{\textbf{T}}}+\varphi _{1}+\varphi _{2})}+\frac{\wp _{4}^{2}}{2}-\frac{\psi _{{\textbf{C}}}\widetilde{{\textbf{S}}}}{\widetilde{{{\textbf{E}}}_{{\textbf{C}}}}}\Big (\frac{\widetilde{{\textbf{S}}}}{\widetilde{{{\textbf{E}}}_{{\textbf{C}}}}}-1\Big )\nonumber \\ {}{} & {} \le -({\mathbb {R}}_{0}^{{\mathbb {S}}}-1)\big ({(\beta +\epsilon \psi _{{\textbf{T}}}+\varphi _{1}+\varphi _{2})}+\frac{\wp _{4}^{2}}{2}\big )+\frac{\psi _{{\textbf{C}}}\widetilde{{\textbf{S}}}}{\widetilde{{{\textbf{E}}}_{{\textbf{C}}}}}\ln \widetilde{{{\textbf{E}}}_{{\textbf{C}}}}-\frac{\psi _{{\textbf{C}}}\widetilde{{\textbf{S}}}}{\widetilde{{{\textbf{E}}}_{{\textbf{C}}}}}\ln \widetilde{{\textbf{S}}}, \end{aligned}$$

where

$$\begin{aligned} {\mathbb {R}}_{0}^{{\mathbb {S}}}=\frac{\psi _{{\textbf{C}}}\widetilde{{\textbf{S}}}}{{(\beta +\epsilon \psi _{{\textbf{T}}}+\varphi _{1}+\varphi _{2})}+\frac{\wp _{4}^{2}}{2}}. \end{aligned}$$

Analogously, implementing Itô’s technique to $-\ln {{\textbf{I}}}_{{\textbf{T}}},~~-\ln {{\textbf{I}}}_{{\textbf{C}}}$ and $-\ln {{\textbf{I}}}_{{\textbf{T}}{\textbf{C}}},$ we find

49 $$\begin{aligned}{} & {} {\mathcal {L}}(-\ln {{\textbf{I}}}_{{\textbf{T}}})\le \mu \frac{\widetilde{{{\textbf{L}}}_{{\textbf{T}}}}}{\widetilde{{\textbf{I}}}_{{\textbf{T}}}}\ln \widetilde{{{\textbf{L}}}_{{\textbf{T}}}}-\varsigma _{2}\eta \frac{\widetilde{{{\textbf{L}}}_{{\textbf{T}}{\textbf{C}}}}}{\widetilde{{\textbf{I}}}_{{\textbf{T}}}}\ln \widetilde{{{\textbf{L}}}_{{\textbf{T}}{\textbf{C}}}}-\theta _{2}\xi \frac{\widetilde{{{\textbf{I}}}_{{\textbf{T}}{\textbf{C}}}}}{\widetilde{{\textbf{I}}}_{{\textbf{T}}}}\ln \widetilde{{{\textbf{I}}}_{{\textbf{T}}{\textbf{C}}}}+(\beta +\varsigma _{3}+\zeta _{{\textbf{T}}}+\delta )\frac{1}{\widetilde{{\textbf{I}}}_{{\textbf{T}}}}\widetilde{{\textbf{I}}}_{{\textbf{T}}},\nonumber \\ {}{} & {} {\mathcal {L}}(-\ln {{\textbf{I}}}_{{\textbf{C}}})\le \varphi _{1}\frac{\widetilde{{{\textbf{E}}}_{{\textbf{C}}}}}{\widetilde{{\textbf{I}}}_{{\textbf{C}}}}\ln \widetilde{{{\textbf{E}}}_{{\textbf{C}}}}-\rho \eta \frac{\widetilde{{{\textbf{L}}}_{{\textbf{T}}{\textbf{C}}}}}{\widetilde{{\textbf{I}}}_{{\textbf{C}}}}\ln \widetilde{{{\textbf{L}}}_{{\textbf{T}}{\textbf{C}}}}-\theta _{1}\xi \frac{\widetilde{{{\textbf{I}}}_{{\textbf{T}}{\textbf{C}}}}}{\widetilde{{\textbf{I}}}_{{\textbf{C}}}}\ln \widetilde{{{\textbf{I}}}_{{\textbf{T}}{\textbf{C}}}}+(\beta +\zeta _{{\textbf{C}}}+\nu +\varphi _{3})\frac{1}{\widetilde{{\textbf{I}}}_{{\textbf{C}}}}\ln \widetilde{{\textbf{I}}}_{{\textbf{C}}},\nonumber \\ {}{} & {} {\mathcal {L}}(-\ln {{\textbf{I}}}_{{\textbf{T}}{\textbf{C}}})\le \rho \frac{\widetilde{{{\textbf{L}}}_{{\textbf{T}}{\textbf{C}}}}}{\widetilde{{\textbf{I}}}_{{\textbf{T}}{\textbf{C}}}}\ln \widetilde{{{\textbf{L}}}_{{\textbf{T}}{\textbf{C}}}}-\varsigma _{3}\frac{\widetilde{{{\textbf{I}}}_{{\textbf{T}}}}}{\widetilde{{\textbf{I}}}_{{\textbf{T}}{\textbf{C}}}}\ln \widetilde{{{\textbf{I}}}_{{\textbf{T}}}}-\nu \frac{\widetilde{{{\textbf{I}}}_{{\textbf{C}}}}}{\widetilde{{\textbf{I}}}_{{\textbf{T}}{\textbf{C}}}}\ln \widetilde{{{\textbf{I}}}_{{\textbf{C}}}}+(\beta +\zeta _{{\textbf{T}}{\textbf{C}}}+\xi )\frac{1}{\widetilde{{\textbf{I}}}_{{\textbf{T}}{\textbf{C}}}}\ln \widetilde{{\textbf{I}}}_{{\textbf{T}}{\textbf{C}}}.\nonumber \\ \end{aligned}$$

Introducing

50 $$\begin{aligned} \Phi _{3}(\Lambda )=-\ln {{\textbf{E}}}_{{\textbf{C}}}-{\textbf{c}}_{1}\ln {{\textbf{I}}}_{{\textbf{T}}}-{\textbf{c}}_{2}\ln {{{\textbf{I}}}_{{\textbf{C}}}}-{\textbf{c}}_{3}{{\textbf{I}}}_{{\textbf{T}}{\textbf{C}}}, \end{aligned}$$

which leads to

51 $$\begin{aligned} {\left\{ \begin{array}{ll} {\textbf{c}}_{1}\frac{(\beta +\varsigma _{3}+\zeta _{{\textbf{T}}}+\delta )}{\widetilde{{\textbf{I}}}_{{\textbf{T}}}}-{\textbf{c}}_{3}\frac{\varsigma _{3}}{\widetilde{{{\textbf{I}}}}_{{\textbf{T}}{\textbf{C}}}}-\frac{\psi _{{\textbf{C}}\widetilde{{\textbf{S}}}}}{\widetilde{{\textbf{E}}}_{{\textbf{C}}}}=0,\\ {\textbf{c}}_{2}\frac{\beta +\zeta _{{\textbf{C}}}+\nu +\varphi _{3}}{\widetilde{{{\textbf{I}}}_{{\textbf{C}}}}}-{\textbf{c}}_{3}\nu \frac{\widetilde{{{\textbf{I}}}_{{\textbf{C}}}}}{\widetilde{{\textbf{I}}}_{{\textbf{T}}{\textbf{C}}}}=0,\\ -{\textbf{c}}_{1}\theta _{2}\xi \frac{\widetilde{{{\textbf{I}}}_{{\textbf{T}}{\textbf{C}}}}}{\widetilde{{{\textbf{I}}}_{{\textbf{T}}}}}-{\textbf{c}}_{2}\theta _{1}\xi \frac{\widetilde{{{\textbf{I}}}_{{\textbf{T}}{\textbf{C}}}}}{\widetilde{{{\textbf{I}}}_{{\textbf{C}}}}}+{\textbf{c}}_{3}(\beta +\zeta _{{\textbf{T}}{\textbf{C}}}+\xi )=0, \end{array}\right. } \end{aligned}$$

where

$$\begin{aligned} {\textbf{c}}_{1}=\frac{\psi _{{\textbf{T}}{\textbf{C}}}\widetilde{{{\textbf{I}}}_{{\textbf{T}}}}\widetilde{{{\textbf{I}}}_{{\textbf{T}}{\textbf{C}}}}\big ((\beta +\zeta _{{\textbf{T}}{\textbf{C}}}+\xi )(\beta +\zeta _{{\textbf{C}}}+\nu +\varphi _{3})-\theta _{1}\xi \nu \widetilde{{{\textbf{I}}}_{{\textbf{C}}}}\big )}{{\mathcal {F}}_{3}}, \end{aligned}$$

$$\begin{aligned} {\textbf{c}}_{2}=\frac{\nu (\beta +\varsigma _{3}+\zeta _{\tau }+\delta )\widetilde{{{\textbf{I}}}_{{\textbf{C}}}}^{2}}{\varsigma _{3}(\beta +\zeta _{{\textbf{C}}}+\nu +\xi )\widetilde{{{\textbf{I}}}_{{\textbf{T}}}}}\bigg (\frac{\psi _{{\textbf{T}}{\textbf{C}}}\widetilde{{{\textbf{I}}}_{{\textbf{T}}}}\widetilde{{{\textbf{I}}}_{{\textbf{T}}{\textbf{C}}}}\big ((\beta +\zeta _{{\textbf{T}}{\textbf{C}}}+\xi )(\beta +\zeta _{{\textbf{C}}}+\nu +\varphi _{3})-\theta _{1}\xi \nu \widetilde{{{\textbf{I}}}_{{\textbf{C}}}}\big )}{{\mathcal {F}}_{3}}\bigg )-\frac{\nu \psi _{C_ {1}}\widetilde{{\textbf{S}}}\widetilde{{{\textbf{I}}}_{{\textbf{C}}}^{2}}}{\varsigma _{3}(\beta +\zeta _{{\textbf{C}}}+\nu +\varphi _{3})\widetilde{{{\textbf{E}}}_{{\textbf{C}}}}}, \end{aligned}$$

$$\begin{aligned} {\textbf{c}}_{3}=\frac{(\beta +\varsigma _{3}+\zeta _{{\textbf{T}}}+\delta )\widetilde{{{\textbf{I}}}_{{\textbf{T}}{\textbf{C}}}}}{\varsigma _{3}\widetilde{{{\textbf{I}}}_{{\textbf{T}}}}}\bigg (\frac{\psi _{{\textbf{T}}{\textbf{C}}}\widetilde{{{\textbf{I}}}_{{\textbf{T}}}}\widetilde{{{\textbf{I}}}_{{\textbf{T}}{\textbf{C}}}}\big ((\beta +\zeta _{{\textbf{T}}{\textbf{C}}}+\xi )(\beta +\zeta _{{\textbf{C}}}+\nu +\varphi _{3})-\theta _{1}\xi \nu \widetilde{{{\textbf{I}}}_{{\textbf{C}}}}\big )}{{\mathcal {F}}_{3}}\bigg )-\frac{\psi _{{\textbf{C}}}\widetilde{{\textbf{S}}}\widetilde{{{\textbf{I}}}_{{\textbf{T}}{\textbf{C}}}}}{\varsigma _{3}}. \end{aligned}$$

As $${\mathcal {F}}_{3}=\varsigma _{3}\psi _{{\textbf{C}}}\widetilde{{\textbf{S}}}(\beta +\zeta _{{\textbf{C}}}+\nu +\varphi _{3})-\theta _{1}\xi \nu (\beta +\varsigma _{3}+\zeta _{{\textbf{T}}}+\delta )\widetilde{{{\textbf{I}}}_{{\textbf{C}}}}\widetilde{{{\textbf{I}}}_{{\textbf{T}}{\textbf{C}}}}\widetilde{{{\textbf{E}}}_{{\textbf{C}}}}+(\beta +\zeta _{{\textbf{T}}{\textbf{C}}}+\xi )(\beta +\varsigma _{3}+\zeta _{{\textbf{T}}}+\delta )(\beta +\zeta _{{\textbf{C}}}+\nu +\varphi _{3})\widetilde{{{\textbf{E}}}_{{\textbf{C}}}}\widetilde{{{\textbf{I}}}_{{\textbf{T}}{\textbf{C}}}}.$$

Now, considering (49)–(52), we have

52 $$\begin{aligned} {\mathcal {L}}\Phi _{3}(\Lambda )=-({\mathbb {R}}_{0}^{{\mathbb {S}}}-1)\Big (\beta +\epsilon \psi _{{\textbf{T}}}+\varphi _{1}+\varphi _{2}+\frac{\wp _{4}^{2}}{2}\Big )+\frac{\psi _{{\textbf{C}}}\widetilde{{\textbf{S}}}}{\widetilde{{{\textbf{E}}}_{{\textbf{C}}}}}\ln \widetilde{{{\textbf{E}}}_{{\textbf{C}}}}-\frac{\psi _{{\textbf{C}}}\widetilde{{\textbf{S}}}}{\widetilde{{{\textbf{E}}}_{{\textbf{C}}}}}\ln \widetilde{{\textbf{S}}}. \end{aligned}$$

Furthermore, we describe

53 $$\begin{aligned} \Phi _{4}(\Lambda )=\Phi _{3}(\Lambda )+{\textbf{d}}_{1}\Phi _{1}(\Lambda )+{\textbf{d}}_{2}\Phi _{2}(\Lambda ). \end{aligned}$$

Thus, we conclude that ${\textbf{d}}_{1}=\frac{\alpha _{1}\psi _{{\textbf{C}}}\widetilde{{\textbf{S}}}^{2}\widetilde{{{\textbf{I}}}_{{\textbf{T}}{\textbf{C}}}\widetilde{{\textbf{L}}}_{{\textbf{T}}}}}{(\nabla \widetilde{{\textbf{L}}}_{{\textbf{T}}}+a_{1}\widetilde{{{\textbf{S}}}})\widetilde{{{\textbf{E}}}_{{\textbf{C}}}}}$ and ${\textbf{d}}_{2}=\frac{{\textbf{c}}_{1}\alpha _{2}\psi _{{\textbf{C}}}\widetilde{{\textbf{S}}}\widetilde{{{\textbf{L}}}_{{\textbf{T}}{\textbf{C}}}^{2}\widetilde{{\textbf{I}}}_{{\textbf{C}}}}}{(\lambda \psi _{{\textbf{C}}}\widetilde{{\textbf{L}}}_{{\textbf{T}}{\textbf{C}}}+{\textbf{b}}_{1}\nabla \widetilde{{{\textbf{I}}}}_{{\textbf{T}}{\textbf{C}}})\widetilde{{{\textbf{E}}}_{{\textbf{C}}}}}.$

In view of (45), (48) and (53), we have

54 $$\begin{aligned} {\mathcal {L}}\Phi _{4}(\Lambda )\le -({\mathbb {R}}_{0}^{{\mathbb {S}}}-1)\Big (\beta +\epsilon \psi _{{\textbf{T}}}+\varphi _{1}+\varphi _{2}+\frac{\wp _{4}^{2}}{2}\Big )+\alpha _{1}({\textbf{d}}_{1}+{\textbf{b}}_{1}{\textbf{d}}_{2}){{\textbf{I}}}_{{\textbf{T}}}+\alpha _{2}(a_{2}{\textbf{d}}_{1}+{\textbf{d}}_{2}){{\textbf{I}}}_{{\textbf{C}}}. \end{aligned}$$

Introducing

55 $$\begin{aligned} \Phi _{5}(\Lambda )=\Phi _{4}(\Lambda )-\frac{\alpha _{2}(a_{2}{\textbf{d}}_{1}+{\textbf{d}}_{2})}{\theta _{2}\xi }{{\textbf{I}}}_{{\textbf{T}}}. \end{aligned}$$

Again, implementing the Itô’s technique to $\Phi _{5},$ we have

56 $$\begin{aligned} {\mathcal {L}}\Phi _{5}(\Lambda )\le -({\mathbb {R}}_{0}^{{\mathbb {S}}}-1)\Big (\beta +\epsilon \psi _{{\textbf{T}}}+\varphi _{1}+\varphi _{2}+\frac{\wp _{4}^{2}}{2}\Big )+\big [\alpha _{1}({\textbf{d}}_{1}+{\textbf{b}}_{1}{\textbf{d}}_{2})+\frac{\alpha _{2}(a_{2}{\textbf{d}}_{1}+{\textbf{d}}_{2})(\beta +\varsigma _{3}+\zeta _{{\textbf{T}}}+\delta )}{\theta _{2}\xi }\big ]{{\textbf{I}}}_{{\textbf{T}}}. \end{aligned}$$

Introducing

57 $$\begin{aligned} \Phi _{6}(\Lambda )=-\ln {{\textbf{S}}}-\ln {{\textbf{L}}}_{{\textbf{T}}}-\ln {{\textbf{E}}}_{{\textbf{C}}}-\ln {{\textbf{I}}}_{{\textbf{C}}}-\ln {{\textbf{L}}}_{{\textbf{T}}{\textbf{C}}}-\ln {{\textbf{I}}}_{{\textbf{T}}{\textbf{C}}}-\ln {{\textbf{R}}}. \end{aligned}$$

Implementing the Itô’s technique to $\Phi _{6},$ we have

58 $$\begin{aligned} {\mathcal {L}}\Phi _{6}(\Lambda ){} & {} \le -\frac{\nabla }{{\textbf{S}}}-\psi _{{\textbf{T}}}\frac{{\textbf{S}}}{{{\textbf{L}}}_{{\textbf{T}}}}-\psi _{{\textbf{C}}}\frac{{\textbf{S}}}{{{\textbf{E}}}_{{\textbf{C}}}}-\varphi _{1}\frac{{{\textbf{E}}}_{{\textbf{C}}}}{{{\textbf{I}}}_{{\textbf{C}}}}-\varsigma _{1}\eta \frac{{{\textbf{L}}}_{{\textbf{T}}{\textbf{C}}}}{{{\textbf{I}}}_{{\textbf{C}}}}-\theta _{1}\xi \frac{{{\textbf{I}}}_{{\textbf{T}}{\textbf{C}}}}{{{\textbf{I}}}_{{\textbf{C}}}}-\lambda \psi _{{\textbf{C}}}\frac{{{\textbf{L}}}_{{\textbf{T}}}}{{{\textbf{L}}}_{{\textbf{T}}{\textbf{C}}}}-\epsilon \psi _{{\textbf{T}}}\frac{{{\textbf{E}}}_{{\textbf{C}}}}{{{\textbf{L}}}_{{\textbf{T}}{\textbf{C}}}}\nonumber \\ {}{} & {} \quad -\rho \frac{{{\textbf{L}}}_{{\textbf{T}}{\textbf{C}}}}{{{\textbf{I}}}_{{\textbf{T}}{\textbf{C}}}}-\varsigma _{3}\frac{{{\textbf{I}}}_{{\textbf{T}}}}{{{\textbf{I}}}_{{\textbf{T}}{\textbf{C}}}}-\nu \frac{{{\textbf{I}}}_{{\textbf{C}}}}{{{\textbf{I}}}_{{\textbf{T}}{\textbf{C}}}}-\varpi \frac{{{\textbf{L}}}_{{\textbf{T}}}}{{\textbf{R}}}-\varphi _{2}\frac{{{\textbf{E}}}{{\textbf{C}}}}{{\textbf{R}}}-\delta \frac{{{\textbf{I}}}_{{\textbf{T}}}}{{\textbf{R}}}-\varphi _{3}\frac{{{\textbf{I}}}_{{\textbf{C}}}}{{\textbf{R}}}+(\psi _{{\textbf{T}}}+\psi _{{\textbf{C}}}+7\beta )\nonumber \\ {}{} & {} \quad +(\mu +\lambda \psi _{{\textbf{C}}}+\varpi +\epsilon \psi _{{\textbf{T}}}+\varphi _{1}+\varphi _{2}+2\zeta _{{\textbf{C}}}+\nu +\varphi _{3}+\rho +\eta +\zeta _{{\textbf{T}}{\textbf{C}}}+\xi )+\frac{1}{2}(\wp _{1}^{2}+\wp _{2}^{2}+\wp _{3}^{2}\nonumber \\ {}{} & {} \quad +\wp _{4}^{2}+\wp _{5}^{2}+\wp _{6}^{2}+\wp _{7}^{2}+\wp _{8}^{2}). \end{aligned}$$

Again, we describe

59 $$\begin{aligned} \Phi _{7}(\Lambda )=\frac{1}{\mu +1}\big ({{\textbf{S}}}+{{\textbf{L}}}_{{\textbf{T}}}+{{\textbf{I}}}_{{\textbf{T}}}+{{\textbf{E}}}_{{\textbf{C}}}+{{\textbf{I}}}_{{\textbf{C}}}+{{\textbf{L}}}_{{\textbf{T}}{\textbf{C}}}+{{\textbf{I}}}_{{\textbf{T}}{\textbf{C}}}+{{\textbf{R}}}\big )^{\mu +1},\end{aligned}$$

where $$\mu \in (0,1)$$ fulfilling

$$\begin{aligned} (\beta +\psi _{{\textbf{T}}})\wedge (\beta +\psi _{{\textbf{C}}})-\frac{\mu }{2}(\wp _{1}^{2}\vee \wp _{2}^{2}\vee \wp _{3}^{2}\vee \wp _{4}^{2}\vee \wp _{5}^{2}\vee \wp _{6}^{2}\vee \wp _{7}^{2}\vee \wp _{8}^{2})>0. \end{aligned}$$

Employing the Itô’s technique to $\Phi _{7},$ we have

60 $$\begin{aligned} {\mathcal {L}}\Phi _{7}(\Lambda ){} & {} =\big ({{\textbf{S}}}+{{\textbf{L}}}_{{\textbf{T}}}+{{\textbf{I}}}_{{\textbf{T}}}+{{\textbf{E}}}_{{\textbf{C}}}+{{\textbf{I}}}_{{\textbf{C}}}+{{\textbf{L}}}_{{\textbf{T}}{\textbf{C}}}+{{\textbf{I}}}_{{\textbf{T}}{\textbf{C}}}+{{\textbf{R}}}\big )^{\mu }\nonumber \\ {}{} & {} \quad \times \big [\nabla -(\beta +\psi _{{\textbf{T}}})({{\textbf{S}}}+{{\textbf{E}}}_{{\textbf{C}}}+{{\textbf{I}}}_{{\textbf{T}}}+{{\textbf{L}}}_{{\textbf{T}}})-\beta {{\textbf{I}}}_{{\textbf{T}}}-(\beta +\psi _{{\textbf{C}}})({{\textbf{I}}}_{{\textbf{C}}}+{{\textbf{L}}}_{{\textbf{T}}{\textbf{C}}}+{{\textbf{I}}}_{{\textbf{T}}{\textbf{C}}}\nonumber \\ {}{} & {} \quad +{{\textbf{R}}})-\beta {{\textbf{I}}}_{{\textbf{C}}}\big ]+\frac{\mu }{2}\big ({{\textbf{S}}}+{{\textbf{L}}}_{{\textbf{T}}}+{{\textbf{I}}}_{{\textbf{T}}}+{{\textbf{E}}}_{{\textbf{C}}}+{{\textbf{I}}}_{{\textbf{C}}}+{{\textbf{L}}}_{{\textbf{T}}{\textbf{C}}}+{{\textbf{I}}}_{{\textbf{T}}{\textbf{C}}}+{{\textbf{R}}}\big )^{\mu -1}\nonumber \\ {}{} & {} \quad \times \big (\wp _{1}^{2}{{\textbf{S}}}^{2}+\wp _{2}^{2}{{\textbf{L}}}_{{\textbf{T}}}^{2}+\wp _{3}^{2}{{\textbf{I}}}_{{\textbf{T}}}^{2}+\wp _{4}^{2}{{\textbf{E}}}_{{\textbf{C}}}^{2}+\wp _{5}^{2}{{\textbf{I}}}_{{\textbf{C}}}^{2}+\wp _{6}^{2}{{\textbf{L}}}_{{\textbf{T}}{\textbf{C}}}^{2}+\wp _{7}^{2}{{\textbf{I}}}_{{\textbf{T}}{\textbf{C}}}^{2}+\wp _{8}^{2}{{\textbf{R}}}^{2}\big )\nonumber \\ {}{} & {} \le \big ({{\textbf{S}}}+{{\textbf{L}}}_{{\textbf{T}}}+{{\textbf{I}}}_{{\textbf{T}}}+{{\textbf{E}}}_{{\textbf{C}}}+{{\textbf{I}}}_{{\textbf{C}}}+{{\textbf{L}}}_{{\textbf{T}}{\textbf{C}}}+{{\textbf{I}}}_{{\textbf{T}}{\textbf{C}}}+{{\textbf{R}}}\big )^{\mu }\nonumber \\ {}{} & {} \quad \times \Big [\nabla -[(\beta )+\psi _{{\textbf{T}}}\wedge (\beta +\psi _{{\textbf{C}}})]\big ({{\textbf{S}}}+{{\textbf{L}}}_{{\textbf{T}}}+{{\textbf{I}}}_{{\textbf{T}}}+{{\textbf{E}}}_{{\textbf{C}}}+{{\textbf{I}}}_{{\textbf{C}}}+{{\textbf{L}}}_{{\textbf{T}}{\textbf{C}}}+{{\textbf{I}}}_{{\textbf{T}}{\textbf{C}}}+{{\textbf{R}}}\big )\Big ]\nonumber \\ {}{} & {} \quad +\frac{\mu }{2}\big ({{\textbf{S}}}+{{\textbf{L}}}_{{\textbf{T}}}+{{\textbf{I}}}_{{\textbf{T}}}+{{\textbf{E}}}_{{\textbf{C}}}+{{\textbf{I}}}_{{\textbf{C}}}+{{\textbf{L}}}_{{\textbf{T}}{\textbf{C}}}+{{\textbf{I}}}_{{\textbf{T}}{\textbf{C}}}+{{\textbf{R}}}\big )^{\mu +1}\nonumber \\ {}{} & {} \quad \times \big (\wp _{1}^{2}\vee \wp _{2}^{2}\vee \wp _{3}^{2}\vee \wp _{4}^{2}\vee \wp _{5}^{2}\vee \wp _{6}^{2}\vee \wp _{7}^{2}\vee \wp _{8}^{2}\big )\nonumber \\ {}{} & {} \le {\textbf{Q}}-\frac{{\tilde{\mu }}}{2}\big ({{\textbf{S}}}+{{\textbf{L}}}_{{\textbf{T}}}+{{\textbf{I}}}_{{\textbf{T}}}+{{\textbf{E}}}_{{\textbf{C}}}+{{\textbf{I}}}_{{\textbf{C}}}+{{\textbf{L}}}_{{\textbf{T}}{\textbf{C}}}+{{\textbf{I}}}_{{\textbf{T}}{\textbf{C}}}+{{\textbf{R}}}\big )^{\mu +1}, \end{aligned}$$

where

61 $$\begin{aligned} {\textbf{Q}}{} & {} =\sup \limits _{\Lambda \in {\mathbb {R}}_{+}^{8}}\Bigg \{\nabla \bigg ({{\textbf{S}}}+{{\textbf{L}}}_{{\textbf{T}}}+{{\textbf{I}}}_{{\textbf{T}}}+{{\textbf{E}}}_{{\textbf{C}}}+{{\textbf{I}}}_{{\textbf{C}}}+{{\textbf{L}}}_{{\textbf{T}}{\textbf{C}}}+{{\textbf{I}}}_{{\textbf{T}}{\textbf{C}}}+{{\textbf{R}}}\bigg )^{\mu }\nonumber \\ {}{} & {} \quad -\frac{{\tilde{\mu }}}{2}\big ({{\textbf{S}}}+{{\textbf{L}}}_{{\textbf{T}}}+{{\textbf{I}}}_{{\textbf{T}}}+{{\textbf{E}}}_{{\textbf{C}}}+{{\textbf{I}}}_{{\textbf{C}}}+{{\textbf{L}}}_{{\textbf{T}}{\textbf{C}}}+{{\textbf{I}}}_{{\textbf{T}}{\textbf{C}}}+{{\textbf{R}}}\bigg )^{\mu +1}\Bigg \}, \end{aligned}$$

and

62 $$\begin{aligned} {\tilde{\mu }}=(\beta +\psi _{{\textbf{T}}})\wedge (\beta +\psi _{{\textbf{C}}})-\frac{\mu }{2}(\wp _{1}^{2}\vee \wp _{2}^{2}\vee \wp _{3}^{2}\vee \wp _{4}^{2}\vee \wp _{5}^{2}\vee \wp _{6}^{2}\vee \wp _{7}^{2}\vee \wp _{8}^{2}). \end{aligned}$$

Here, introducing a ${\mathbb {C}}^{2}$-function $\Phi _{8}$ on ${\mathbb {R}}_{+}^{8}\mapsto {\mathbb {R}}$

63 $$\begin{aligned} \Phi _{8}(\Lambda )={\mathbb {M}}\Phi _{5}(\Lambda )+\Phi _{6}(\Lambda )+\Phi _{7}(\Lambda ), 
\end{aligned}$$

where ${\mathbb {M}}$ is a sufficiently significant non-negative constant that satisfies

64 $$\begin{aligned} -{\mathbb {M}}({\mathbb {R}}_{0}^{{\mathbb {S}}}-1)\Bigg (\beta +\epsilon \psi _{{\textbf{T}}}+\varphi _{1}+\varphi _{2}+\frac{\wp {4}^{2}}{2}\Bigg )+{\mathcal {W}}\le -2, \end{aligned}$$

and

65 $$\begin{aligned} {\mathcal {W}}{} & {} =\sup \limits _{\Lambda \in {\mathbb {R}}_{+}^{8}}\Bigg \{(\psi _{{\textbf{T}}}+\psi _{{\textbf{C}}}+7\beta )+(\mu +\lambda \psi _{{\textbf{C}}}+\varpi +\epsilon \psi _{{\textbf{T}}}+\varphi _{1}+\varphi _{2}+2\zeta _{{\textbf{C}}}+\nu +\varphi _{3}+\rho +\eta +\zeta _{{\textbf{T}}{\textbf{C}}}+\xi )\nonumber \\ {}{} & {} \quad +\frac{1}{2}(\wp _{1}^{2}+\wp _{2}^{2}+\wp _{3}^{2}+\wp _{4}^{2}+\wp _{5}^{2}+\wp _{6}^{2}+\wp _{7}^{2}+\wp _{8}^{2})+{\textbf{Q}}+\alpha _{1}{{\textbf{I}}}_{{\textbf{T}}}+\alpha _{2}{{\textbf{I}}}_{{\textbf{C}}}\nonumber \\ {}{} & {} \quad -\frac{{\tilde{\mu }}}{2}\bigg ({{\textbf{S}}}+{{\textbf{L}}}_{{\textbf{T}}}+{{\textbf{I}}}_{{\textbf{T}}}+{{\textbf{E}}}_{{\textbf{C}}}+{{\textbf{I}}}_{{\textbf{C}}}+{{\textbf{L}}}_{{\textbf{T}}{\textbf{C}}}+{{\textbf{I}}}_{{\textbf{T}}{\textbf{C}}}+{{\textbf{R}}}\bigg )^{\mu +1}\Bigg \}. \end{aligned}$$

Examine that the minimal point ${\tilde{\Lambda }}\in {\mathbb {R}}_{+}^{8}$ of $\Phi _{8}(\Lambda )$ appears to exist, therefore we conclude

66 $$\begin{aligned} \Phi (\Lambda )=\Phi _{8}(\Lambda )-\Phi _{8}({\tilde{\Lambda }}). \end{aligned}$$

Merging (57), (59) and (61), we have

67 $$\begin{aligned} {\mathcal {L}}\Phi (\Lambda ){} & {} \le -{\mathcal {M}}({\mathbb {R}}_{0}^{{\mathbb {S}}}-1)\Big (\beta +\epsilon \psi _{{\textbf{T}}}+\varphi _{1}+\varphi _{2}+\frac{\wp {4}^{2}}{2}\Big )+{\mathcal {W}}\nonumber \\ {}{} & {} \quad +{\mathbb {M}}\big [\alpha _{1}({\textbf{d}}_{1}+{\textbf{b}}_{1}{\textbf{d}}_{2})+\frac{\alpha _{2}(a_{2}{\textbf{d}}_{1}+{\textbf{d}}_{2})(\beta +\varsigma _{3}+\zeta _{{\textbf{T}}}+\delta )}{\theta _{2}\xi }\big ]{{\textbf{I}}}_{{\textbf{T}}}-\frac{\nabla }{{\textbf{S}}}-\psi _{{\textbf{T}}}\frac{{\textbf{S}}}{{{\textbf{L}}}_{{\textbf{T}}}}\nonumber \\ {}{} & {} \quad -\psi _{{\textbf{C}}}\frac{{\textbf{S}}}{{{\textbf{E}}}_{{\textbf{C}}}}-\varphi _{1}\frac{{{\textbf{E}}}_{{\textbf{C}}}}{{{\textbf{I}}}_{{\textbf{C}}}}-\varsigma _{1}\eta \frac{{{\textbf{L}}}_{{\textbf{T}}{\textbf{C}}}}{{{\textbf{I}}}_{{\textbf{C}}}}-\theta _{1}\xi \frac{{{\textbf{I}}}_{{\textbf{T}}{\textbf{C}}}}{{{\textbf{I}}}_{{\textbf{C}}}}-\lambda \psi _{{\textbf{C}}}\frac{{{\textbf{L}}}_{{\textbf{T}}}}{{{\textbf{L}}}_{{\textbf{T}}{\textbf{C}}}}-\epsilon \psi _{{\textbf{T}}}\frac{{{\textbf{E}}}_{{\textbf{C}}}}{{{\textbf{L}}}_{{\textbf{T}}{\textbf{C}}}}\nonumber \\ {}{} & {} \quad -\rho \frac{{{\textbf{L}}}_{{\textbf{T}}{\textbf{C}}}}{{{\textbf{I}}}_{{\textbf{T}}{\textbf{C}}}}-\varsigma _{3}\frac{{{\textbf{I}}}_{{\textbf{T}}}}{{{\textbf{I}}}_{{\textbf{T}}{\textbf{C}}}}-\nu \frac{{{\textbf{I}}}_{{\textbf{C}}}}{{{\textbf{I}}}_{{\textbf{T}}{\textbf{C}}}}-\varpi \frac{{{\textbf{L}}}_{{\textbf{T}}}}{{\textbf{R}}}-\varphi _{2}\frac{{{\textbf{E}}}{{\textbf{C}}}}{{\textbf{R}}}-\delta \frac{{{\textbf{I}}}_{{\textbf{T}}}}{{\textbf{R}}}-\varphi _{3}\frac{{{\textbf{I}}}_{{\textbf{C}}}}{{\textbf{R}}}\nonumber \\ {}{} & {} \quad -\frac{{\tilde{\mu }}}{4}\big ({{\textbf{S}}}+{{\textbf{L}}}_{{\textbf{T}}}+{{\textbf{I}}}_{{\textbf{T}}}+{{\textbf{E}}}_{{\textbf{C}}}+{{\textbf{I}}}_{{\textbf{C}}}+{{\textbf{L}}}_{{\textbf{T}}{\textbf{C}}}+{{\textbf{I}}}_{{\textbf{T}}{\textbf{C}}}+{{\textbf{R}}}\big )^{\mu +1}. \end{aligned}$$

Next, we construct the following for a bounded closed set:

68 $$\begin{aligned} {\mathbb {D}}_{\epsilon }{} & {} =\Big \{\Lambda \in {\mathbb {R}}_{+}^{8}:~{\textbf{S}}\in [\epsilon ,1/\epsilon ],~{{\textbf{L}}}_{{\textbf{T}}}\in [\epsilon ^{3},1/\epsilon ^{3}],~{{\textbf{I}}}_{{\textbf{T}}}\in [\epsilon ,1/\epsilon ],~{{\textbf{E}}}_{{\textbf{C}}}\in [\epsilon ^{2},1/\epsilon ^{2}],~{{\textbf{I}}}_{{\textbf{C}}}\in [\epsilon ,1/\epsilon ],\nonumber \\ {}{} & {} \qquad \qquad \qquad ~{{\textbf{L}}}_{{\textbf{T}}{\textbf{C}}}\in [\epsilon ^{4},1/\epsilon ^{4}],~{{\textbf{I}}}_{{\textbf{T}}{\textbf{C}}}\in [\epsilon ^{2},1/\epsilon ^{2}],~{{\textbf{R}}}\in [\epsilon ^{3},1/\epsilon ^{3}]\Big \}, \end{aligned}$$

where $\epsilon$ is a non-negative constant that is small enough to meet the subsequent variants

69 $$\begin{aligned}{} & {} -\frac{\nabla \wedge \psi _{{\textbf{C}}}\wedge \alpha _{1}\wedge \psi _{\xi }\wedge \beta }{\epsilon }+{\mathcal {F}}_{4}\le -1,\nonumber \\ {}{} & {} {\mathbb {M}}\big [\alpha _{1}({\textbf{d}}_{1}+{\textbf{b}}_{1}{\textbf{d}}_{2})+\frac{\alpha _{2}(a_{2}{\textbf{d}}_{1}+{\textbf{d}}_{2})(\beta +\varsigma _{3}+\zeta _{{\textbf{T}}}+\delta )}{\theta _{2}\xi }\big ]\epsilon \le 1,\nonumber \\ {}{} & {} -{\tilde{\mu }}+8{\mathcal {F}}_{4}\epsilon ^{\mu +1}\le -8\epsilon ^{\mu +1},\nonumber \\ {}{} & {} -{\tilde{\mu }}+8{\mathcal {F}}_{4}\epsilon ^{3\mu +3}\le -8\epsilon ^{3\mu +3},\nonumber \\ {}{} & {} -{\tilde{\mu }}+8{\mathcal {F}}_{4}\epsilon ^{2\mu +2}\le -8\epsilon ^{2\mu +2},\nonumber \\ {}{} & {} -{\tilde{\mu }}+8{\mathcal {F}}_{4}\epsilon ^{4\mu +4}\le -8\epsilon ^{4\mu +4}, \end{aligned}$$

having

70 $$\begin{aligned} {\mathcal {F}}_{4}=\sup \limits _{{{\textbf{I}}}_{{\textbf{T}}}\in {\mathbb {R}}^{+}}\Big \{{\mathbb {M}}\big [\alpha _{1}({\textbf{d}}_{1}+{\textbf{b}}_{1}{\textbf{d}}_{2})+\frac{\alpha _{2}(a_{2}{\textbf{d}}_{1}+{\textbf{d}}_{2})(\beta +\varsigma _{3}+\zeta _{{\textbf{T}}}+\delta )}{\theta _{2}\xi }\big ]{{\textbf{I}}}_{{\textbf{T}}}-\frac{{\tilde{\mu }}}{8}{{\textbf{I}}}_{{\textbf{T}}}^{\mu +1}\Big \}. \end{aligned}$$

For the sake of simplicity, we can split ${\mathbb {R}}_{+}^{8}\setminus {\mathbb {D}}_{\epsilon }$ into the subsequent sixteen regions:

71 $$\begin{aligned}{} & {} {\mathbb {D}}_{\epsilon }^{1}=\big \{\Lambda \in {\mathbb {R}}_{+}^{8}:~{{\textbf{S}}}\in (0,\epsilon ]\big \},~~~{\mathbb {D}}_{\epsilon }^{2}=\big \{\Lambda \in {\mathbb {R}}_{+}^{8}:~{{\textbf{L}}}_{{\textbf{T}}}\in (0,\epsilon ]\big \},\nonumber \\ {}{} & {} {\mathbb {D}}_{\epsilon }^{3}=\big \{\Lambda \in {\mathbb {R}}_{+}^{8}:~{{\textbf{I}}}_{{\textbf{T}}}\in (0,\epsilon ^{3}],~{{\textbf{S}}}>\epsilon ,~{{\textbf{L}}}_{{\textbf{T}}}>\epsilon \big \},~~~{\mathbb {D}}_{\epsilon }^{4}=\big \{\Lambda \in {\mathbb {R}}_{+}^{8}:~{{\textbf{E}}}_{{\textbf{C}}}\in (0,\epsilon ^{2}],~{{\textbf{S}}}>\epsilon \big \},\nonumber \\ {}{} & {} {\mathbb {D}}_{\epsilon }^{5}=\big \{\Lambda \in {\mathbb {R}}_{+}^{8}:~{{\textbf{I}}}_{{\textbf{C}}}\in (0,\epsilon ]\big \},~~~{\mathbb {D}}_{\epsilon }^{6}=\big \{\Lambda \in {\mathbb {R}}_{+}^{8}:~{{\textbf{L}}}_{{\textbf{T}}{\textbf{C}}}\in (0,\epsilon ^{4}],~{{\textbf{E}}}_{{\textbf{C}}}>\epsilon ^{3}\big \},\nonumber \\ {}{} & {} {\mathbb {D}}_{\epsilon }^{7}=\big \{\Lambda \in {\mathbb {R}}_{+}^{8}:~{{\textbf{I}}}_{{\textbf{T}}{\textbf{C}}}\in (0,\epsilon ^{2}],{{\textbf{I}}}_{{\textbf{T}}}>\epsilon \big \},~~~{\mathbb {D}}_{\epsilon }^{8}=\big \{\Lambda \in {\mathbb {R}}_{+}^{8}:~{{\textbf{R}}}\in (0,\epsilon ^{3}],~{{\textbf{R}}}>\epsilon ^{2}\big \},\nonumber \\ {}{} & {} {\mathbb {D}}_{\epsilon }^{9}=\big \{\Lambda \in {\mathbb {R}}_{+}^{8}:~{{\textbf{S}}}\ge 1/\epsilon \big \},~~~{\mathbb {D}}_{\epsilon }^{10}=\big \{\Lambda \in {\mathbb {R}}_{+}^{8}:~{{\textbf{L}}}_{{\textbf{T}}}\ge 1/\epsilon ^{3}\big \},\nonumber \\ {}{} & {} {\mathbb {D}}_{\epsilon }^{11}=\big \{\Lambda \in {\mathbb {R}}_{+}^{8}:~{{\textbf{I}}}_{{\mathbb {T}}}\ge 1/\epsilon \big \},~~~{\mathbb {D}}_{\epsilon }^{12}=\big \{\Lambda \in {\mathbb {R}}_{+}^{8}:~{{\textbf{E}}}_{{\textbf{C}}}\ge 1/\epsilon ^{2}\big \},\nonumber \\ {}{} & {} {\mathbb {D}}_{\epsilon }^{13}=\big \{\Lambda \in {\mathbb {R}}_{+}^{8}:~{{\textbf{I}}}_{{\textbf{C}}}\ge 1/\epsilon \big \},~~~{\mathbb {D}}_{\epsilon }^{14}=\big \{\Lambda \in {\mathbb {R}}_{+}^{8}:~{{\textbf{L}}}_{{\textbf{T}}{\textbf{C}}}\ge 1/\epsilon ^{4}\big \},\nonumber \\ {}{} & {} {\mathbb {D}}_{\epsilon }^{15}=\big \{\Lambda \in {\mathbb {R}}_{+}^{8}:~{{\textbf{I}}}_{{\textbf{T}}{\textbf{C}}}\ge 1/\epsilon ^{2}\big \},~~~{\mathbb {D}}_{\epsilon }^{16}=\big \{\Lambda \in {\mathbb {R}}_{+}^{8}:~{{\textbf{R}}}\ge 1/\epsilon ^{3}\big \}. \end{aligned}$$

Evidently, $${\mathbb {R}}_{+}^{8}\setminus {\mathbb {D}}_{\epsilon }=\bigcup \limits _{\jmath =1}^{16}{\mathbb {D}}_{\epsilon }^{\jmath 
}.$$ Consequently, it is easy to demonstrate that

72 $$\begin{aligned} {\mathcal {L}}\Phi (\Lambda )\le -1~~\forall ~\Lambda \in {\mathbb {R}}_{+}^{8}\setminus {\mathbb {D}}_{\epsilon }. \end{aligned}$$

This verifies assumption $({\mathcal {H}}_{1})$ of Lemma 1.

The diffusion matrix for model (28) is presented as follows:

73 $$\begin{aligned} {\textbf{Q}}=\begin{pmatrix} \wp _{1}^{2}{{\textbf{S}}}^{2}&{}0&{}0&{}0&{}0&{}0&{}0&{}0\\ 0&{}\wp _{2}^{2}{{\textbf{L}}}_{{\textbf{T}}}^{2}&{}0&{}0&{}0&{}0&{}0&{}0\\ 0&{}0&{}\wp _{3}^{2}{{\textbf{I}}}_{{\textbf{T}}}^{2}&{}0&{}0&{}0&{}0&{}0\\ 0&{}0&{}0&{}\wp _{4}^{2}{{\textbf{E}}}_{{\textbf{C}}}^{2}&{}0&{}0&{}0&{}0\\ 0&{}0&{}0&{}0&{}\wp _{5}^{2}{{\textbf{I}}}_{{\textbf{C}}}^{2}&{}0&{}0&{}0\\ 0&{}0&{}0&{}0&{}0&{}\wp _{6}^{2}{{\textbf{L}}}_{{\textbf{T}}{\textbf{C}}}^{2}&{}0&{}0\\ 0&{}0&{}0&{}0&{}0&{}0&{}\wp _{7}^{2}{{\textbf{I}}}_{{\textbf{T}}{\textbf{C}}}^{2}&{}0\\ 0&{}0&{}0&{}0&{}0&{}0&{}0&{}\wp _{8}^{2}{{\textbf{R}}}^{2}\\ \end{pmatrix}. \end{aligned}$$

It is evident that, matrix ${\textbf{Q}}$ is positive definite $\forall ~\Lambda \in {\mathbb {D}}.$ This verifies assumption $({\mathcal {H}}_{1})$ of Lemma 1. Thus, the model (28) has a unique stationary distribution $\pi (.\,)$ and ergodic. This puts the proof to its conclusion. $\square$

#### Remark 2

For system (28), if ${\mathbb {R}}_{0}>1,$ the illness always endures. According to Theorem 8, if ${\mathbb {R}}_{0}^{{\mathbb {S}}}>1$, the sickness will continue in the stochastic framework (28). In particular, in the absence of noise, that is, $\wp _{\kappa }=0,~(\kappa =1,...,8).$ Observe that $\overline{{\textbf{S}}}=\frac{\nabla }{\psi _{{\textbf{T}}}+\psi _{{\textbf{C}}}+\beta }~~~~~\iff ~~~~\overline{{\textbf{S}}}=\frac{\nabla }{\psi _{{\textbf{C}}}+\beta }.$

Next, the equation is $\overline{{\textbf{S}}}={{\textbf{S}}}_{1}^{0}.$ On the same instance, obtaining

74 $$\begin{aligned} {\mathbb {R}}_{0}^{{\mathbb {S}}}=\frac{\alpha _{2}{\mathbb {R}}_{0}^{{\mathbb {T}}}\big ({\mathbb {R}}_{0}^{{\mathbb {T}}}(\zeta _{{\textbf{C}}}+\varphi _{1}+\varphi _{3})-{\mathcal {K}}_{8}/2+\sqrt{{\mathbb {R}}_{0}^{T}\big ({\mathbb {R}}_{0}^{T}{\mathcal {K}}_{8}^{2}+4\mu \alpha _{1}\big )/2\big )}}{\big (\sqrt{{\mathbb {R}}_{0}^{T}\big ({\mathbb {R}}_{0}^{T}{\mathcal {K}}_{8}^{2}+4\mu \alpha _{1}\big )/2}+(\varphi _{1}+\varphi _{3}){\mathbb {R}}_{0}^{{\mathbb {T}}}-{\mathcal {K}}_{8}{\mathbb {R}}_{0}^{{\mathbb {T}}}/2\big )\big (\sqrt{{\mathbb {R}}_{0}^{T}\big ({\mathbb {R}}_{0}^{T}{\mathcal {K}}_{8}^{2}+4\mu \alpha _{1}\big )/2}+(\zeta _{{\textbf{C}}}+\varphi _{3}){\mathbb {R}}_{0}^{{\mathbb {T}}}-{\mathcal {K}}_{8}{\mathbb {R}}_{0}^{{\mathbb {T}}}/2\big )}>1.\nonumber \end{aligned}$$

## Stochastic COVID-19 model without TB infection

By utilizing the identical technique from probabilistic framework (28) to incorporate random perturbation, we obtain the subsequent stochastic model:

75 $$\begin{aligned} {\left\{ \begin{array}{ll} d{{\textbf{S}}}=\big [\nabla -\frac{\alpha _{2}}{{\mathcal {N}}_{1}}({{\textbf{E}}}_{{\textbf{C}}}+{{\textbf{I}}}_{{\textbf{C}}})-\beta {{\textbf{S}}}\big ]d\tau +\wp _{4\jmath -3}{{\textbf{S}}}d{{\mathbb {B}}}_{4\jmath -3}(\tau ),\\ d{{{\textbf{E}}}_{{\textbf{C}}}}=\big [\frac{\alpha _{2}}{{\mathcal {N}}_{1}}({{\textbf{E}}}_{{\textbf{C}}}+{{\textbf{I}}}_{{\textbf{C}}})-(\beta +\varphi _{1}+\varphi _{2}){{\textbf{E}}}_{{\textbf{C}}}\big ]d\tau +\wp _{4\jmath -2}{{\textbf{E}}}_{{\textbf{C}}}d{{\mathbb {B}}}_{4\jmath -2}(\tau ),\\ d{{{\textbf{I}}}_{{\textbf{C}}}}=\big [\varphi _{1}{{\textbf{E}}}_{{\textbf{C}}}-(\beta +\zeta _{{\textbf{C}}}+\varphi _{3}){{\textbf{I}}}_{{\textbf{C}}}\big ]d\tau +\wp _{4\jmath -1}{{\textbf{I}}}_{{\textbf{C}}}d{{\mathbb {B}}}_{4\jmath -1}(\tau ),\\ d{{\textbf{R}}}=\big [\varphi _{2}{{\textbf{E}}}_{{\textbf{C}}}+\varphi _{3}{{\textbf{I}}}_{{\textbf{C}}}-\beta {{\textbf{R}}}\big ]d\tau +\wp _{4\jmath }{{\textbf{R}}}d{{\mathbb {B}}}_{4\jmath }(\tau ). \end{array}\right. } \end{aligned}$$

Then, we state

$$\begin{aligned} {\mathcal {R}}_{\jmath }^{\kappa }=\frac{\nabla \alpha _{2}\beta \vartheta _{3}}{\vartheta _{3}\vartheta _{2}(\vartheta _{4}\vartheta _{1}-\varphi _{2}\varphi _{3})}. \end{aligned}$$

The values of the parameters have similar significance within the system (75). Indicate

76 $$\begin{aligned} \vartheta _{1}=\beta +\frac{\wp _{4\jmath -3}^{2}}{2},~~\vartheta _{2}=(\beta +\varphi _{2}+\varphi _{1})+\frac{\wp _{4\jmath -2}^{2}}{2},~~~\vartheta _{3}=(\beta +\zeta _{{\textbf{C}}}+\varphi _{3})+\frac{\wp _{4\jmath -1}^{2}}{2},~~~\vartheta _{4}=\beta +\frac{\wp _{4\jmath -3}^{2}}{2}. \end{aligned}$$

The two theorems that proceed are derived from “Codynamics model and preliminaries” and “Stochastic configuration of codynamics of TB-COVID-19 model” section using a similar methodology.

### Theorem 9

Suppose there are initial values $\big ({{\textbf{S}}}(0),{{\textbf{E}}}_{{\textbf{C}}}(0),{{\textbf{I}}}_{{\textbf{C}}}(0),{\textbf{R}}(0)\big )\in {\mathbb {R}}_{+}^{4}$ have unique solution $\big ({{\textbf{S}}}(\tau ),{{\textbf{E}}}_{{\textbf{C}}}(\tau ),{{\textbf{I}}}_{{\textbf{C}}}(\tau ),{\textbf{R}}(\tau )\big )\in {\mathbb {R}}_{+}^{4}$ of the model (75) with $\tau >0$ and the solution will exist in ${\mathbb {R}}_{+}^{4}$ having probability 1 (a.s).

### Theorem 10

Suppose that ${\mathcal {R}}_{\jmath }^{\kappa }>1,$ then model (75) possesses the ergodic functionality and yields a unique stationary distribution $\pi (.\,).$

### Probability density function (P.D.F)

In what follows, we present a mathematical principle pertaining to the P.D.F associated with the subsequent probabilistic framework as:

77 $$\begin{aligned} d\Lambda (\tau )={\hat{{\textbf{c}}}}(\Lambda ,\tau )d\tau +{\hat{{\textbf{d}}}}(\Lambda ,\tau )d{\textbf{Q}}(\tau ), \end{aligned}$$

where $\Lambda$ indicates the parameter whilst ${\hat{{\textbf{c}}}}(\Lambda ,\tau ),~{\hat{{\textbf{d}}}}(\Lambda ,\tau )$ are some functions and ${\textbf{Q}}(\tau )$ is the Wiener technique.

#### Lemma 2

(^50^) Suppose there is a mapping ${\tilde{p}}(\Lambda )$ states the P.D.F associated to the formula (77):

$$\begin{aligned} \partial _{\tau }{\tilde{p}}(\Lambda ,\tau \vert {\Lambda _{0}},{\tau }_{0})=-\partial _{\Lambda }\big [{\hat{{\textbf{c}}}}(\Lambda ,\tau ){\tilde{p}}(\Lambda ,\tau \vert {\Lambda _{0}},{\tau }_{0})\big ]+\frac{1}{2}\partial _{\Lambda }^{2}\Big ({\hat{{\textbf{d}}}}(\Lambda ,\tau )^{2}{\tilde{p}}(\Lambda ,\tau \vert {\Lambda _{0}},{\tau }_{0})\Big ). \end{aligned}$$

Following that, we provide the prerequisites required to find the positive definite (P-D) 4D real symmetric matrix.

#### Lemma 3

Assume that there is a 4D real algebraic equation $\Xi _{0}^{2}+{\textbf{Q}}\Upsilon +\Upsilon {\textbf{Q}}^{{\textbf{T}}}=0$ having $\Xi _{0}=diag(1,0,0,0),$ while $\Upsilon$ indicates the real symmetric matrix.

(i) If
  $$\begin{aligned}{\textbf{Q}}=\begin{pmatrix} -\vartheta _{1}&{}\vartheta _{2}&{}-\vartheta _{3}&{}-\vartheta _{4}\\ 1&{}0&{}0&{}0\\ 0&{}1&{}0&{}0\\ 0&{}0&{}1&{}0 \end{pmatrix}, \end{aligned}$$
  containing with $\vartheta _{1}>0,\vartheta _{3}>0,\vartheta _{4}>0$ and $\vartheta _{1}\vartheta _{2}\vartheta _{3}-\vartheta _{3}^{2}-\vartheta _{1}^{2}\vartheta _{4}>0,$ then
  78 $$\begin{aligned} \Upsilon =\begin{pmatrix}\frac{\vartheta _{2}\vartheta _{3}-\vartheta _{1}\vartheta _{4}}{2(\vartheta _{1}\vartheta _{2}\vartheta _{3}-\vartheta _{3}^{2}-\vartheta _{1}^{2}\vartheta _{4})}&{}0&{}-\frac{\vartheta _{3}}{2(\vartheta _{1}\vartheta _{2}\vartheta _{3}-\vartheta _{3}^{2}-\vartheta _{1}^{2}\vartheta _{4})}&{}0\\ 0&{}\frac{\vartheta _{3}}{2(\vartheta _{1}\vartheta _{2}\vartheta _{3}-\vartheta _{3}^{2}-\vartheta _{1}^{2}\vartheta _{4})}&{}0&{}-\frac{\vartheta _{1}}{2(\vartheta _{1}\vartheta _{2}\vartheta _{3}-\vartheta _{3}^{2}-\vartheta _{1}^{2}\vartheta _{4})}\\ -\frac{\vartheta _{3}}{2(\vartheta _{1}\vartheta _{2}\vartheta _{3}-\vartheta _{3}^{2}-\vartheta _{1}^{2}\vartheta _{4})}&{}0&{}\frac{\vartheta _{1}}{2(\vartheta _{1}\vartheta _{2}\vartheta _{3}-\vartheta _{3}^{2}-\vartheta _{1}^{2}\vartheta _{4})}&{}0\\ 0&{}-\frac{\vartheta _{1}}{2(\vartheta _{1}\vartheta _{2}\vartheta _{3}-\vartheta _{3}^{2}-\vartheta _{1}^{2}\vartheta _{4})}&{}0&{}-\frac{\vartheta _{1}\vartheta _{2}-\vartheta _{3}}{2(\vartheta _{1}\vartheta _{2}\vartheta _{3}-\vartheta _{3}^{2}-\vartheta _{1}^{2}\vartheta _{4})} \end{pmatrix} \end{aligned}$$
  is a P-D.
(ii) If
  $$\begin{aligned}{\textbf{Q}}=\begin{pmatrix} -\vartheta _{1}&{}\vartheta _{2}&{}-\vartheta _{3}&{}\vartheta _{4}\\ 1&{}0&{}0&{}0\\ 0&{}1&{}0&{}0\\ 0&{}0&{}1&{}\vartheta _{5} \end{pmatrix}, \end{aligned}$$
  containing $\vartheta _{1}>0,\vartheta _{3}>0$ and $\vartheta _{1}\vartheta _{2}-\vartheta _{3}>0,$ then
  79 $$\begin{aligned} \Upsilon =\begin{pmatrix}\frac{\vartheta _{2}}{2(\vartheta _{1}\vartheta _{2}-\vartheta _{3})}&{}0&{}-\frac{1}{2(\vartheta _{1}\vartheta _{2}-\vartheta _{3})}&{}0\\ 0&{}\frac{1}{2(\vartheta _{1}\vartheta _{2}-\vartheta _{3})}&{}0&{}0\\ -\frac{1}{2(\vartheta _{1}\vartheta _{2}-\vartheta _{3})}&{}0&{}\frac{\vartheta _{1}}{2\vartheta _{3}(\vartheta _{1}\vartheta _{2}-\vartheta _{3})}&{}0\\ 0&{}0&{}0&{}0 \end{pmatrix}, \end{aligned}$$
  is a semi P-D matrix.
(iii) If
  $$\begin{aligned}{\textbf{Q}}=\begin{pmatrix} -\vartheta _{1}&{}\vartheta _{2}&{}\vartheta _{3}&{}\vartheta _{4}\\ 1&{}0&{}0&{}0\\ 0&{}0&{}\vartheta _{5}&{}\vartheta _{6}\\ 0&{}0&{}\vartheta _{7}&{}\vartheta _{8} \end{pmatrix}, \end{aligned}$$
  containing $\vartheta _{1}>0$ and $\vartheta _{2}>0,$ then
  80 $$\begin{aligned} \Upsilon =\begin{pmatrix}(2\vartheta _{1})^{-1}&{}0&{}0&{}0\\ 0&{}(2\vartheta _{1}\vartheta _{2})^{-1}&{}0&{}0\\ 0&{}0&{}0&{}0\\ 0&{}0&{}0&{}0 \end{pmatrix}, \end{aligned}$$
  is a semi P-D matrix.

#### Proof

Indicate the $\ell$-th significant main component of $\Upsilon$ is $\Upsilon ^{(\ell )},$ which is expressed as

(i) Observe that $\vartheta _{1}(\vartheta _{2}\vartheta _{3}-\vartheta _{1}\vartheta _{4})>\vartheta _{3}^{2}>0,$ then
  $$\begin{aligned} \Upsilon ^{(\Bbbk )}={\left\{ \begin{array}{ll} \frac{\vartheta _{2}\vartheta _{3}-\vartheta _{1}\vartheta _{4}}{2(\vartheta _{1}\vartheta _{2}\vartheta _{3}-\vartheta _{3}^{2}-\vartheta _{1}^{2}\vartheta _{4})}>0,~~\Bbbk =1\\ \frac{\vartheta _{3}(\vartheta _{2}\vartheta _{3}-\vartheta _{1}\vartheta _{4})}{4(\vartheta _{1}\vartheta _{2}\vartheta _{3}-\vartheta _{3}^{2}-\vartheta _{1}^{2}\vartheta _{4})^{2}}>0,~~\Bbbk =2\\ \frac{\vartheta _{3}}{8(\vartheta _{1}\vartheta _{2}\vartheta _{3}-\vartheta _{3}^{2}-\vartheta _{1}^{2}\vartheta _{4})^{2}}>0,~~\Bbbk =3\\ \frac{1}{16(\vartheta _{1}\vartheta _{2}\vartheta _{3}-\vartheta _{3}^{2}-\vartheta _{1}^{2}\vartheta _{4})^{2}}>0,~~\Bbbk =4. \end{array}\right. } \end{aligned}$$
  Furthermore, assertions (ii) and (iii) can be obtained in the same way. $\square$

Here, the precise representation of the density function of system (75) at a quasi-equilibrium point will be derived. In relation to analytical importance, it is important to note that the P.D.F can represent the majority of the unpredictable features of a probabilistic process.

Initially, we apply an analogous change to illustrate (75). For this, consider $\zeta _{4\jmath -3}=\ln {{\textbf{S}}},~\zeta _{4\jmath -2}=\ln {{\textbf{E}}}_{{\textbf{C}}},~\zeta _{4\jmath -1}=\ln {{\textbf{I}}}_{{\textbf{C}}}$ and $\zeta _{4\jmath }=\ln {{\textbf{R}}}.$ Thus, system (75)’s corresponding expression is provided by

81 $$\begin{aligned} {\left\{ \begin{array}{ll} d\zeta _{4\jmath -3}=\big [\nabla e^{-(4\jmath -3)}-\alpha _{2}(e^{4\jmath -2}e^{-(4\jmath -3)}-e^{4\jmath -1}e^{-(4\jmath -3)})-\vartheta _{1}\big ]d\tau +\wp _{4\jmath -3}d{{\mathbb {B}}}_{4\jmath -3}(\tau ),\\ d\zeta _{4\jmath -2}=\big [\alpha _{2}(\vartheta _{2}-e^{4\jmath -1}e^{-(4\jmath -2)})-\vartheta _{2}\big ]d\tau +\wp _{4\jmath -2}d{{\mathbb {B}}}_{4\jmath -2}(\tau ),\\ d\zeta _{4\jmath -1}=\big [\varphi _{1} e^{4\jmath -2}e^{-(4\jmath -1)}-\vartheta _{3}\big ]d\tau +\wp _{4\jmath -2}d{{\mathbb {B}}}_{4\jmath -2}(\tau ),\\ d\zeta _{4\jmath }=\big [\varphi _{2} e^{(4\jmath -1)}e^{-4\jmath }+\varphi _{3} e^{(4\jmath -1)}e^{-4\jmath }-\vartheta _{4}\big ]d\tau +\wp _{4\jmath }d{{\mathbb {B}}}_{4\jmath }(\tau ), \end{array}\right. } \end{aligned}$$

When ${\mathcal {R}}_{0}^{\kappa }>1,$ we illustrate a quasi steady state ${\textbf{U}}_{\jmath }^{*}=({{\textbf{S}}}_{\jmath }^{*},{{{\textbf{E}}}_{{\textbf{C}}}}_{\jmath }^{*},{{{\textbf{I}}}_{{\textbf{C}}}}_{\jmath }^{*},{{{\textbf{R}}}}_{\jmath }^{*}),$ where

82 $$\begin{aligned} {{\textbf{S}}}_{\jmath }^{*}=\frac{\vartheta _{2}\vartheta _{3}}{\alpha _{2}},~~{{{\textbf{E}}}_{{\textbf{C}}}}_{\jmath }^{*}=\frac{\vartheta _{3}{{{\textbf{I}}}_{{\textbf{T}}}}_{\jmath }^{*}}{\alpha _{2}},~~{{{\textbf{I}}}_{{\textbf{T}}}}_{\jmath }^{*}=\frac{\vartheta _{2}({\mathcal {R}}_{0}^{\kappa }-1)(\vartheta _{2}\vartheta _{4}-\varphi _{2}\varphi _{3})}{\alpha _{2}\vartheta _{4}\big (\beta +\varphi _{2}+\varphi _{1}+\frac{\wp _{4\jmath -2}^{2}}{2}\big )-\varphi _{2}\varphi _{3}},~~{{\textbf{R}}}_{\jmath }^{*}=\frac{\varphi _{2}{{{\textbf{E}}}_{{\textbf{C}}}}_{\jmath }^{*}+\varphi _{3}{{{\textbf{I}}}_{{\textbf{C}}}}_{\jmath }^{*}}{\vartheta _{4}}. \end{aligned}$$

Assume that ${\textbf{g}}=\zeta _{\ell }-\zeta _{\ell }^{*},~(\ell =1,...,8).$ Thus, system (81) can be expressed as

83 $$\begin{aligned} {\left\{ \begin{array}{ll} d{\textbf{g}}_{4\jmath -3}=(-{\chi _{11}}{\textbf{g}}_{4\jmath -3}+{\chi _{12}}{\textbf{g}}_{4\jmath -2}-{\chi _{13}}{\textbf{g}}_{4\jmath -1}-{\chi _{14}}{\textbf{g}}_{4\jmath })d\tau +\wp _{4\jmath -3}d{{\mathbb {B}}}_{4\jmath -3}(\tau ),\\ d{\textbf{g}}_{4\jmath -2}=({\chi _{22}}{\textbf{g}}_{4\jmath -3}+{\chi _{22}}{\textbf{g}}_{4\jmath -2}-{\chi _{22}}{\textbf{g}}_{4\jmath -1})d\tau +\wp _{4\jmath -2}d{{\mathbb {B}}}_{4\jmath -2}(\tau ),\\ d{\textbf{g}}_{4\jmath -1}=({\chi _{33}}{\textbf{g}}_{4\jmath -2}-{\chi _{33}}{\textbf{g}}_{4\jmath -2})d\tau +\wp _{4\jmath -1}d{{\mathbb {B}}}_{4\jmath -1}(\tau ),\\ d{\textbf{g}}_{4\jmath }=\big ({\chi _{41}}{\textbf{g}}_{4\jmath -3}+{\chi _{42}}{\textbf{g}}_{4\jmath -2}-({\chi _{41}+\chi _{42}}){\textbf{g}}_{4\jmath }\big )d\tau +\wp _{4\jmath }d{{\mathbb {B}}}_{4\jmath }(\tau ), \end{array}\right. } \end{aligned}$$

where $${\chi _{11}}=\frac{\nabla -\alpha _{2}({{{\textbf{E}}}_{{\textbf{C}}}}_{\jmath }^{*}+{{{\textbf{I}}}_{{\textbf{C}}}}_{\jmath }^{*})}{{{\textbf{S}}}_{\jmath }^{*}},~~{\chi _{12}}=\frac{\alpha _{2}{{{\textbf{E}}}_{{\textbf{C}}}}_{\jmath }^{*}}{{{\textbf{S}}}_{\jmath }^{*}},~~{\chi _{13}}=\frac{\alpha _{2}{{{\textbf{I}}}_{{\textbf{C}}}}_{\jmath }^{*}}{{{\textbf{S}}}_{\jmath }^{*}},~~{\chi _{14}}=\frac{\vartheta _{1}}{{{\textbf{S}}}_{\jmath }^{*}},~~{\chi _{22}}=\frac{\alpha _{2}{{{\textbf{E}}}_{{\textbf{C}}}}_{\jmath }^{*}}{{{{\textbf{E}}}_{{\textbf{C}}}}_{\jmath }^{*}},~~{\chi _{33}}=\frac{\varphi _{1}{{{\textbf{E}}}_{{\textbf{C}}}}_{\jmath }^{*}}{{{{\textbf{I}}}_{{\textbf{C}}}}_{\jmath }^{*}},~~{\chi _{41}}=\frac{\varphi _{2}{{{\textbf{E}}}_{{\textbf{C}}}}_{\jmath }^{*}}{{{{\textbf{R}}}}_{\jmath }^{*}},~~{\chi _{42}}=\frac{\varphi _{3}{{{\textbf{I}}}_{{\textbf{C}}}}_{\jmath }^{*}}{{{{\textbf{R}}}}_{\jmath }^{*}}.$$

Furthermore, $${\chi _{11}}=\vartheta _{1}{\chi _{22}}+{\chi _{13}}={\chi _{33}}\vartheta _{2}=\vartheta _{3}$$ and $${\chi _{44}}=\vartheta _{4}.$$

Define $\Psi (\tau )=\big ({\textbf{g}}_{4\jmath -3}(\tau )....{\textbf{g}}_{4\jmath }(\tau )\big )$ and ${\textbf{Q}}(\tau )=\big ({\textbf{Q}}_{4\jmath -3}(\tau )....{\textbf{Q}}_{4\jmath }(\tau )\big ),$ we have

$$\begin{aligned} d\Psi (\tau )={\textbf{Q}}\Psi (\tau )d\tau +\Xi d{\textbf{Q}}(\tau ), \end{aligned}$$

where

84 $$\begin{aligned} {\textbf{Q}}=\begin{pmatrix} -{\chi _{11}}&{}{\chi _{12}}&{}-{\chi _{13}}&{}{\chi _{14}}\\ {\chi _{22}}&{}-{\chi _{22}}&{}\chi _{22}&{}0\\ 0&{}{\chi _{33}}&{}-{\chi _{33}}&{}0\\ {\chi _{41}}&{}{\chi _{42}}&{}0&{}-({\chi _{14}+\chi _{42}}) \end{pmatrix},~~and ~~\Xi =\begin{pmatrix} \wp _{4\jmath -3}&{}0&{}0&{}0\\ 0&{}\wp _{4\jmath -2}&{}0&{}0\\ 0&{}0&{}\wp _{4\jmath -1}&{}0\\ 0&{}0&{}0&{}\wp _{4\jmath } \end{pmatrix}. \end{aligned}$$

Next, we confirm that the real components of each of ${\textbf{Q}}$’s eigenvalues are negative. The characteristic polynomial of ${\textbf{Q}}$ that corresponds to it is ${\chi _{{\textbf{Q}}}}(\upsilon )={{\tilde{a}}}_{4}+{{\tilde{a}}}_{3}\psi _{1}+{\tilde{a}}_{2}\psi _{1}^{2}+{{\tilde{a}}}_{1}\psi _{1}^{3}+\psi _{1}^{4},$ where

85 $$\begin{aligned}{} & {} {\tilde{a}}_{1}={\chi _{11}}+{\chi _{22}}+{\chi _{33}}+{\chi _{41}}+{\chi _{42}}>0,\nonumber \\ {}{} & {} {\tilde{a}}_{2}=({\chi _{11}}-{\chi _{12}}){\chi _{22}}+({\chi _{11}}-{\chi _{14}}){\chi _{41}}+({\chi _{33}}+{\chi _{42}}){\chi _{11}}+({\chi _{22}}+{\chi _{33}})({\chi _{41}}+{\chi _{42}})>0,\nonumber \\ {}{} & {} {\tilde{a}}_{3}=({\chi _{13}}-{\chi _{12}}){\chi _{22}}{\chi _{33}}+({\chi _{11}}-{\chi _{12}}-{\chi _{14}})({\chi _{41}}+{\chi _{42}}){\chi _{22}}+({\chi _{11}}-{\chi _{14}}){\chi _{33}}{\chi _{41}}+{\chi _{11}}{\chi _{33}}{\chi _{42}}>0,\nonumber \\ {}{} & {} {\tilde{a}}_{4}=\big (({\chi _{13}}-{\chi _{12}}-{\chi _{14}})({\chi _{41}}+{\chi _{42}})+{\chi _{14}}{\chi _{41}}\big ){\chi _{22}}{\chi _{33}}. \end{aligned}$$

Following that, if ${\mathcal {R}}_{0}^{\kappa }>1,$ then

86 $$\begin{aligned}{} & {} {\chi _{13}}-{\chi _{12}}=\Big (1-\frac{\alpha _{2}}{\beta +\varphi _{2}+\varphi _{1}+\frac{\wp _{4\jmath -2}^{2}}{2}}\Big )\alpha _{2}{{\textbf{I}}}_{{\textbf{C}}}>0.\nonumber \\ {}{} & {} ({\chi _{13}}-{\chi _{12}}-{\chi _{14}})({\chi _{41}}+{\chi _{42}})+{\chi _{14}}{\chi _{41}}=\Big (\frac{\nabla }{{{\textbf{S}}}^{*}}-\vartheta _{1}\Big )+\varphi _{2}\varphi _{3}=\vartheta _{1}\vartheta _{4}({\mathcal {R}}_{0}^{\kappa }-1)>0.\nonumber \\ {\tilde{a}}_{1}{\tilde{a}}_{2}{} & {} \ge {\chi _{33}}\big [({\chi _{11}}-{\chi _{12}}){\chi _{22}}+({\chi _{11}}-{\chi _{14}}){\chi _{41}}+{\chi _{11}}{\chi _{42}}\big ]+{\chi _{11}}{\chi _{22}}({\chi _{41}}+{\chi _{42}})\ge {\tilde{a}}_{3}+{\tilde{a}}_{1}({\tilde{a}}_{2}{\tilde{a}}_{3}-{\tilde{a}}_{1}{\tilde{a}}_{4})\nonumber \\ {}{} & {} \ge {\tilde{a}}_{1}\big \{\big [({\chi _{11}}({\chi _{41}}+{\chi _{42}}))+{\chi _{22}}({\chi _{11}}-{\chi _{12}}+{\chi _{41}+{\chi _{42}}})-{\chi _{14}}{\chi _{41}}\big ]{\chi _{22}}({\chi _{11}}-{\chi _{12}}-{\chi _{14}})({\chi _{41}}+{\chi _{42}})\nonumber \\ {}{} & {} \quad +\big [{\chi _{11}}({\chi _{22}}+{\chi _{33}}+{\chi _{41}}+{\chi _{42}})+{\chi _{33}}({\chi _{41}}+{\chi _{42}})-{\chi _{12}}{\chi _{22}}-{\chi _{14}}{\chi _{41}}\big ]{\chi _{33}}({\chi _{11}}-{\chi _{14}})({\chi _{41}}+{\chi _{42}})\nonumber \\ {}{} & {} \quad +{\chi _{14}}{\chi _{33}}{\chi _{42}}{\tilde{a}}_{2}\big \} \nonumber \\ {}{} & {} \ge \big [{\chi _{22}}({\chi _{41}}+{\chi _{42}})({\chi _{11}}-{\chi _{12}}-{\chi _{14}})+({\chi _{13}}-{\chi _{12}}){\chi _{22}}{\chi _{33}}+({\chi _{11}}-{\chi _{14}}){\chi _{33}}{\chi _{41}}+{\chi _{11}}{\chi _{33}}{\chi _{42}}\big ]\nonumber \\ {}{} & {} \quad \times {\chi _{22}}({\chi _{11}}-{\chi _{12}}-{\chi _{14}})({\chi _{41}}+{\chi _{42}})+\big [({\chi _{11}}-{\chi _{12}}-{\chi _{14}})({\chi _{41}}+{\chi _{42}}){\chi _{22}}+(\chi _{13}-{\chi _{12}}){\chi _{22}}{\chi _{33}}\nonumber \\ {}{} & {} \quad +({\chi _{11}}-{\chi _{14}}){\chi _{33}}{\chi _{41}}+{\chi _{11}}{\chi _{33}}{\chi _{42}}\big ](\chi _{13}-{\chi _{12}}){\chi _{22}}{\chi _{33}}+\big [({\chi _{11}}-{\chi _{12}}-{\chi _{14}})({\chi _{41}}+{\chi _{42}}){\chi _{22}}\nonumber \\ {}{} & {} \quad +({\chi _{13}}-{\chi _{12}}){\chi _{22}}{\chi _{33}}+(\chi _{11}-{\chi _{41}}){\chi _{33}}{\chi _{41}}+{\chi _{11}}{\chi _{33}}{\chi _{42}}\big ]{\chi _{33}}({\chi _{11}}-{\chi _{14}})({\chi _{41}}+{\chi _{42}})+{\chi _{14}}{\chi _{33}}{\chi _{42}}{\tilde{a}}_{1}{\tilde{a}}_{2}\nonumber \\ {}{} & {} >{\tilde{a}}_{3}^{2}. \end{aligned}$$

Thus, ${\tilde{a}}_{\jmath }>0,~(\jmath =1,...,4)~({\tilde{a}}_{1}{\tilde{a}}_{2}-{\tilde{a}}_{3})>0$ and ${\tilde{a}}_{1}{\tilde{a}}_{2}{\tilde{a}}_{3}-{\tilde{a}}_{3}^{2}-{\tilde{a}}_{1}^{2}{\tilde{a}}_{4}>0.$ Subsequently it appears that A possesses every negative real-part eigenvalues that correspond to the Routh-Hurwitz stability condition^51^.

With reference to Lemma 2, the Fokker–Planck equation below is satisfied by the relevant P.D.F ${\mathcal {U}}(\Psi )$ to the Quasi-stationary condition of the system (81) can be expressed as

$$\begin{aligned}{} & {} \sum \limits _{\jmath =1}^{2}\Big (\frac{\wp _{4\jmath -3}^{2}}{2}\frac{\partial ^{2}{\mathcal {U}}}{\partial {\textbf{g}}_{4\jmath -3}^{2}}+\frac{\wp _{4\jmath -2}^{2}}{2}\frac{\partial ^{2}{\mathcal {U}}}{\partial {\textbf{g}}_{4\jmath -2}^{2}}+\frac{\wp _{4\jmath -1}^{2}}{2}\frac{\partial ^{2}{\mathcal {U}}}{\partial {\textbf{g}}_{4\jmath -1}^{2}}+\frac{\wp _{4\jmath }^{2}}{2}\frac{\partial ^{2}{\mathcal {U}}}{\partial {\textbf{g}}_{4\jmath }^{2}}\Big )\nonumber \\ {}{} & {} =\sum \limits _{\jmath =1}^{2}\Big \{\frac{\partial }{\partial {\textbf{g}}_{4\jmath -3}}\big ({\chi _{14}}{\textbf{g}}_{4\jmath }-{\chi _{13}}{\textbf{g}}_{4\jmath -1}-{\chi _{11}}{\textbf{g}}_{4\jmath -3}\big ){\mathcal {U}}+\frac{\partial }{\partial {\textbf{g}}_{4\jmath -2}}\big ({\chi _{22}}{\textbf{g}}_{4\jmath -3}-{\chi _{22}}{\textbf{g}}_{4\jmath -2}+{\chi _{22}}{\textbf{g}}_{4\jmath -1}\big ){\mathcal {U}}\nonumber \\ {}{} & {} \quad +\frac{\partial }{\partial {\textbf{g}}_{4\jmath -1}}\big ({\chi _{33}}{\textbf{g}}_{4\jmath -2}-{\chi _{33}}{\textbf{g}}_{4\jmath -2}\big ){\mathcal {U}}+\frac{\partial }{\partial {\textbf{g}}_{4\jmath }}\big ({\chi _{41}}{\textbf{g}}_{4\jmath -3}+{\chi _{42}}{\textbf{g}}_{4\jmath -2}-({\chi _{41}}+{\chi _{42}}){\textbf{g}}_{4\jmath }\big ){\mathcal {U}}\Big \}. \end{aligned}$$

Given that $\Xi$ is an invariant matrix, one can determine that ${\mathcal {U}}(\Psi )$ is potentially identified as having a Gaussian distribution by incorporating the pertinent findings of Roozen^52^:

$$\begin{aligned} {\mathcal {U}}(\Psi )={\tilde{c}}\exp \Big (\frac{-1}{2}\Psi ^{{\textbf{T}}}{\mathbb {M}}\Psi \Big ), \end{aligned}$$

where ${\tilde{c}}$ justifying $\int \limits _{{\mathbb {R}}_{+}^{4}}{\tilde{c}}\exp \Big (\frac{-1}{2}\Psi ^{{\textbf{T}}}{\mathbb {M}}\Psi \Big )d\Psi =1$ and ${\mathbb {M}}=({\theta _{1}}_{\jmath \kappa })_{4\times 4}$ is a real symmetric matrix fulfilling

87 $$\begin{aligned} {\mathbb {M}}\Xi ^{2}{\mathbb {M}}+{\mathbb {M}}{\textbf{Q}}+{\textbf{Q}}^{{\textbf{T}}}{\mathbb {M}}=0.\end{aligned}$$

If $M^{-1}$ holds, we indicate $\Pi =M^{-1},$ ${\mathbb {M}}$ can be found to possess the equivalent degree of positive definiteness. Following this, (87) has the structure that follows.

88 $$\begin{aligned} \Xi ^{2}+{\textbf{Q}}\Pi +\Pi {\textbf{Q}}^{{\textbf{T}}}=0.\end{aligned}$$

The mathematical structure of (88) is obtained by employing a finitely autonomous coherence theory, which gives us $\Xi =\sum \limits _{\ell =1}^{4}\Xi _{\ell }$ and $\Pi =\sum \limits _{\ell =1}^{4},$ then we have

$$\begin{aligned} \Xi _{\ell }^{2}+{\textbf{Q}}\Pi _{\ell }+\Pi _{\ell }{\textbf{Q}}^{{\textbf{T}}}=0,~~\ell =1,...,4, \end{aligned}$$

where

$$\begin{aligned} \Xi _{1}=\begin{pmatrix} \wp _{4\jmath -3}^{2}&{}0&{}0&{}0\\ 0&{}0&{}0&{}0\\ 0&{}0&{}0&{}0\\ 0&{}0&{}0&{}0\\ \end{pmatrix},~~ \Xi _{2}=\begin{pmatrix} 0&{}0&{}0&{}0\\ 0&{}\wp _{4\jmath -2}^{2}&{}0&{}0\\ 0&{}0&{}0&{}0\\ 0&{}0&{}0&{}0\\ \end{pmatrix},~~\Xi _{3}=\begin{pmatrix} 0&{}0&{}0&{}0\\ 0&{}0&{}0&{}0\\ 0&{}0&{}\wp _{4\jmath -1}^{2}&{}0\\ 0&{}0&{}0&{}0\\ \end{pmatrix},~~\Xi _{4}=\begin{pmatrix} 0&{}0&{}0&{}0\\ 0&{}0&{}0&{}0\\ 0&{}0&{}0&{}0\\ 0&{}0&{}0&{}\wp _{4\jmath }^{2}\\ \end{pmatrix}, \end{aligned}$$

and $\Pi _{\ell }$ are decided upon thereafter.

Taking into account ${\textbf{g}}_{\ell }=\upsilon _{\ell }-\upsilon _{\ell }^{*}$ and the transformation between the frameworks (75) and (81) yields the following:

$$\begin{aligned} {\mathcal {U}}({\tilde{\Psi }})=\frac{1}{4\varphi _{2}^{2}}\vert \Pi \vert ^{-1/2}\exp \Big (\frac{-1}{2}{\tilde{\Psi }}\Pi ^{-1}{\tilde{\Psi }}^{{\textbf{T}}}\Big ), \end{aligned}$$

where $${\tilde{\Psi }}=\Big (\ln \frac{{\textbf{S}}}{{{\textbf{S}}}^{*}},\ln \frac{{{\textbf{E}}}_{{\textbf{C}}}}{{{\textbf{E}}}_{{\textbf{C}}}^{*}},\ln \frac{{{\textbf{I}}}_{{\textbf{C}}}}{{{\textbf{I}}}_{{\textbf{C}}}^{*}},\ln \frac{{{\textbf{R}}}}{{{\textbf{R}}}^{*}}\Big ).$$

#### Theorem 11

Surmising that ${\mathcal {R}}_{0}^{\kappa }>1,$ for any $\big ({{\textbf{S}}}(0),{{\textbf{E}}}_{{\textbf{C}}}(0),{{\textbf{I}}}_{{\textbf{C}}}(0),{{\textbf{R}}}(0)\big )\in {\mathbb {R}}_{+}^{4},$ then the solution $\big ({{\textbf{S}}}(\tau ),{{\textbf{E}}}_{{\textbf{C}}}(\tau ),{{\textbf{I}}}_{{\textbf{C}}}(\tau ),{{\textbf{R}}}(\tau )\big )\in {\mathbb {R}}_{+}^{4}$ model (75) possess a log-normal P.D.F ${\mathcal {U}}({\tilde{\Psi }})$ about ${\textbf{U}}_{\jmath }^{*}$ as follows ${\tilde{\Psi }}=\Big (\ln \frac{{\textbf{S}}}{{{\textbf{S}}}^{*}},\ln \frac{{{\textbf{E}}}_{{\textbf{C}}}}{{{\textbf{E}}}_{{\textbf{C}}}^{*}},\ln \frac{{{\textbf{I}}}_{{\textbf{C}}}}{{{\textbf{I}}}_{{\textbf{C}}}^{*}},\ln \frac{{{\textbf{R}}}}{{{\textbf{R}}}^{*}}\Big )$ having $\Pi =\Pi _{\ell },~(\ell =1,...,4)$ is a positive definite matrix and the components $\Pi _{1},\Pi _{2},\Pi _{3}$ and $\Pi _{4}$ are described as

89 $$\begin{aligned}{} & {} \Pi _{1}={\left\{ \begin{array}{ll} ({\chi _{22}}{\chi _{33}}{\chi _{41}}\wp _{4\jmath -3})^{2}({\textbf{U}}_{1}{\textbf{H}}_{1})^{-1}\Upsilon _{1}\big [({\textbf{U}}_{1}{\textbf{H}}_{1})^{-1}\big ]^{{\textbf{T}}},~~~~if~~~~\varpi _{1}=0,\\ ({\chi _{22}}{\chi _{33}}\wp _{4\jmath -3})^{2}({\textbf{U}}_{2}{\textbf{H}}_{2}{\textbf{H}}_{1})^{-1}\Upsilon _{2}\big [({\textbf{U}}_{2}{\textbf{H}}_{2}{\textbf{H}}_{1})^{-1}\big ]^{{\textbf{T}}},~~~~if~~~~\varpi _{1}\ne 0,~\varpi _{2}=0,\\ ({\chi _{22}}{\chi _{33}}\varpi _{2}\wp _{4\jmath -3})^{2}({\textbf{U}}_{3}{\textbf{H}}_{2}{\textbf{H}}_{1})^{-1}\Upsilon _{1}\big [({\textbf{U}}_{3}{\textbf{H}}_{2}{\textbf{H}}_{1})^{-1}\big ]^{{\textbf{T}}},~~~~if~~~~\varpi _{1}\ne 0,~\varpi _{2}\ne 0,\\ \end{array}\right. }\nonumber \\ {}{} & {} \Pi _{2}={\left\{ \begin{array}{ll} ({\chi _{12}}\wp _{4\jmath -2})^{2}({\textbf{U}}_{4}{\textbf{H}}_{3})^{-1}\Upsilon _{3}\big [({\textbf{U}}_{4}{\textbf{H}}_{3})^{-1}\big ]^{{\textbf{T}}},~~~~if~~~~\varpi _{3}=0,~\varpi _{4}=0,\\ ({\chi _{14}}{\chi _{33}}\varpi _{3}\wp _{4\jmath -2})^{2}({\textbf{U}}_{5}{\textbf{H}}_{3})^{-1}\Upsilon _{1}\big [({\textbf{U}}_{5}{\textbf{H}}_{3})^{-1}\big ]^{{\textbf{T}}},~~~~if~~~~\varpi _{3}\ne 0,~\varpi _{4}=0,\\ ({\chi _{13}}{\chi _{42}}\varpi _{4}\wp _{4\jmath -2})^{2}({\textbf{U}}_{6}{\textbf{H}}_{4}{\textbf{H}}_{3})^{-1}\Upsilon _{1}\big [({\textbf{U}}_{6}{\textbf{H}}_{4}{\textbf{H}}_{3})^{-1}\big ]^{{\textbf{T}}},~~~~if~~~~\varpi _{3}=0,~\varpi _{4}\ne 0, ~\varpi _{3}\ne 0,~\varpi _{4}=0,\\ ({\chi _{12}}\varpi _{4}\wp _{4\jmath -2})^{2}({\textbf{U}}_{7}{\textbf{H}}_{5}{\textbf{H}}_{3})^{-1}\Upsilon _{4}\big [({\textbf{U}}_{7}{\textbf{H}}_{5}{\textbf{H}}_{3})^{-1}\big ]^{{\textbf{T}}},~~~~if~~~~\varpi _{3}\ne 0,~\varpi _{4}\ne 0,\varpi _{5}=0,\\ ({\chi _{12}}\varpi _{4}\varpi _{5}\wp _{4\jmath -2})^{2}({\textbf{U}}_{8}{\textbf{H}}_{5}{\textbf{H}}_{3})^{-1}\Upsilon _{1}\big [({\textbf{U}}_{8}{\textbf{H}}_{5}{\textbf{H}}_{3})^{-1}\big ]^{{\textbf{T}}},~~~~if~~~~\varpi _{3}\ne 0,~\varpi _{4}\ne 0,\varpi _{5}\ne 0,\\ \end{array}\right. }\nonumber \\ {}{} & {} \Pi _{3}={\left\{ \begin{array}{ll} (\chi _{13}\wp _{4\jmath -1})^{2}({\textbf{U}}_{9}{\textbf{H}}_{6})^{-1}\Upsilon _{5}\big [({\textbf{U}}_{9}{\textbf{H}}_{6})^{-1}\big ]^{{\textbf{T}}}~~~~if~~~\varpi _{6}=0,\\ (\chi _{13}{\chi _{42}}\varpi _{6}\wp _{4\jmath -1})^{2}({\textbf{U}}_{10}{\textbf{H}}_{6})^{-1}\Upsilon _{1}\big [({\textbf{U}}_{10}{\textbf{H}}_{6})^{-1}\big ]^{{\textbf{T}}}~~~~if~~~\varpi _{6}\ne 0,\\ \end{array}\right. }\nonumber \\ {}{} & {} \Pi _{4}=(\chi _{14}{\chi _{22}}{\chi _{33}}\wp _{4\jmath })^{2}({\textbf{U}}_{11}{\textbf{H}}_{7})^{-1}\Upsilon _{1}\bigg [({\textbf{U}}_{11}{\textbf{H}}_{7})^{-1}\bigg ]^{{\textbf{T}}}, \end{aligned}$$

where

90 $$\begin{aligned} \varpi _{1}{} & {} =({\chi _{22}}-{\chi _{41}})(\chi _{41}+{\chi _{42}})/{\chi _{22}},\nonumber \\\varpi _{2}{} & {} =\varpi _{1}-{\chi _{41}-(\chi _{41}+{\chi _{42}})}\varpi _{1}/{\chi _{33}},\nonumber \\ \varpi _{3}{} & {} =\big ({\chi _{41}}{\chi _{12}}^{2}-{\chi _{14}}{\chi _{42}}^{2}+{\chi _{11}}{\chi _{12}{\chi _{42}}}+{\chi _{31}}{\chi _{33}}{\chi _{42}}-{\chi _{12}}{\chi _{42}}(\chi _{41}+{\chi _{42}})\big )/{\chi _{12}^{2}},\nonumber \\\varpi _{4}{} & {} ={\chi _{33}}({\chi _{11}}{\chi _{12}}-{\chi _{12}}{\chi _{33}}+{\chi _{13}}{\chi _{33}}-{\chi _{14}}{\chi _{42}})/{\chi _{12}}^{2},\nonumber \\ \varpi _{5}{} & {} =-{\chi _{14}}{\chi _{33}}/{\chi _{12}}+\varpi _{4}\lambda _{3}/\varpi _{3}-{\chi _{13}}{\chi _{42}}\varpi _{4}^{2}/{\chi _{12}}\varpi _{3}^{2},\nonumber \\ \varpi _{6}{} & {} =({\chi _{13}}^{2}-{\chi _{12}}{\chi _{22}}-{\chi _{11}}{\chi _{13}}+{\chi _{13}}{\chi _{22}}){\chi _{22}}/{\chi _{13}}^{2},\nonumber \\ \lambda _{3}{} & {} =({\chi _{13}}-{\chi _{12}}){\chi _{22}}{\chi _{33}}-{\chi _{14}}{\chi _{33}}{\chi _{41}}+{\chi _{14}}({{\chi _{41}}}-{\chi _{22}})({\chi _{41}}+{\chi _{42}}),\end{aligned}$$

and the matrices ${\textbf{U}}_{\varsigma _{1}},~(\varsigma _{1}-1,...,11),~{\textbf{H}}_{\varsigma _{2}},~(\varsigma _{2}=1,...,7)$ and $\varpi _{{\textbf{s}}},~({\textbf{s}}=1,...,5)$ are illustrated in the subsequent result.

#### Proof

*Case A*:  Considering

91 $$\begin{aligned} \Xi _{1}^{2}+{\textbf{Q}}\Pi _{1}+\Pi _{1}{\textbf{Q}}^{{\textbf{T}}}=0. \end{aligned}$$

In view of the elimination matrix ${\textbf{H}}_{1}$ as

$$\begin{aligned} {\textbf{H}}_{1}=\begin{pmatrix} 1&{}00&{}0\\ 0&{}1&{}0&{}0\\ 0&{}0&{}1&{}0\\ 0&{}-{\chi _{41}}/{\chi _{22}}&{}0&{}1 \end{pmatrix}. \end{aligned}$$

Consequently, we get

$$\begin{aligned} {\textbf{Q}}_{1}={\textbf{H}}_{1}{\textbf{Q}}{\textbf{H}}_{1}^{-1}=\begin{pmatrix} -{\chi _{11}}&{}{\chi _{12}}{\chi _{22}}+{\chi _{41}}{\chi _{41}}/{\chi _{22}}&{}-{\chi _{13}}&{}{\chi _{14}}\\ {\chi _{22}}&{}-{\chi _{22}}&{}{\chi _{22}}&{}0\\ 0&{}{\chi _{33}}&{}-{\chi _{33}}&{}0\\ 0&{}\varpi _{1}&{}-{\chi _{41}}&{}-({\chi _{41}+{\chi _{42}}}) \end{pmatrix}, \end{aligned}$$

where $$\varpi _{1}=({\chi _{22}}-{\chi _{41}})({\chi _{41}}+{\chi _{42}})/{\chi _{22}}.$$

The subsequent sub-stages are then taken out of the appropriate evaluation.

*Subphase AI* Choose $\varpi _{1}=1$ and ${\mathcal {N}}=(0,0,0,1),$ then there is ${\textbf{U}}_{1}{\textbf{Q}}_{1}{\textbf{U}}_{1}^{-1}={\textbf{Q}}_{1},$ where ${\textbf{U}}_{1}=\big ({\mathcal {N}}{\textbf{Q}}_{1}^{3},{\mathcal {N}}{\textbf{Q}}_{1}^{2},{\mathcal {N}}{\textbf{Q}}_{1},{\mathcal {N}}\big )^{{\textbf{T}}}$ and

$$\begin{aligned} {\textbf{Q}}_{1}=\begin{pmatrix} -\tilde{a_{1}}&{}-\tilde{a_{2}}&{}-\tilde{a_{3}}&{}-\tilde{a_{4}}\\ 1&{}0&{}0&{}0\\ 0&{}1&{}0&{}0\\ 0&{}0&{}1&{}0 \end{pmatrix}. \end{aligned}$$

Consequently, it is possible to write the appropriate formula of (91) as

$$\begin{aligned} ({\textbf{U}}_{1}{\textbf{H}}_{1})\Xi _{1}^{2}({\textbf{U}}_{1}{\textbf{H}}_{1})^{{\textbf{T}}}+{\textbf{Q}}_{1}\big (({\textbf{U}}_{1}{\textbf{H}}_{1})\Pi _{1}({\textbf{U}}_{1}{\textbf{H}}_{1})^{{\textbf{T}}}\big )+\big (({\textbf{U}}_{1}{\textbf{H}}_{1})\Pi _{1}({\textbf{U}}_{1}{\textbf{H}}_{1})^{{\textbf{T}}}\big ){\textbf{Q}}_{1}^{{\textbf{T}}}=0. \end{aligned}$$

By making the use of Lemma 3, we determine $({\textbf{U}}_{1}{\textbf{H}}_{1})\Pi _{1}({\textbf{U}}_{1}{\textbf{H}}_{1})^{{\textbf{T}}}=({\chi _{22}}{\chi _{33}}{\chi _{41}}\wp _{4\jmath -3})^{2}\Upsilon _{1},$ where

$$\begin{aligned} \Upsilon _{1}=\begin{pmatrix} \frac{{\tilde{a}}_{2}{\tilde{a}}_{3}-{\tilde{a}}_{1}{\tilde{a}}_{4}}{2({\tilde{a}}_{1}{\tilde{a}}_{2}{\tilde{a}}_{3}-{\tilde{a}}_{3}^{2}-{\tilde{a}}_{1}^{2}{\tilde{a}}_{4})}&{}0&{}-\frac{{\tilde{a}}_{3}}{2({\tilde{a}}_{1}{\tilde{a}}_{2}{\tilde{a}}_{3}-{\tilde{a}}_{3}^{2}-{\tilde{a}}_{1}^{2}{\tilde{a}}_{4})}&{}0\\ 0&{}\frac{{\tilde{a}}_{3}}{2({\tilde{a}}_{1}{\tilde{a}}_{2}{\tilde{a}}_{3}-{\tilde{a}}_{3}^{2}-{\tilde{a}}_{1}^{2}{\tilde{a}}_{4})}&{}0&{}-\frac{{\tilde{a}}_{1}}{2({\tilde{a}}_{1}{\tilde{a}}_{2}{\tilde{a}}_{3}-{\tilde{a}}_{3}^{2}-{\tilde{a}}_{1}^{2}{\tilde{a}}_{4})}\\ -\frac{{\tilde{a}}_{3}}{2({\tilde{a}}_{1}{\tilde{a}}_{2}{\tilde{a}}_{3}-{\tilde{a}}_{3}^{2}-{\tilde{a}}_{1}^{2}{\tilde{a}}_{4})}&{}0&{}\frac{{\tilde{a}}_{31}}{2({\tilde{a}}_{1}{\tilde{a}}_{2}{\tilde{a}}_{3}-{\tilde{a}}_{3}^{2}-{\tilde{a}}_{1}^{2}{\tilde{a}}_{4})}&{}0\\ 0&{}-\frac{{\tilde{a}}_{1}}{2({\tilde{a}}_{1}{\tilde{a}}_{2}{\tilde{a}}_{3}-{\tilde{a}}_{3}^{2}-{\tilde{a}}_{1}^{2}{\tilde{a}}_{4})}&{}0&{}-\frac{{\tilde{a}}_{1}{\tilde{a}}_{2}-{\tilde{a}}_{3}}{2({\tilde{a}}_{1}{\tilde{a}}_{2}{\tilde{a}}_{3}-{\tilde{a}}_{3}^{2}-{\tilde{a}}_{1}^{2}{\tilde{a}}_{4})} \end{pmatrix}, \end{aligned}$$

is a P-D symmetric matrix. Therefore, $\Pi _{1}=({\chi _{22}}{\chi _{33}}{\chi _{41}}\wp _{4\jmath -3})^{2}({\textbf{U}}_{1}{\textbf{H}}_{1})^{-1}\Upsilon _{1}\big (({\textbf{U}}_{1}{\textbf{H}}_{1})^{-1}\big )^{{\textbf{T}}}$ is also a P-D matrix.

*Subcase AII* Taking $\varpi _{1}\ne 0$ and also suppose that ${\textbf{Q}}_{2}={\textbf{H}}_{2}{\textbf{Q}}_{1}{\textbf{H}}_{2}^{-1},$ where

$$\begin{aligned} {\textbf{H}}_{2}=\begin{pmatrix} 1&{}0&{}0&{}0\\ 0&{}1&{}0&{}0\\ 0&{}0&{}1&{}0\\ 0&{}0&{}-\frac{\varpi _{1}}{{\chi _{33}}}&{}1 \end{pmatrix},~~and~~~{\textbf{Q}}_{2}={\textbf{H}}_{2}{\textbf{Q}}_{1}{\textbf{H}}_{2}^{-1}=\begin{pmatrix} -{\chi _{11}}&{}\frac{{\chi _{12}}{\chi _{22}}+{\chi _{14}}{\chi _{41}}}{{\chi _{22}}}&{}\frac{{\chi _{14}}{\chi _{41}}-{\chi _{13}}{\chi _{22}}{\chi _{33}}}{{\chi _{22}}{\chi _{33}}}&{}{\chi _{14}}\\ {\chi _{22}}&{}-{\chi _{22}}&{}{\chi _{22}}&{}0\\ 0&{}{\chi _{33}}&{}-{\chi _{33}}&{}0\\ 0&{}0&{}\varpi _{2}&{}-({\chi _{41}}+\chi _{42}) \end{pmatrix}, \end{aligned}$$

containing $$\varpi _{2}=\varpi _{1}-{\chi _{41}}-({\chi _{41}}+{\chi _{42}})\varpi _{1}/{\chi _{33}}.$$

*Subcase AIII* Taking $\varpi _{1}\ne 0$ and $\varpi _{2}=0.$ Moreover, suppose that ${\textbf{Q}}_{2}={\textbf{U}}_{2}{\textbf{Q}}_{2}{\textbf{U}}_{2}^{-1},$ where

$$\begin{aligned} {\textbf{U}}_{2}=\begin{pmatrix} {\chi _{22}}{\chi _{33}}&{}-{\chi _{33}}({\chi _{22}}+{\chi _{33}})&{}{\chi _{33}}^{2}+{\chi _{22}}{\chi _{33}}&{}0\\ 0&{}{\chi _{33}}&{}-{\chi _{33}}&{}0\\ 0&{}0&{}1&{}0\\ 0&{}0&{}0&{}1 \end{pmatrix},~~and~~~{\textbf{Q}}_{2}=\begin{pmatrix} -\lambda _{1}&{}-\lambda _{2}&{}-\lambda _{3}&{}{\chi _{14}}{\chi _{22}}{\chi _{33}}\\ 1&{}0&{}0&{}0\\ 0&{}1&{}0&{}0\\ 0&{}0&{}0&{}-({\chi _{41}}+\chi _{42}) \end{pmatrix}, \end{aligned}$$

containing $\lambda _{2}={\chi _{11}}+{\chi _{22}}+{\chi _{33}}>0,~\lambda _{2}=({\chi _{11}}-{\chi _{12}}){\chi _{22}}+{\chi _{11}}{\chi _{33}}-{\chi _{14}}{\chi _{41}}>0$ and $\lambda _{3}=({\chi _{13}}-{\chi _{12}}){\chi _{22}}{\chi _{33}}-{\chi _{14}}{\chi _{33}}{\chi _{41}}+{\chi _{14}}({\chi _{41}}-{\chi _{22}})({\chi _{41}}+{\chi _{42}})>0.$ Hence, we get

$$\begin{aligned} ({\textbf{U}}_{2}{\textbf{H}}_{2}{\textbf{H}}_{1})\Xi _{1}^{2}({\textbf{U}}_{2}{\textbf{H}}_{2}{\textbf{H}}_{1})^{{\textbf{T}}}+{\textbf{Q}}_{2}\big (({\textbf{U}}_{2}{\textbf{H}}_{2}{\textbf{H}}_{1})\Pi _{1}({\textbf{U}}_{2}{\textbf{H}}_{2}{\textbf{H}}_{1})^{{\textbf{T}}}\big )+\big (({\textbf{U}}_{2}{\textbf{H}}_{2}{\textbf{H}}_{1})\Pi _{1}({\textbf{U}}_{2}{\textbf{H}}_{2}{\textbf{H}}_{1})^{{\textbf{T}}}\big ){\textbf{Q}}_{2}^{{\textbf{T}}}=0. \end{aligned}$$

By making the use of Lemma 3, we determine $({\textbf{U}}_{2}{\textbf{H}}_{2}{\textbf{H}}_{1})\Pi _{1}({\textbf{U}}_{2}{\textbf{H}}_{2}{\textbf{H}}_{1})^{{\textbf{T}}}=({\chi _{22}}{\chi _{33}}{\chi _{41}}\wp _{4\jmath -3})^{2}\Upsilon _{2},$ where

$$\begin{aligned} \Upsilon _{2}=\begin{pmatrix} \frac{\lambda _{2}}{2(\lambda _{1}\lambda _{2}-\lambda _{3})}&{}0&{}-\frac{1}{2(\lambda _{1}\lambda _{2}-\lambda _{3})}&{}0\\ 0&{}\frac{1}{2(\lambda _{1}\lambda _{2}-\lambda _{3})}&{}0&{}0\\ -\frac{1}{2(\lambda _{1}\lambda _{2}-\lambda _{3})}&{}0&{}\frac{\lambda _{1}}{2(\lambda _{1}\lambda _{2}-\lambda _{3})}&{}0\\ 0&{}0&{}0&{}0 \end{pmatrix}, \end{aligned}$$

is a symmetric, semi P-D matrix. Thus, $\Pi _{1}=({\chi _{22}}{\chi _{33}}\wp _{4\jmath -3})^{2}({\textbf{U}}_{2}{\textbf{H}}_{2}{\textbf{H}}_{1})^{-1}\Upsilon _{2}\big (({\textbf{U}}_{2}{\textbf{H}}_{2}{\textbf{H}}_{1})^{-1}\big )^{{\textbf{T}}}.$

*Subcase AIV* Taking $\varpi _{1}\ne 0$ and $\varpi _{2}\ne 0,$ employing the analogous technique as we did in **Subcasee AI**. Suppose that ${\textbf{U}}_{3}=({\mathcal {N}}{\textbf{Q}}_{2}^{3},{\mathcal {N}}{\textbf{Q}}_{2}^{2},{\mathcal {N}}{\textbf{Q}}_{2},{\mathcal {N}})^{{\textbf{T}}}$ so that ${\textbf{U}}_{3}{\textbf{Q}}_{2}{\textbf{U}}_{3}^{-1}={\textbf{Q}}_{1}.$ Hence, we have $({\textbf{U}}_{3}{\textbf{H}}_{2}{\textbf{H}}_{1})\Xi _{1}^{2}({\textbf{U}}_{3}{\textbf{H}}_{2}{\textbf{H}}_{1})^{{\textbf{T}}}+{\textbf{Q}}_{2}\big (({\textbf{U}}_{3}{\textbf{H}}_{2}{\textbf{H}}_{1})\Pi _{1}({\textbf{U}}_{3}{\textbf{H}}_{2}{\textbf{H}}_{1})^{{\textbf{T}}}\big )+\big (({\textbf{U}}_{3}{\textbf{H}}_{2}{\textbf{H}}_{1})\Pi _{1}({\textbf{U}}_{3}{\textbf{H}}_{2}{\textbf{H}}_{1})^{{\textbf{T}}}\big ){\textbf{Q}}_{3}^{{\textbf{T}}}=0,$ where $({\textbf{U}}_{3}{\textbf{H}}_{2}{\textbf{H}}_{1})\Pi _{1}({\textbf{U}}_{3}{\textbf{H}}_{2}{\textbf{H}}_{1})^{{\textbf{T}}}=({\chi _{22}}{\chi _{33}}\varpi _{2}\wp _{4\jmath -3})^{2}\Upsilon _{1}.$ Thus, we conclude that $\Pi _{1}=({\chi _{22}}{\chi _{33}}\varpi _{2}\wp _{4\jmath -3})^{2}({\textbf{U}}_{3}{\textbf{H}}_{2}{\textbf{H}}_{1})^{-1}\Upsilon _{1}\big (({\textbf{U}}_{3}{\textbf{H}}_{2}{\textbf{H}}_{1})\big )^{{\textbf{T}}}$ is a P-D matrix.

*Case B* Considering

$$\begin{aligned} \Xi _{2}^{2}+{\textbf{Q}}\Pi _{2}+\Pi _{2}{\textbf{Q}}^{{\textbf{T}}}=0. \end{aligned}$$

Assume that ${\textbf{H}}_{3}{\textbf{Q}}{\textbf{H}}_{3}={\textbf{Q}}_{3},$ where

$$\begin{aligned}{} & {} {\textbf{H}}_{3}=\begin{pmatrix} 0&{}1&{}0&{}0\\ 1&{}0&{}0&{}0\\ -{\chi _{42}}/{\chi _{12}}&{}0&{}0&{}1\\ -{\chi _{33}}/{\chi _{12}}&{}0&{}1&{}0 \end{pmatrix}, \nonumber \\ {}{} & {} {\textbf{Q}}_{3}=\begin{pmatrix} -{\chi _{22}}&{}{{\chi _{12}}{\chi _{22}}+{\chi _{22}}{\chi _{33}}}/{{\chi _{12}}}&{}0&{}{\chi _{22}}\\ {\chi _{12}}&{}-\big ({\chi _{11}}{\chi _{12}}+{\chi _{13}}{\chi _{33}}+{\chi _{14}}{\chi _{42}}/{\chi _{12}}\big )&{}{\chi _{14}}&{}-{\chi _{13}}\\ 0&{}\varpi _{3}&{}-\big ({\chi _{12}}{\chi _{41}}+{\chi _{12}}{\chi _{42}}+{\chi _{14}}{\chi _{42}}/{\chi _{12}}\big )&{}{\chi _{13}}{\chi _{42}}/{\chi _{12}}\\ 0&{}\varpi _{4}&{}-{\chi _{14}}{\chi _{23}}/{\chi _{12}}&{}{\chi _{13}}{\chi _{33}}-{\chi _{33}}{\chi _{12}}/{\chi _{12}}, \end{pmatrix}, \end{aligned}$$

where $$\varpi _{3}=\big ({\chi _{41}}{\chi _{12}}^{2}-{\chi _{14}}{\chi _{42}}^{2}+{\chi _{11}}{\chi _{12}}{\chi _{42}}+{\chi _{13}}{\chi _{33}}{\chi _{42}}-{\chi _{12}}{\chi _{42}}({\chi _{41}}+{\chi _{42}})/{\chi _{12}}^{2}\big )$$ and $$\varpi _{4}={\chi _{33}}\big ({\chi _{11}}{\chi _{12}}-{\chi _{12}}{\chi _{33}}+{\chi _{13}}{\chi _{33}}-{\chi _{14}}{\chi _{42}}/{\chi _{12}}^{2}\big ).$$

*Subcase BI* When $\varpi _{3}=0=\varpi _{4}$ and suppose that ${\textbf{Q}}_{3}={\textbf{U}}_{4}{\textbf{Q}}_{3}{\textbf{U}}_{4}^{-1},$ where

$$\begin{aligned}{} & {} {\textbf{U}}_{4}=\begin{pmatrix} {\chi _{12}}&{}-\big ({\chi _{11}}{\chi _{12}}+{\chi _{13}}{\chi _{33}}+{\chi _{14}}{\chi _{42}}/{\chi _{12}}\big )&{}{\chi _{14}}&{}-{\chi _{13}}\\ 0&{}1&{}0&{}0\\ 0&{}0&{}1&{}0\\ 0&{}0&{}0&{}1 \end{pmatrix},\nonumber \\ {}{} & {} {\textbf{Q}}_{3}=\begin{pmatrix} -\lambda _{4}&{}-\lambda _{5}&{}-\lambda _{6}&{}-\lambda _{7}\\ 1&{}0&{}0&{}0\\ 0&{}0&{}-\big ({\chi _{12}}({\chi _{41}}+{\chi _{42}})+{\chi _{14}}{\chi _{42}}/{\chi _{12}}\big )&{}{\chi _{13}}{\chi _{42}}/{\chi _{12}}\\ 0&{}0&{}-{\chi _{14}}{\chi _{33}}/{\chi _{12}}&{}{\chi _{13}}{\chi _{33}}-{\chi _{33}}{\chi _{12}}/{\chi _{12}} \end{pmatrix}, \end{aligned}$$

containing $$\lambda _{4}=\big ({\chi _{11}}{\chi _{12}}+{\chi _{12}}{\chi _{22}}+{\chi _{13}}{\chi _{33}}-{\chi _{14}}{\chi _{42}}/{\chi _{12}}\big )>0,$$ $$\lambda _{5}=\big (({\chi _{11}}-{\chi _{12}}){\chi _{12}}{\chi _{22}}+({\chi _{13}}-{\chi _{12}}){\chi _{22}}{\chi _{33}}-{\chi _{14}}{\chi _{42}}{\chi _{22}}/{\chi _{12}}\big )>0,$$ $$\lambda _{6}=\big (({\chi _{13}}-{\chi _{12}}){\chi _{12}}{\chi _{23}}+({\chi _{23}}-{\chi _{12}}){\chi _{22}}{\chi _{31}}-{\chi _{14}}{\chi _{42}}{\chi _{23}}/{\chi _{12}}\big )>0,$$ and $$\lambda _{7}=\big (({\chi _{33}}-{\chi _{12}}){\chi _{12}}{\chi _{13}}+({\chi _{42}}-{\chi _{12}}){\chi _{22}}{\chi _{13}}-{\chi _{14}}{\chi _{42}}{\chi _{14}}/{\chi _{12}}\big )>0.$$

In this way, we have

$$\begin{aligned} ({\textbf{U}}_{4}{\textbf{H}}_{3})\Xi _{2}^{2}({\textbf{U}}_{4}{\textbf{H}}_{3})^{{\textbf{T}}}+{\textbf{Q}}_{3}\big (({\textbf{U}}_{4}{\textbf{H}}_{3})\Pi _{2}({\textbf{U}}_{4}{\textbf{H}}_{3})^{{\textbf{T}}}\big )+\big (({\textbf{U}}_{4}{\textbf{H}}_{3})\Pi _{2}({\textbf{U}}_{4}{\textbf{H}}_{3})^{{\textbf{T}}}\big ){\textbf{Q}}_{3}^{{\textbf{T}}}. \end{aligned}$$

Taking into account Lemma 3, we have $({\textbf{U}}_{4}{\textbf{H}}_{3})\Pi _{2}({\textbf{U}}_{4}{\textbf{H}}_{3})^{{\textbf{T}}}$ is a semi P-D matrix and

$$\begin{aligned} ({\textbf{U}}_{4}{\textbf{H}}_{3})\Pi _{2}({\textbf{U}}_{4}{\textbf{H}}_{3})^{{\textbf{T}}}=({\chi _{12}}\wp _{4\jmath -2})^{2}\Upsilon _{3} \\ \Upsilon _{3}=\begin{pmatrix} (2\lambda _{4})^{-1}&{}0&{}0&{}0\\ 0&{}(2\lambda _{4}\lambda _{5})^{-1}&{}0&{}0\\ 0&{}0&{}0&{}0\\ 0&{}0&{}0&{}0 \end{pmatrix}. \end{aligned}$$

Finally, $$\Pi _{2}=({\chi _{12}}\wp _{4\jmath -2})^{2}({\textbf{U}}_{4}{\textbf{H}}_{3})^{-1}\Upsilon _{3}\big (({\textbf{U}}_{4}{\textbf{H}}_{3})^{-1}\big )^{{\textbf{T}}}.$$

*Subcase BII* When $\varpi _{3}\ne 0$ and $\varpi _{4}=0,$ applying the analogous approach as we did in the *Subcase AI*, then we attain ${\textbf{U}}_{5}=({\mathcal {N}}{\textbf{Q}}_{3}^{3},{\mathcal {N}}{\textbf{Q}}_{3}^{2},{\mathcal {N}}{\textbf{Q}}_{3},{\mathcal {N}})^{{\textbf{T}}}$ so that ${\textbf{U}}_{5}{\textbf{Q}}_{3}{\textbf{U}}_{5}^{-1}={\textbf{Q}}_{1}.$ hence, we get

$$\begin{aligned} ({\textbf{U}}_{5}{\textbf{H}}_{3})\Xi _{2}^{2}({\textbf{U}}_{5}{\textbf{H}}_{3})^{{\textbf{T}}}+{\textbf{Q}}_{1}\big (({\textbf{U}}_{5}{\textbf{H}}_{3})\Pi _{2}({\textbf{U}}_{5}{\textbf{H}}_{3})^{{\textbf{T}}}\big )+\big (({\textbf{U}}_{5}{\textbf{H}}_{3})\Pi _{2}({\textbf{U}}_{5}{\textbf{H}}_{3})^{{\textbf{T}}}\big ){\textbf{Q}}_{1}^{{\textbf{T}}}, \end{aligned}$$

where $$({\textbf{U}}_{5}{\textbf{H}}_{3})\Pi _{2}({\textbf{U}}_{5}{\textbf{H}}_{3})^{{\textbf{T}}}=({\chi _{14}}{\chi _{33}}\varpi _{3}\wp _{4\jmath -2})^{2}\Upsilon _{1}.$$ Thus,

92 $$\begin{aligned} \Pi _{2}=({\chi _{14}}{\chi _{33}}\varpi _{3}\wp _{4\jmath -2})^{2}({\textbf{U}}_{5}{\textbf{H}}_{3})^{-1}\Upsilon _{1}\big (({\textbf{U}}_{5}{\textbf{H}}_{3})^{-1}\big )^{{\textbf{T}}}, \end{aligned}$$

is a P-D matrix.

*Subcase BIII* When $\varpi _{3}=0$ and $\varpi _{4}\ne 0$ and suppose that ${\textbf{Q}}_{4}={\textbf{H}}_{4}{\textbf{Q}}_{3}{\textbf{H}}_{4}^{-1},$ where

$$\begin{aligned}{} & {} {\textbf{H}}_{4}=\begin{pmatrix} 1&{}0&{}0&{}0\\ 0&{}1&{}0&{}0\\ 0&{}0&{}0&{}1\\ 0&{}0&{}1&{}0 \end{pmatrix},\nonumber \\ {}{} & {} {\textbf{Q}}_{4}=\begin{pmatrix} -{\chi _{22}}&{}{\chi _{12}}{\chi _{22}}+{\chi _{22}}{\chi _{33}}/{\chi _{12}}&{}{\chi _{22}}&{}0\\ {\chi _{12}}&{}-\big ({\chi _{11}}{\chi _{12}}+{\chi _{13}}{\chi _{33}}+{\chi _{14}}{\chi _{42}}/{\chi _{12}}\big )&{}-{\chi _{13}}&{}{\chi _{14}}\\ 0&{}\varpi _{4}&{}{\chi _{13}}{\chi _{33}}-{\chi _{33}}{\chi _{12}}/{\chi _{12}}&{}-{\chi _{14}}{\chi _{33}}/{\chi _{12}}\\ 0&{}0&{}{\chi _{13}}{\chi _{42}}/{\chi _{12}}&{}-\big ({\chi _{12}}(\chi _{41}+{\chi _{42}})+{\chi _{14}}{\chi _{42}}/{\chi _{12}}\big ) \end{pmatrix}. \end{aligned}$$

Furthermore, we have ${\textbf{U}}_{6}=({\mathcal {N}}{\textbf{Q}}_{4}^{3},{\mathcal {N}}{\textbf{Q}}_{4}^{2},{\mathcal {N}}{\textbf{Q}}_{4},{\mathcal {N}})^{{\textbf{T}}}$ so that ${\textbf{U}}_{6}{\textbf{Q}}_{4}{\textbf{U}}_{6}^{-1}={\textbf{Q}}_{1}.$ Hence, we get

$$\begin{aligned} ({\textbf{U}}_{6}{\textbf{H}}_{4}{\textbf{H}}_{3})\Xi _{2}^{2}({\textbf{U}}_{6}{\textbf{H}}_{4}{\textbf{H}}_{3})^{{\textbf{T}}}+{\textbf{Q}}_{1}\big (({\textbf{U}}_{6}{\textbf{H}}_{4}{\textbf{H}}_{3})\Pi _{2}({\textbf{U}}_{6}{\textbf{H}}_{4}{\textbf{H}}_{3}\big )+\big (({\textbf{U}}_{6}{\textbf{H}}_{4}{\textbf{H}}_{3})\Pi _{2}({\textbf{U}}_{6}{\textbf{H}}_{4}{\textbf{H}}_{3})^{{\textbf{T}}}\big ){\textbf{Q}}_{1}^{{\textbf{T}}}, \end{aligned}$$

where $$({\textbf{U}}_{6}{\textbf{H}}_{4}{\textbf{H}}_{3})\Pi _{2}({\textbf{U}}_{6}{\textbf{H}}_{4}{\textbf{H}}_{3})^{{\textbf{T}}}=({\chi _{13}}{\chi _{42}}\varpi _{4}\wp _{4\jmath -2})^{2}\Upsilon _{1}.$$ Thus,

$$\begin{aligned} \Pi _{2}=({\chi _{13}}{\chi _{42}}\varpi _{4}\wp _{4\jmath -2})^{2}({\textbf{U}}_{6}{\textbf{H}}_{4}{\textbf{H}}_{3})^{-1}\Upsilon _{1}\big (({\textbf{U}}_{6}{\textbf{H}}_{4}{\textbf{H}}_{3})^{-1}\big )^{{\textbf{T}}}, \end{aligned}$$

is a P-D matrix.

*Subcase BIV* When $\varpi _{3}=\varpi _{4}\ne 0$ and suppose that ${\textbf{Q}}_{5}={\textbf{H}}_{5}{\textbf{Q}}_{3}{\textbf{H}}_{5}^{-1},$ where

$$\begin{aligned}{} & {} {\textbf{H}}_{5}=\begin{pmatrix} 1&{}0&{}0&{}0\\ 0&{}1&{}0&{}0\\ 0&{}0&{}1&{}0\\ 0&{}0&{}-{\varpi _{4}}/{\varpi _{3}}&{}1 \end{pmatrix},\nonumber \\{} & {} {\textbf{Q}}_{5}=\begin{pmatrix} -{\chi _{22}}&{}{\chi _{12}}{\chi _{22}}+{\chi _{22}}{\chi _{33}}/{\chi _{12}}&{}{\chi _{22}}{\varpi _{4}}/{\varpi _{3}}&{}{\chi _{22}}\\ {\chi _{12}}&{}-\big ({\chi _{11}}{\chi _{12}}+{\chi _{13}}{\chi _{33}}+{\chi _{14}}{\chi _{42}}/{\chi _{12}}\big )&{}{\chi _{14}}-{\chi _{13}}{\varpi _{4}}/{\varpi _{3}}&{}-{\chi _{13}}\\ 0&{}\varpi _{4}&{}-\lambda _{8}&{}{\chi _{13}}{\chi _{42}}/{\chi _{12}}\\ 0&{}0&{}\varpi _{5}&{}\frac{\varpi _{3}(\chi _{13}\chi _{33}-\chi _{33}\chi _{12})-\chi _{13}\chi _{32}\varpi _{4}}{\varpi _{3}\chi _{12}} \end{pmatrix}. \end{aligned}$$

Furthermore, we have ${\textbf{U}}_{6}=({\mathcal {N}}{\textbf{Q}}_{4}^{3},{\mathcal {N}}{\textbf{Q}}_{4}^{2},{\mathcal {N}}{\textbf{Q}}_{4},{\mathcal {N}})^{{\textbf{T}}}$ so that ${\textbf{U}}_{6}{\textbf{Q}}_{4}{\textbf{U}}_{6}^{-1}={\textbf{Q}}_{1}.$ Hence, we get

$$\begin{aligned} ({\textbf{U}}_{6}{\textbf{H}}_{4}{\textbf{H}}_{3})\Xi _{2}^{2}({\textbf{U}}_{6}{\textbf{H}}_{4}{\textbf{H}}_{3})^{{\textbf{T}}}+{\textbf{Q}}_{1}\big (({\textbf{U}}_{6}{\textbf{H}}_{4}{\textbf{H}}_{3})\Pi _{2}({\textbf{U}}_{6}{\textbf{H}}_{4}{\textbf{H}}_{3}\big )+\big (({\textbf{U}}_{6}{\textbf{H}}_{4}{\textbf{H}}_{3})\Pi _{2}({\textbf{U}}_{6}{\textbf{H}}_{4}{\textbf{H}}_{3})^{{\textbf{T}}}\big ){\textbf{Q}}_{1}^{{\textbf{T}}}, \end{aligned}$$

where $$({\textbf{U}}_{6}{\textbf{H}}_{4}{\textbf{H}}_{3})\Pi _{2}({\textbf{U}}_{6}{\textbf{H}}_{4}{\textbf{H}}_{3})^{{\textbf{T}}}=({\chi _{13}}{\chi _{42}}\varpi _{4}\wp _{4\jmath -2})^{2}\Upsilon _{1}.$$ Thus,

$$\begin{aligned} \Pi _{2}=({\chi _{13}}{\chi _{42}}\varpi _{4}\wp _{4\jmath -2})^{2}({\textbf{U}}_{6}{\textbf{H}}_{4}{\textbf{H}}_{3})^{-1}\Upsilon _{1}\big (({\textbf{U}}_{6}{\textbf{H}}_{4}{\textbf{H}}_{3})^{-1}\big )^{{\textbf{T}}}, \end{aligned}$$

is a P-D matrix.

*Subcase BV* When $\varpi _{3}=\varpi _{4}\ne 0$ and $\varpi _{5}=0,$ applying the identical technique from *Subcase AIII*), we obtain ${\textbf{U}}_{7}{\textbf{Q}}_{5}{\textbf{U}}_{7}^{-1}={\textbf{Q}}_{4},$ where

$$\begin{aligned} {\textbf{U}}_{7}=\begin{pmatrix} {\chi _{12}}\varpi _{4}&{}-\lambda _{9}&{}\lambda _{10}&{}0\\ 0&{}\varpi _{4}&{}-\lambda _{8}&{}0\\ 0&{}0&{}1&{}0\\ 0&{}0&{}0&{}1 \end{pmatrix},~~ , ~~{\textbf{Q}}_{4}=\begin{pmatrix} -\lambda _{11}&{}-\lambda _{12}&{}-\lambda _{13}&{}\lambda _{14}\\ 1&{}0&{}0&{}\lambda _{15}\\ 01&{}1&{}0&{}\lambda _{16}\\ 0&{}0&{}0&{}\lambda _{17} \end{pmatrix}, \end{aligned}$$

having

$$\begin{aligned} \lambda _{9}= & {} \Big (\frac{{\chi _{12}}{\chi _{11}}+{\chi _{13}}{\chi _{33}}-{\chi _{14}}{\chi _{42}}}{{\chi _{12}}}+\lambda _{8}\Big ),~\lambda _{10}=\lambda _{8}^{2}+\Big (\frac{\varpi _{3}{\chi _{14}}+{\chi _{13}}\varpi _{4}}{\varpi _{3}}\Big )\varpi _{4},~~\\ \lambda _{11}= & {} {\left\{ \begin{array}{ll} \Bigg \{\big ({\chi _{11}}{\chi _{41}}{\chi _{12}}^{2}+{\chi _{12}}{\chi _{42}}{\chi _{11}}^{2}-{\chi _{11}}{\chi _{14}}{\chi _{42}}^{2}+{\chi _{22}}{\chi _{41}}{\chi _{12}}^{2}-{\chi _{14}}{\chi _{22}}{\chi _{42}}^{2}\\ \quad +({\chi _{41}}{\chi _{12}}^{2}-{\chi _{14}}{\chi _{42}}^{2}-{\chi _{12}}{\chi _{22}}{\chi _{42}})({\chi _{41}}+{\chi _{42}})-{\chi _{12}}{\chi _{42}}({\chi _{41}}+{\chi _{42}})^{2}\\ \quad +{\chi _{11}}{\chi _{12}}{\chi _{22}}{\chi _{42}}+{\chi _{11}}{\chi _{13}}{\chi _{33}}{\chi _{41}}+{\chi _{13}}{\chi _{22}}{\chi _{33}}{\chi _{42}}+{\chi _{13}}{\chi _{33}}^{2}{\chi _{42}}\big ){\chi _{12}}^{2}/\varpi _{3}\Bigg \}>0,\end{array}\right. } \\ \lambda _{12}= & {} {\left\{ \begin{array}{ll} \Bigg \{{\chi _{13}}^{2}{\chi _{33}}^{2}{\chi _{41}}-{\chi _{12}}^{2}{\chi _{41}}^{2}{\chi _{14}}+{\chi _{14}}^{2}{\chi _{42}}^{2}{\chi _{41}}-{\chi _{12}}^{3}{\chi _{22}}{\chi _{41}}-{\chi _{11}}{\chi _{12}}^{2}{\chi _{22}}{\chi _{42}}\\ \quad +{\chi _{11}}{\chi _{12}}^{2}{\chi _{22}}{\chi _{41}}+{\chi _{11}}^{2}{\chi _{12}}{\chi _{22}}{\chi _{42}}-{\chi _{11}}{\chi _{14}}{\chi _{22}}{\chi _{42}}^{2}+{\chi _{12}}{\chi _{14}}{\chi _{22}}{\chi _{42}}^{2}\\ \quad +{\chi _{11}}{\chi _{12}}^{2}{\chi _{41}}({\chi _{41}}+{\chi _{42}})-{\chi _{12}}^{2}{\chi _{22}}{\chi _{33}}{\chi _{41}}-{\chi _{11}}{\chi _{12}}{\chi _{42}}({\chi _{41}}+{\chi _{42}})^{2}\\ \quad +{\chi _{11}}^{2}{\chi _{12}}{\chi _{42}}({\chi _{41}}+{\chi _{42}})-{\chi _{13}}{\chi _{22}}{\chi _{42}}{\chi _{33}}^{2}-{\chi _{11}}{\chi _{14}}{\chi _{42}}^{2}(\chi _{41}+{\chi _{42}})\\ \quad +{\chi _{14}}{\chi _{22}}{\chi _{33}}{\chi _{42}}^{2}+{\chi _{12}}^{2}{\chi _{22}}{\chi _{42}}({\chi _{41}}+{\chi _{42}})+{\chi _{12}}^{2}{\chi _{22}}{\chi _{41}}(\chi _{41}+{\chi _{42}})\\ \quad -{\chi _{12}}{\chi _{22}}{\chi _{42}}(\chi _{41}+{\chi _{42}})^{2}-{\chi _{14}}{\chi _{22}}{\chi _{42}}^{2}(\chi _{41}+{\chi _{42}})-{\chi _{13}}{\chi _{33}}{\chi _{42}}(\chi _{41}+{\chi _{42}})^{2}\\ \quad +{\chi _{11}}{\chi _{12}}{\chi _{13}}{\chi _{33}}{\chi _{41}}-{\chi _{11}}{\chi _{12}}{\chi _{14}}{\chi _{42}}{\chi _{41}}-{\chi _{11}}{\chi _{12}}{\chi _{22}}{\chi _{33}}{\chi _{42}}+{\chi _{11}}{\chi _{13}}{\chi _{22}}{\chi _{33}}{\chi _{42}}\\ \quad -{\chi _{12}}{\chi _{13}}{\chi _{22}}{\chi _{33}}{\chi _{42}}+{\chi _{12}}{\chi _{13}}{\chi _{22}}{\chi _{33}}{\chi _{41}}-{\chi _{12}}{\chi _{13}}{\chi _{33}}{\chi _{33}}{\chi _{41}}+{\chi _{11}}{\chi _{13}}{\chi _{33}}{\chi _{42}}({\chi _{41}}+{\chi _{42}})\\ \quad +{\chi _{12}}{\chi _{13}}{\chi _{33}}{\chi _{41}}({\chi _{41}}+{\chi _{42}})-2{\chi _{13}}{\chi _{14}}{\chi _{33}}{\chi _{41}}{\chi _{42}}+{\chi _{13}}{\chi _{22}}{\chi _{33}}{\chi _{33}}{\chi _{42}}+{\chi _{12}}{\chi _{14}}{\chi _{41}}{\chi _{42}}({\chi _{41}}+{\chi _{42}})\\ \quad +{\chi _{12}}{\chi _{22}}{\chi _{33}}{\chi _{42}}({\chi _{41}}+{\chi _{42}})+{\chi _{13}}{\chi _{33}}{\chi _{33}}{\chi _{42}}({\chi _{41}}+{\chi _{42}}){\chi _{12}}^{2}/\varpi _{3}\Bigg \}>0, \end{array}\right. }\\ \lambda _{13}= & {} {\left\{ \begin{array}{ll}-\Bigg \{\big ({\chi _{11}}^{2}{\chi _{22}}{\chi _{12}}{\chi _{33}}{\chi _{42}}-{\chi _{11}}^{2}{\chi _{22}}{\chi _{12}}{\chi _{42}}({\chi _{41}}+{\chi _{42}})+{\chi _{12}}^{2}{\chi _{11}}{\chi _{22}}{\chi _{33}}{\chi _{41}}-{\chi _{12}}^{2}{\chi _{11}}{\chi _{22}}{\chi _{41}}({\chi _{41}}+{\chi _{42}})\\ \quad +{\chi _{12}}^{2}{\chi _{11}}{\chi _{22}}{\chi _{42}}({\chi _{41}}+{\chi _{42}})-{\chi _{22}}{\chi _{11}}{\chi _{12}}{\chi _{13}}{\chi _{33}}{\chi _{41}}-{\chi _{22}}{\chi _{11}}{\chi _{12}}{\chi _{13}}{\chi _{33}}{\chi _{42}}+{\chi _{22}}{\chi _{11}}{\chi _{12}}{\chi _{14}}{\chi _{41}}{\chi _{42}}\\ \quad +{\chi _{22}}{\chi _{11}}{\chi _{12}}{\chi _{14}}{\chi _{42}}^{2}-{\chi _{22}}{\chi _{33}}{\chi _{11}}{\chi _{12}}{\chi _{33}}{\chi _{42}}+{\chi _{22}}{\chi _{11}}{\chi _{12}}{\chi _{13}}{\chi _{42}}({\chi _{41}}+{\chi _{42}})^{2}+{\chi _{22}}{\chi _{11}}{\chi _{13}}{\chi _{33}}^{2}{\chi _{42}}\\ \quad -{\chi _{22}}{\chi _{11}}{\chi _{13}}{\chi _{33}}{\chi _{42}}({\chi _{41}}+{\chi _{42}})-{\chi _{22}}{\chi _{11}}{\chi _{14}}{\chi _{33}}{\chi _{42}}^{2}+{\chi _{22}}{\chi _{11}}{\chi _{14}}{\chi _{42}}^{2}({\chi _{41}}+{\chi _{42}})+{\chi _{22}}{\chi _{12}}^{3}{\chi _{41}}({\chi _{41}}\\ \quad +{\chi _{42}})+{\chi _{22}}{\chi _{12}}^{2}{\chi _{14}}{\chi _{41}}^{2}+{\chi _{22}}{\chi _{14}}{\chi _{41}}{\chi _{42}}{\chi _{12}}^{2}+{\chi _{22}}{\chi _{12}}^{2}{\chi _{33}}{\chi _{41}}({\chi _{41}}+{\chi _{42}})-{\chi _{22}}{\chi _{33}}{\chi _{12}}^{2}{\chi _{33}}{\chi _{41}}\\ \quad -{\chi _{22}}{\chi _{42}}{\chi _{12}}^{2}({\chi _{41}}+{\chi _{42}})^{2}+{\chi _{22}}{\chi _{12}}{\chi _{13}}{\chi _{41}}{\chi _{33}}^{2}-{\chi _{22}}{\chi _{12}}{\chi _{13}}{\chi _{33}}{\chi _{41}}({\chi _{41}}+{\chi _{42}})+{\chi _{22}}{\chi _{33}}{\chi _{12}}{\chi _{13}}{\chi _{33}}{\chi _{41}}\\ \quad +{\chi _{22}}{\chi _{12}}{\chi _{13}}{\chi _{33}}{\chi _{42}}({\chi _{41}}+{\chi _{42}})+{\chi _{22}}{\chi _{33}}{\chi _{12}}{\chi _{13}}{\chi _{33}}{\chi _{42}}-{\chi _{22}}{\chi _{12}}{\chi _{14}}{\chi _{41}}{\chi _{42}}({\chi _{41}}+{\chi _{42}})\\ \quad -2{\chi _{22}}{\chi _{12}}{\chi _{14}}{\chi _{42}}^{2}({\chi _{41}}+{\chi _{42}})-{\chi _{22}}{\chi _{12}}{\chi _{33}}{\chi _{42}}({\chi _{41}}+{\chi _{42}})^{2}+{\chi _{22}}{\chi _{13}}{\chi _{33}}{\chi _{42}}({\chi _{41}}+{\chi _{42}})^{2}\\ \quad -{\chi _{22}}{\chi _{33}}{\chi _{13}}{\chi _{33}}{\chi _{42}}({\chi _{41}}+{\chi _{42}})-{\chi _{22}}{\chi _{41}}{\chi _{22}}{\chi _{14}}^{4}{\chi _{42}}^{2}{\chi _{42}}^{3}-{\chi _{22}}{\chi _{14}}{\chi _{33}}{\chi _{42}}^{2}({\chi _{41}}\\ \quad +{\chi _{42}})+{\chi _{22}}{\chi _{33}}{\chi _{14}}{\chi _{33}}{\chi _{42}}^{2}\big ){\chi _{12}}^{2}/\varpi _{3} \Bigg \}>0. \end{array}\right. } \end{aligned}$$

Moreover, $\lambda 13-\lambda _{11}\lambda _{12}<0,~\lambda _{14},~\lambda _{15},\lambda _{16}$ and $\lambda _{17}$ will be determined later.

Then, we obtain

$$\begin{aligned} ({\textbf{U}}_{7}{\textbf{H}}_{5}{\textbf{H}}_{3})\Xi _{2}^{2}({\textbf{U}}_{7}{\textbf{H}}_{5}{\textbf{H}}_{3})^{{\textbf{T}}}+{\textbf{Q}}_{4}\big (({\textbf{U}}_{7}{\textbf{H}}_{5}{\textbf{H}}_{3})\Pi _{2}({\textbf{U}}_{7}{\textbf{H}}_{5}{\textbf{H}}_{3}\big )+\big (({\textbf{U}}_{7}{\textbf{H}}_{5}{\textbf{H}}_{3})\Pi _{2}({\textbf{U}}_{7}{\textbf{H}}_{5}{\textbf{H}}_{3})^{{\textbf{T}}}\big ){\textbf{Q}}_{4}^{{\textbf{T}}}=0, \end{aligned}$$

where $$ ({\textbf{U}}_{7}{\textbf{H}}_{5}{\textbf{H}}_{3})\Pi _{2}({\textbf{U}}_{7}{\textbf{H}}_{5}{\textbf{H}}_{3})^{{\textbf{T}}}=({\chi _{12}}\varpi _{4}\wp _{4\jmath -2})^{2}\Upsilon _{4}$$ and

$$\begin{aligned} \Upsilon _{4}=\begin{pmatrix} \big (\lambda _{12}/2(\lambda _{11}\lambda _{12}-\lambda _{13})\big )&{}0&{}-\big (1/2(\lambda _{11}\lambda _{12}-\lambda _{13})\big )&{}0\\ 0&{}\big (1/2(\lambda _{11}\lambda _{12}-\lambda _{13})\big )&{}0&{}0\\ -\big (1/2(\lambda _{11}\lambda _{12}-\lambda _{13})\big )&{}0&{}\big (\lambda _{11}/2(\lambda _{11}\lambda _{12}-\lambda _{13})\big )&{}0\\ 0&{}0&{}0&{}0 \end{pmatrix}. \end{aligned}$$

Therefore, $$\Pi _{2}=({\chi _{12}}\varpi _{4}\wp _{4\jmath -2})^{2}({\textbf{U}}_{7}{\textbf{H}}_{5}{\textbf{H}}_{3})^{-1}\Upsilon _{4}\big (({\textbf{U}}_{7}{\textbf{H}}_{5}{\textbf{H}}_{3})^{-1}\big )^{{\textbf{T}}}.$$

*Subcase BVI* If $\varpi _{3}=\varpi _{4}=\varpi _{5}\ne 0$ and applying the analogous approach as we did in *Subcase AIV*. Assume that ${\textbf{U}}_{8}=\big ({\mathcal {N}}{\textbf{Q}}_{5}^{3},{\mathcal {N}}{\textbf{Q}}_{5}^{2},{\mathcal {N}}{\textbf{Q}}_{5},{\mathcal {N}}\big )^{{\textbf{T}}}$ so that ${\textbf{U}}_{8}{\textbf{Q}}_{5}{\textbf{Q}}_{8}^{-1}={\textbf{Q}}_{1}.$ Hence, we have

$$\begin{aligned} ({\textbf{U}}_{8}{\textbf{H}}_{5}{\textbf{H}}_{3})\Xi _{2}^{2}({\textbf{U}}_{8}{\textbf{H}}_{5}{\textbf{H}}_{3})^{{\textbf{T}}}+{\textbf{Q}}_{1}\big (({\textbf{U}}_{8}{\textbf{H}}_{5}{\textbf{H}}_{3})\Pi _{2}({\textbf{U}}_{8}{\textbf{H}}_{5}{\textbf{H}}_{3}\big )+\big (({\textbf{U}}_{8}{\textbf{H}}_{5}{\textbf{H}}_{3})\Pi _{2}({\textbf{U}}_{8}{\textbf{H}}_{5}{\textbf{H}}_{3})^{{\textbf{T}}}\big ){\textbf{Q}}_{1}^{{\textbf{T}}}=0, \end{aligned}$$

where $({\textbf{U}}_{8}{\textbf{H}}_{5}{\textbf{H}}_{3})\Pi _{2}({\textbf{U}}_{8}{\textbf{H}}_{5}{\textbf{H}}_{3})^{{\textbf{T}}}=({\chi _{12}}\varpi _{4}\varpi _{5}\wp _{4\jmath -2})^{2}\Upsilon _{1}=0.$Therefore, $\Pi _{2}=({\chi _{12}}\varpi _{4}\varpi _{5}\wp _{4\jmath -2})^{2}({\textbf{U}}_{8}{\textbf{H}}_{5}{\textbf{H}}_{3})^{-1}\Upsilon _{1}\big (({\textbf{U}}_{8}{\textbf{H}}_{5}{\textbf{H}}_{3})^{-1}\big )^{{\textbf{T}}}$ is a P-D matrix.

*Case C*  Surmise that $\Xi _{3}^{2}+{\textbf{Q}}\Pi _{3}+\Pi _{3}{\textbf{Q}}^{{\textbf{T}}}=0.$

Assume that ${\textbf{Q}}_{6}={\textbf{H}}_{6}{\textbf{Q}}{\textbf{H}}_{6}^{-1},$ where

$$\begin{aligned} {\textbf{H}}_{6}=\begin{pmatrix} 0&{}0&{}1&{}0\\ 1&{}0&{}0&{}0\\ {{\chi _{22}}}/{\chi _{13}}&{}1&{}0&{}0\\ 0&{}0&{}0&{}1 \end{pmatrix},~~and~~{\textbf{Q}}_{6}=\begin{pmatrix} -{\chi _{33}}&{}-{\chi _{22}}{\chi _{33}}/{{\chi _{13}}}&{}{\chi _{33}}&{}0\\ -{\chi _{13}}&{}-\big ({\chi _{11}}{\chi _{13}}+{\chi _{12}}{\chi _{22}}/{\chi _{13}}\big )&{}{\chi _{12}}&{}{\chi _{14}}\\ 0&{}\varpi _{6}&{}\big ({\chi _{12}}{\chi _{22}}-{\chi _{22}}{\chi _{12}}/{\chi _{13}}\big )&{}{\chi _{14}}{\chi _{22}}/{\chi _{13}}\\ 0&{}0&{}{\chi _{42}}&{}-({\chi _{41}}+{\chi _{42}}) \end{pmatrix}, \end{aligned}$$

having $$\varpi _{6}=\big ({\chi _{13}}^{2}-{\chi _{12}}{\chi _{22}}-{\chi _{11}}{\chi _{13}}+{\chi _{13}}{\chi _{22}}\big ){\chi _{22}}/{\chi _{13}}^ {2}.$$

*Subcase CI* When $\varpi _{6}=0,$ employing the identical approach as we applied in *Subcase AIII* and consider that ${\textbf{Q}}_{5}={\textbf{U}}_{9}{\textbf{Q}}_{6}{\textbf{U}}_{9}^{-1},$ where

93 $$\begin{aligned} {\textbf{U}}_{9}=\begin{pmatrix} -{\chi _{13}}&{}-\big ({\chi _{11}}{\chi _{13}}+{\chi _{12}}{\chi _{22}}/{\chi _{13}}\big )&{}{\chi _{12}}&{}{\chi _{14}}\\ 0&{}1&{}0&{}0\\ 0&{}0&{}1&{}0\\ 0&{}0&{}0&{}1 \end{pmatrix},~~and~~{\textbf{Q}}_{5}=\begin{pmatrix} -\lambda _{18}&{}-\lambda _{19}&{}-\lambda _{20}&{}-\lambda _{22}\\ 1&{}0&{}0&{}0\\ 0&{}0&{}\big ({\chi _{12}}{\chi _{22}}-{\chi _{22}}{\chi _{13}}/{\chi _{13}}\big )&{}{\chi _{14}}{\chi _{22}}/{\chi _{13}}\\ 0&{}0&{}{\chi _{42}}&{}-({\chi _{41}}+{\chi _{42}}) \end{pmatrix}, \end{aligned}$$

containing $\lambda _{18}=({\chi _{11}}{\chi _{13}}+{\chi _{12}}{\chi _{22}}+{\chi _{13}}{\chi _{33}})/{\chi _{13}},~~\lambda _{19}=({\chi _{11}}{\chi _{13}}{\chi _{33}}+{\chi _{12}}{\chi _{22}}{\chi _{33}}-{\chi _{13}}{\chi _{33}}{\chi _{22}})/{\chi _{13}},~~\lambda _{20}$ and $\lambda _{22}$ will be computed later.

Thus, we find

$$\begin{aligned} ({\textbf{U}}_{9}{\textbf{H}}_{6})\Xi _{3}^{2}({\textbf{U}}_{9}{\textbf{H}}_{6})^{{\textbf{T}}}+{\textbf{Q}}_{5}\big (({\textbf{U}}_{9}{\textbf{H}}_{6})\Pi _{3}({\textbf{U}}_{9}{\textbf{H}}_{6})^{{\textbf{T}}}\big )+\big (({\textbf{U}}_{9}{\textbf{H}}_{6})\Pi _{3}({\textbf{U}}_{9}{\textbf{H}}_{6})^{{\textbf{T}}}\big ){\textbf{Q}}_{5}^{{\textbf{T}}}=0. \end{aligned}$$

Using the fact of Lemma 3, we have $({\textbf{U}}_{9}{\textbf{H}}_{6})\Pi _{3}({\textbf{U}}_{9}{\textbf{H}}_{6})^{{\textbf{T}}}=({\chi _{13}}\wp _{4\jmath -1})^{2}\Upsilon _{5},$ where

$$\begin{aligned} \Upsilon _{5}=\begin{pmatrix}(2\lambda _{18})^{-1}&{}0&{}0&{}0\\ 0&{}(2\lambda _{18}\lambda _{19})^{-1}&{}0&{}0\\ 0&{}0&{}0&{}0\\ 0&{}0&{}0&{}0 \end{pmatrix}. \end{aligned}$$

Consequently, $$\Pi _{3}=({\chi _{13}}\wp _{4\jmath -1})^{2}({\textbf{U}}_{9}{\textbf{H}}_{6})^{-1}\Upsilon _{5}\big (({\textbf{U}}_{9}{\textbf{H}}_{6})^{-1}\big )^{{\textbf{T}}}.$$

*Subcase CII* When $\varpi _{6}\ne 0,$ then applying *Subcase AI* with a similar technique, resulting in ${\textbf{U}}_{10}=\big ({\mathcal {N}}{\textbf{Q}}_{6}^{3},{\mathcal {N}}{\textbf{Q}}_{6}^{2},{\mathcal {N}}{\textbf{Q}}_{6},{\mathcal {N}}\big )^{{\textbf{T}}}$ so that ${\textbf{U}}_{10}{\textbf{Q}}_{6}{\textbf{U}}_{10}^{-1}={\textbf{Q}}_{1},$ which leads to

$$\begin{aligned} ({\textbf{U}}_{10}{\textbf{H}}_{6})\Xi _{3}^{2}({\textbf{U}}_{10}{\textbf{H}}_{6})^{{\textbf{T}}}+{\textbf{Q}}_{1}\big (({\textbf{U}}_{10}{\textbf{H}}_{6})\Pi _{3}({\textbf{U}}_{10}{\textbf{H}}_{6})^{{\textbf{T}}}\big )+\big (({\textbf{U}}_{10}{\textbf{H}}_{6})\Pi _{3}({\textbf{U}}_{10}{\textbf{H}}_{6})^{{\textbf{T}}}\big ){\textbf{Q}}_{1}^{{\textbf{T}}}=0, \end{aligned}$$

where

$$\begin{aligned} \big (({\textbf{U}}_{10}{\textbf{H}}_{6})\Pi _{3}({\textbf{U}}_{10}{\textbf{H}}_{6})^{{\textbf{T}}}\big )\Pi _{3}\big (({\textbf{U}}_{10}{\textbf{H}}_{6})\Pi _{3}({\textbf{U}}_{10}{\textbf{H}}_{6})^{{\textbf{T}}}\big )^{{\textbf{T}}}=({\chi _{13}}{\chi _{42}}\varpi _{6}\wp _{4\jmath -1})^{2}\Upsilon _{1}. \end{aligned}$$

Hence, we conclude that $\Pi _{3}=({\chi _{13}}{\chi _{42}}\varpi _{6}\wp _{4\jmath -1})^{2}({\textbf{U}}_{10}{\textbf{H}}_{6})^{-1}\Upsilon _{1}\big (({\textbf{U}}_{10}{\textbf{H}}_{6})^{-1}\big )^{{\textbf{T}}}$ is a P-D matrix.

*Case D* Considering $\Xi _{4}^{2}+{\textbf{Q}}\Pi _{4}+\Pi _{4}{\textbf{Q}}^{{\textbf{T}}}=0,$ and also, we have ${\textbf{Q}}_{7}={\textbf{H}}_{7}{\textbf{Q}}{\textbf{H}}_{7}^{-1},$ where

$$\begin{aligned} {\textbf{H}}_{7}=\begin{pmatrix} 0&{}0&{}0&{}1\\ 1&{}0&{}0&{}0\\ 0&{}1&{}0&{}0\\ 0&{}0&{}1&{}0 \end{pmatrix},~~{\textbf{Q}}_{7}=\begin{pmatrix} -(\chi _{41}+{\chi _{42}})&{}{\chi _{41}}&{}{\chi _{42}}&{}0\\ {\chi _{41}}&{}-{\chi _{11}}&{}{\chi _{12}}&{}-{\chi _{13}}\\ 0&{}{\chi _{22}}&{}-{\chi _{22}}&{}{\chi _{22}}\\ 0&{}0&{}{\chi _{33}}&{}-{\chi _{33}} \end{pmatrix}. \end{aligned}$$

Indicate ${\textbf{U}}_{11}=\big ({\mathcal {N}}{\textbf{Q}}_{7}^{3},{\mathcal {N}}{\textbf{Q}}_{7}^{2},{\mathcal {N}}{\textbf{Q}}_{7},{\mathcal {N}}\big )^{{\textbf{T}}}$ so that ${\textbf{U}}_{11}{\textbf{Q}}_{7}{\textbf{U}}_{11}^{-1}={\textbf{Q}}_{1}.$ Thus, we get

$$\begin{aligned} ({\textbf{U}}_{11}{\textbf{H}}_{7})\Xi _{4}^{2}({\textbf{U}}_{11}{\textbf{H}}_{7})^{{\textbf{T}}}+\mathcal {{\mathbb {B}}_{1}}\big (({\textbf{U}}_{11}{\textbf{H}}_{7})\Pi _{4}({\textbf{U}}_{11}{\textbf{H}}_{7})^{{\textbf{T}}}\big )+\big (({\textbf{U}}_{11}{\textbf{H}}_{7})\Pi _{4}({\textbf{U}}_{11}{\textbf{H}}_{7})^{{\textbf{T}}}\big ){\textbf{Q}}_{1}^{{\textbf{T}}}=0, \end{aligned}$$

where

$$\begin{aligned} ({\textbf{U}}_{11}{\textbf{H}}_{7})\Pi _{4}\big (({\textbf{U}}_{11}{\textbf{H}}_{7})\big )^{{\textbf{T}}}=({\chi _{14}}{\chi _{22}}{\chi _{33}}\wp _{4\jmath })^{2}\Upsilon _{1}. \end{aligned}$$

This concludes that $\Pi _{4}=({\chi _{14}}{\chi _{22}}{\chi _{33}}\wp _{4\jmath })^{2}({\textbf{U}}_{11}{\textbf{H}}_{7})^{-1}\Upsilon _{1}\big (({\textbf{U}}_{11}{\textbf{H}}_{7})^{-1}\big )^{{\textbf{T}}}$ is a P-D matrix. Finally, the expression $\Pi =\Pi _{\ell },~(\ell =1,...,4)$ is a P-D matrix. So, the solution $\big ({{\textbf{S}}}(\tau ),{{\textbf{E}}}_{{\textbf{C}}}(\tau ),{{\textbf{I}}}_{{\textbf{C}}}(\tau ),{{\textbf{R}}}(\tau )\big )$ of model (75) possess a log-normal P.D.F ${\mathcal {U}}({\tilde{\Psi }})$ about ${\textbf{U}}_{\jmath }^{*}$ as

$$\begin{aligned} {\mathcal {U}}({\tilde{\Psi }})=\frac{1}{4\varphi _{2}^{2}}\vert \Pi \vert ^{-1/2}\exp \Big (\frac{-1}{2}{\tilde{\Psi }}\Pi ^{-1}{\tilde{\Psi }}^{{\textbf{T}}}\Big ). \end{aligned}$$

This yields the intended result. $\square$

## Numerical solutions of co-dynamics model using random perturbations

The computation methods of stochastic perturbations influence whenever differentiating expressions involve fractional differential compositions involving singular or nonsingular kernels, and classical prescriptions include this component. The fractional notions have an order corresponding to 0 and 1.

### Caputo fractional derivative operator

The main objective of this study is to investigate the co-infection of the TB and COVID-19 models involving integer-order (2), power-law (3) and stochastic strategy for (28). This scheme incorporates substantial pulmonary inflammation, which makes the circulatory mechanism a key battleground for numerous ailments. In the situation where $\intercal$ is chosen as the final propagation period, the mathematical framework will be built using the classical-order expression in the beginning, the power-law memory considered in the next step, and the stochastic configuration in the stages that follow. After the fact that the subsequent number pattern is provided to explain the incidence.

Specifically, we analyze the sectionally divided frameworks (2), (3) and (28) quantitatively by using the procedure given in^29^ in the context of CFD. In order to outline the procedure, we conducted what follows:

$$\begin{aligned} {\left\{ \begin{array}{ll} \frac{d\mho _{\iota }(\tau )}{d\tau }=\digamma (\tau ,\mho _{\iota }).~\mho _{\iota }(0)=\mho _{\iota ,0},~\iota =1,2,...,{\mathfrak {n}}~if~\tau \in [0,\intercal _{1}],\\ \,_{\intercal _{1}}^{c}{\textbf{D}}_{\tau }^{\omega }\mho _{\iota }(\tau )=\digamma (\tau ,\mho _{\iota }),~\mho _{\iota }(\intercal _{1})=\mho _{\iota ,1},~if~\tau \in [\intercal _{1},\intercal _{2}],\\ d\mho _{\iota }(\tau )=\digamma (\tau ,\mho _{\iota })d\tau +\wp _{\iota }\mho _{\iota }d{\mathbb {B}}_{\iota }(\tau ),~\mho _{\iota }(\intercal _{2})=\mho _{\iota ,2},~if~\tau \in [\intercal _{2},\intercal ]. \end{array}\right. } \end{aligned}$$

Thus, it implies that

$$\begin{aligned} \mho _{\iota }^{{\textbf{v}}}={\left\{ \begin{array}{ll}\mho _{\iota }(0)+\sum \limits _{\kappa =2}^{{\textbf{v}}}\Big \{\frac{23}{12}\digamma ({\tau }_{\kappa },\mho ^{\kappa })\Delta \tau -\frac{4}{3}\digamma ({\tau }_{\kappa -1},\mho ^{\kappa -1})\Delta \tau +\frac{7}{12}\digamma ({\tau }_{\kappa -2},\mho ^{\kappa -2})\Delta \tau \Big \},~~\tau \in [0,\intercal _{1}].\\ \mho _{\iota }(\intercal _{1})+\frac{(\Delta \tau )^{\omega -1}}{\Gamma (\omega +1)}\sum \limits _{\kappa =2}^{{\textbf{v}}}\digamma ({\tau }_{\kappa -2},\mho ^{\kappa -2})\widetilde{\Im _{1}}\\ \quad + \frac{(\Delta \tau )^{\omega -1}}{\Gamma (\omega +2)}\sum \limits _{\kappa =2}^{{\textbf{v}}}\Big \{\digamma ({\tau }_{\kappa -1},\mho ^{\kappa -1})-\digamma ({\tau }_{\kappa -2},\mho ^{\kappa -2})\Big \}\widetilde{\Im _{2}}\\ \quad +\frac{\omega (\Delta \tau )^{\omega -1}}{2\Gamma (\omega +3)}\sum \limits _{\kappa =2}^{{\textbf{v}}}\Big \{\digamma ({\tau }_{\kappa },\mho ^{\kappa })-2\digamma ({\tau }_{\kappa -1},\mho ^{\kappa -1})+\digamma ({\tau }_{\kappa -2},\mho ^{\kappa -2})\Big \}\widetilde{\Im _{3}},~~\tau \in [\intercal _{1},\intercal _{2}],\\ \mho _{\iota }(\intercal _{2})+\sum \limits _{\kappa ={\textbf{v}}+3}^{{\mathfrak {n}}}\Big \{\frac{7}{12}\digamma ({\tau }_{\kappa -2},\mho ^{\kappa -2})\Delta \tau -\frac{4}{3}\digamma ({\tau }_{\kappa -1},\mho ^{\kappa -1})\Delta \tau +\frac{23}{12}\digamma ({\tau }_{\kappa },\mho ^{\kappa })\Delta \tau \Big \} \\ \quad +\sum \limits _{\kappa ={\textbf{v}}+3}^{{\mathfrak {n}}}\Big \{\frac{7}{12}\big ({\mathbb {B}}({\tau }_{\kappa -1})-{\mathbb {B}}({\tau }_{\kappa -2})\big )\wp \mho ^{\kappa -2}- \frac{4}{3}\big ({\mathbb {B}}({\tau }_{\kappa })-{\mathbb {B}}({\tau }_{\kappa -1})\big )\wp \mho ^{\kappa -1}\\ \quad +\frac{23}{12}\big ({\mathbb {B}}({\tau }_{\kappa +1})-{\mathbb {B}}({\tau }_{\kappa })\big )\wp \mho ^{\kappa }\Big \},~~\tau \in [\intercal _{2},\intercal ],\end{array}\right. } \end{aligned}$$

where

94 $$\begin{aligned} \widetilde{\Im _{1}}:=({\textbf{v}}-\kappa -1)^{\omega }-({\textbf{v}}-\kappa )^{\omega }, \end{aligned}$$

95 $$\begin{aligned} \widetilde{\Im _{2}}:=({\textbf{v}}-\kappa +1)^{\omega }({\textbf{v}}-\kappa +2\omega +3)-({\textbf{v}}-\kappa )^{\omega }({\textbf{v}}-\kappa +3\omega +3), \end{aligned}$$

and

96 $$\begin{aligned} \widetilde{\Im _{3}}:={\left\{ \begin{array}{ll}({\textbf{v}}-\kappa +1)^{\omega }\Big (2({\textbf{v}}-\kappa )^{2}+ (3\omega +10)({\textbf{v}}-\kappa )+2\omega ^{2}+9\omega +12\Big )\\ \quad + ({\textbf{v}}-\kappa )^{\omega }\Big (2({\textbf{v}}-\kappa )^{2}+(5\omega +10)({\textbf{v}}-\kappa )+6\omega ^{2}+18\omega +12\Big ). \end{array}\right. } \end{aligned}$$

### Caputo–Fabrizio fractional derivative operator

The aim of this research is to examine the co-infection of the TB and COVID-19 models using integer-order (2), exponential decay kernel (4) and the ensuing stochastic scheme (28). This plan includes significant pulmonary inflammation, which means that the circulatory system is a major site of disease combat for a variety of diseases. The mathematical structure will be constructed using the classical-order formulation at first, the exponential decay memory at a later stage, and the stochastic setting in the phases that proceed in the case when $\intercal$ is selected as the ultimate dissemination time. Following this, the following numerical pattern is given to clarify this occurrence.

At this point, we examine the sequential configurations (2), (4) and (28) analytically by using the method outlined in Ref.^29^ in the context of the CFFD. In order to lay out the procedure, we did what follows:

97 $$\begin{aligned} {\left\{ \begin{array}{ll} \frac{d\mho _{\iota }(\tau )}{d\tau }=\digamma (\tau ,\mho _{\iota }).~\mho _{\iota }(0)=\mho _{\iota ,0},~\iota =1,2,...,{\mathfrak {n}}~if~\tau \in [0,\intercal _{1}],\\ \,_{\intercal _{1}}^{CF}{\textbf{D}}_{\tau }^{\omega }\mho _{\iota }(\tau )=\digamma (\tau ,\mho _{\iota }),~\mho _{\iota }(\intercal _{1})=\mho _{\iota ,1},~if~\tau \in [\intercal _{1},\intercal _{2}],\\ d\mho _{\iota }(\tau )=\digamma (\tau ,\mho _{\iota })d\tau +\wp _{\iota }\mho _{\iota }d{\mathbb {B}}_{\iota }(\tau ),~\mho _{\iota }(\intercal _{2})=\mho _{\iota ,2},~if~\tau \in [\intercal _{2},\intercal ]. \end{array}\right. } \end{aligned}$$

Thus, it implies that

98 $$\begin{aligned} \mho _{\iota }^{{\textbf{v}}}={\left\{ \begin{array}{ll}\mho _{\iota }(0)+\sum \limits _{\kappa =2}^{{\textbf{v}}}\Big \{\frac{23}{12}\digamma ({\tau }_{\kappa },\mho ^{\kappa })\Delta \tau -\frac{4}{3}\digamma ({\tau }_{\kappa -1},\mho ^{\kappa -1})\Delta \tau +\frac{7}{12}\digamma ({\tau }_{\kappa -2},\mho ^{\kappa -2})\Delta \tau \Big \},~~\tau \in [0,\intercal _{1}].\\ \mho _{\iota }(\intercal _{1})+\frac{1-\omega }{{\mathbb {M}}(\omega )}\digamma ({\tau }_{{\mathfrak {n}}},\mho ^{{\mathfrak {n}}})+\frac{\omega }{{\mathbb {M}}(\omega )}\sum \limits _{\kappa =2}^{{\textbf{v}}}\Big \{\frac{7}{12}\digamma ({\tau }_{\kappa -2},\mho ^{\kappa -2})\Delta \tau -\frac{4}{3}\digamma ({\tau }_{\kappa -1},\mho ^{\kappa -1})\Delta \tau \\ \quad +\frac{23}{12}\digamma ({\tau }_{\kappa },\mho ^{\kappa })\Delta \tau \Big \},~~\tau \in [\intercal _{1},\intercal _{2}],\\ \mho _{\iota }(\intercal _{2})+\sum \limits _{\kappa ={\textbf{v}}+3}^{{\mathfrak {n}}}\Big \{\frac{7}{12}\digamma ({\tau }_{\kappa -2},\mho ^{\kappa -2})\Delta \tau -\frac{4}{3}\digamma ({\tau }_{\kappa -1},\mho ^{\kappa -1})\Delta \tau +\frac{23}{12}\digamma ({\tau }_{\kappa },\mho ^{\kappa })\Delta \tau \Big \} \\ \quad +\sum \limits _{\kappa ={\textbf{v}}+3}^{{\mathfrak {n}}}\Big \{\frac{7}{12}\big ({\mathbb {B}}({\tau }_{\kappa -1})-{\mathbb {B}}({\tau }_{\kappa -2})\big )\wp \mho ^{\kappa -2}- \frac{4}{3}\big ({\mathbb {B}}({\tau }_{\kappa })-{\mathbb {B}}({\tau }_{\kappa -1})\big )\wp \mho ^{\kappa -1}\\ \quad +\frac{23}{12}\big ({\mathbb {B}}({\tau }_{\kappa +1})-{\mathbb {B}}({\tau }_{\kappa })\big )\wp \mho ^{\kappa }\Big \},~~\tau \in [\intercal _{2},\intercal ].\end{array}\right. } \end{aligned}$$

### Atangana–Baleanu–Caputo fractional derivative operator

The current research aims to investigate the co-infection of the stochastic technique (28) and the integer-order model (2) and the GML kernel TB and COVID-19 models (5). Significant pulmonary inflammation is present in this design, indicating that the circulatory system is a key area of illness defense for a number of illnesses. Initially, the classical-order interpretation will be used to build the computational framework; afterwards, the GML function will be implemented; and in the phases that follow, the stochastic configuration will be used in the scenario where $\intercal$ is chosen as the eventual propagation time. After that, the subsequent numerical structure is provided to explain these instances. In particular, we analyze the sequential configurations (2), (5) and (28) numerically employing the algorithm defined in Ref.^29^ in the framework of the ABCFD. In order to lay out the procedure, we did what follows:

$$\begin{aligned} {\left\{ \begin{array}{ll} \frac{d\mho _{\iota }(\tau )}{d\tau }=\digamma (\tau ,\mho _{\iota }).~\mho _{\iota }(0)=\mho _{\iota ,0},~\iota =1,2,...,{\mathfrak {n}}~if~\tau \in [0,\intercal _{1}],\\ \,_{\intercal _{1}}^{ABC}{\textbf{D}}_{\tau }^{\omega }\mho _{\iota }(\tau )=\digamma (\tau ,\mho _{\iota }),~\mho _{\iota }(\intercal _{1})=\mho _{\iota ,1},~if~\tau \in [\intercal _{1},\intercal _{2}],\\ d\mho _{\iota }(\tau )=\digamma (\tau ,\mho _{\iota })d\tau +\wp _{\iota }\mho _{\iota }d{\mathbb {B}}_{\iota }(\tau ),~\mho _{\iota }(\intercal _{2})=\mho _{\iota ,2},~if~\tau \in [\intercal _{2},\intercal ]. \end{array}\right. } \end{aligned}$$

Thus, it implies that

$$\begin{aligned} \mho _{\iota }^{{\textbf{v}}}={\left\{ \begin{array}{ll}\mho _{\iota }(0)+\sum \limits _{\kappa =2}^{{\textbf{v}}}\Big \{\frac{23}{12}\digamma ({\tau }_{\kappa },\mho ^{\kappa })\Delta \tau -\frac{4}{3}\digamma ({\tau }_{\kappa -1},\mho ^{\kappa -1})\Delta \tau +\frac{7}{12}\digamma ({\tau }_{\kappa -2},\mho ^{\kappa -2})\Delta \tau \Big \},~~\tau \in [0,\intercal _{1}].\\ \mho _{\iota }(\intercal _{1})+\frac{1-\omega }{ABC(\omega )}\digamma ({\tau }_{{\mathfrak {n}}},\mho ^{{\mathfrak {n}}})+\frac{\omega (\Delta \tau )^{\omega -1}}{ABC(\omega )\Gamma (\omega +1)}\sum \limits _{\kappa =2}^{{\textbf{v}}}\digamma ({\tau }_{\kappa -2},\mho ^{\kappa -2})\widetilde{\Im _{1}}\\ \quad + \frac{\omega (\Delta \tau )^{\omega -1}}{ABC(\omega )\Gamma (\omega +2)}\sum \limits _{\kappa =2}^{{\textbf{v}}}\Big \{\digamma ({\tau }_{\kappa -1},\mho ^{\kappa -1})-\digamma ({\tau }_{\kappa -2},\mho ^{\kappa -2})\Big \}\widetilde{\Im _{2}}\\ \quad +\frac{\omega (\Delta \tau )^{\omega -1}}{2ABC(\omega )\Gamma (\omega +3)}\sum \limits _{\kappa =2}^{{\textbf{v}}}\Big \{\digamma ({\tau }_{\kappa },\mho ^{\kappa })-2\digamma ({\tau }_{\kappa -1},\mho ^{\kappa -1})+\digamma ({\tau }_{\kappa -2},\mho ^{\kappa -2})\Big \}\widetilde{\Im _{3}},~~\tau \in [\intercal _{1},\intercal _{2}],\\ \mho _{\iota }(\intercal _{2})+\sum \limits _{\kappa ={\textbf{v}}+3}^{{\mathfrak {n}}}\Big \{\frac{7}{12}\digamma ({\tau }_{\kappa -2},\mho ^{\kappa -2})\Delta \tau -\frac{4}{3}\digamma ({\tau }_{\kappa -1},\mho ^{\kappa -1})\Delta \tau +\frac{23}{12}\digamma ({\tau }_{\kappa },\mho ^{\kappa })\Delta \tau \Big \} \\ \quad +\sum \limits _{\kappa ={\textbf{v}}+3}^{{\mathfrak {n}}}\Big \{\frac{7}{12}\big ({\mathbb {B}}({\tau }_{\kappa -1})-{\mathbb {B}}({\tau }_{\kappa -2})\big )\wp \mho ^{\kappa -2}- \frac{4}{3}\big ({\mathbb {B}}({\tau }_{\kappa })-{\mathbb {B}}({\tau }_{\kappa -1})\big )\wp \mho ^{\kappa -1}\\ \quad +\frac{23}{12}\big ({\mathbb {B}}({\tau }_{\kappa +1})-{\mathbb {B}}({\tau }_{\kappa })\big )\wp \mho ^{\kappa }\Big \},~~\tau \in [\intercal _{2},\intercal ],\end{array}\right. } \end{aligned}$$

where the previous values of $\widetilde{\Im _{1}}, ~\widetilde{\Im _{2}}$, and $\widetilde{\Im _{3}}$ are found in (94)–(96).

### Experimental outcomes and discussion

In order to support research ideas, we will demonstrate mathematical simulation techniques in the next part that make leverage of the Atangana and Araz approaches formerly mentioned in Ref.^29^. The appropriateness and usefulness of the planned TB-COVID-19 are demonstrated through a number of concrete instances, such as manpower reductions, delays in test result transformation, and limitations of analytical equipment. The accessibility and promptness of TB examinations have been severely impacted by these interruptions in the deterministic-probabilistic situation. Utilizing MATLAB 21, all quantitative and symbolic computations were performed.

Researchers are at present demonstrating a great deal of enthusiasm in the estimation of modeling characteristics from provided statistical information, and it is thought to be an essential component of quantitative disease investigations. The aforementioned section was added to the current investigation employing the popular nonlinear least squares method. Applying the previously described method, the settings were determined, and the structure was calibrated to actual codynamic situations found in Later research from the Philippines and South Africa revealed that, for a specific duration, COVID-19 patients having TB had a 2.17^53^ and 2.7^54^ worse probability of death, respectively, than COVID-19 individuals lacking TB^53^. Especially, the entire number of documented infections and fatalities over the time span between March 2020 (the initial incidence had been identified on March 12, 2020) and June 2022 were used to determine the characteristics of the model. Considering the implementation of (99), the Ordinary Least Square solution was employed to reduce the inaccuracy concepts, and the associated relative deviation is incorporated in assessing the quality of fit as

99 $$\begin{aligned} \min \left\{ \frac{\sum \limits _{\Bbbk =1}^{{\mathfrak {n}}}(\Im _{\Bbbk }-{\hat{\Im }}_{\Bbbk })^{2}}{\sum \limits _{\Bbbk =1}^{{\mathfrak {n}}}\Im ^{2}_{\Bbbk }}\right\} . \end{aligned}$$

**Figure 6:**
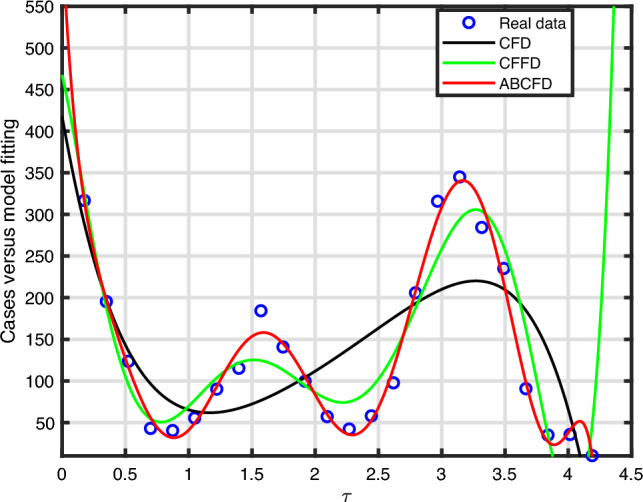
The codynamics of TB-COVID-19 fitting outcomes considering the data obtained from WHO^55^ for weekly reported cases.

The documented accumulative infection rates are denoted by $\Im _{\Bbbk }$ in this particular instance, while the total number of contaminated occurrences determined by modeling execution is denoted by ${\hat{\Im }}_{\Bbbk }$. The people who are moved daily from the contaminated compartment to the confined compartment are added together to determine the estimated levels of progressive transmission. With the exception of $\varsigma _{1}=0.0456$, which is envisioned, all the parameters are estimates. When $\tau =1$ and $\omega =1$ the data in Fig. 6 has been fitted to the model.

The parameters’ projected estimates are displayed in Table 2.

**Table 2:** Details on the system’s characteristic.

Notations	Values	References
$\nabla$	500	Supposed
$\beta$	0.0477	^53^
$\alpha _{1}$	0.6	^53^
$\alpha _{2}$	0.659	^54^
$\varphi _{1}$	0.02	Supposed
$\varsigma _{3}$	0.01	Estimated
$\zeta _{{\textbf{C}}}$	0.023	^54^
$\zeta _{{\textbf{T}}}$	0.01	^54^
$\varphi _{3}$	0.05	Calculated
$\lambda$	0.03	Calculated
$\epsilon$	0.03	Calculated
$\xi$	0.003	Calculated
$\rho$	0.021	Supposed
$\varpi$	0.09	^53^
$\epsilon$	0.048	Estimated
$\mu$	0.25	^54^
$\eta$	0.01	Estimated
$\nu$	0.002	Estimated
$\zeta _{{\textbf{T}}{\textbf{C}}}$	0.2	Assumed
$\varphi _{2}$	0.05	^53^
$\delta$	0.056	Estimated
$\theta _{2}$	0.95	Estimated
$\theta _{1}$	0.9	Estimated
$\varsigma _{2}$	0.25	^53^

#### Example 1

To illustrate our results, we quantitatively generate the paths for the probabilistic sickness structures (2), (3) and (28) and their corresponding deterministic components. The starting points are $({{\textbf{S}}},{{\textbf{L}}}_{{\textbf{T}}},{{\textbf{I}}}_{{\textbf{T}}},{{\textbf{E}}}_{{\textbf{C}}},{{\textbf{I}}}_{{\textbf{C}}},{{\textbf{L}}}_{{\textbf{T}}{\textbf{C}}},{{\textbf{I}}}_{{\textbf{T}}{\textbf{C}}},{{\textbf{R}}})(0)=(1000,100,10,1000,10,7,5,1)$ and the time range is [0, 100] units. Table 2 allows us to re-select the parameters to represent the piecewise methodology assessment of the naturally occurring factor process for (2), (3) and (28), respectively.

Here, we calculate the fundamental reproductive quantity ${\mathbb {R}}_{0}=2.4563>1$ for the deterministic framework (2), which suggests that co-infection of TB and COVID-19 will continue to exist in the average situation in both submodels. To observe how noise concentration affects the behavior of the probabilistic framework (28), we select random perturbations $\wp _{\jmath }=0.03~(\jmath =1,...,5).$ This yields ${\mathbb {R}}_{0}^{{\mathbb {S}}}=\frac{\psi _{{\textbf{C}}}\widetilde{{\textbf{S}}}}{{(\beta +\epsilon \psi _{{\textbf{T}}}+\varphi _{1}+\varphi _{2})}+\frac{\wp _{4}^{2}}{2}}=1.231>1.$ The existence of an ESD for the probabilistic model (28) is demonstrated by Theorem 8.

**Figure 7:**
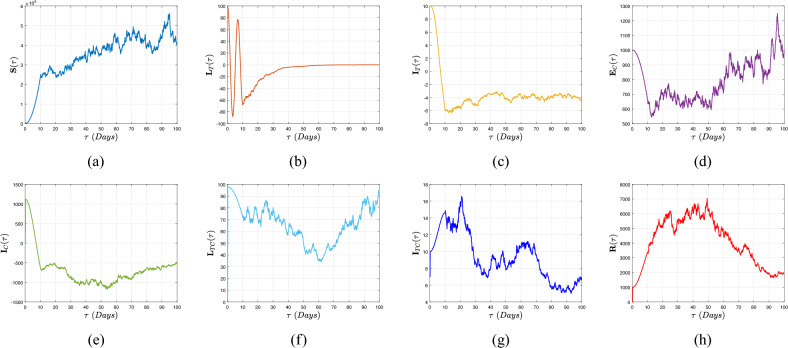
Time evaluation plots for deterministic-probabilistic co-infection TB-COVID-19 models (2), (3) and (28) with the impacts of latent and active TB outbreaks using CFD operator having FO $\omega =0.98,$ low intensities and ICs (1000, 100, 10, 1000, 10, 7, 5, 1).

**Figure 8:**
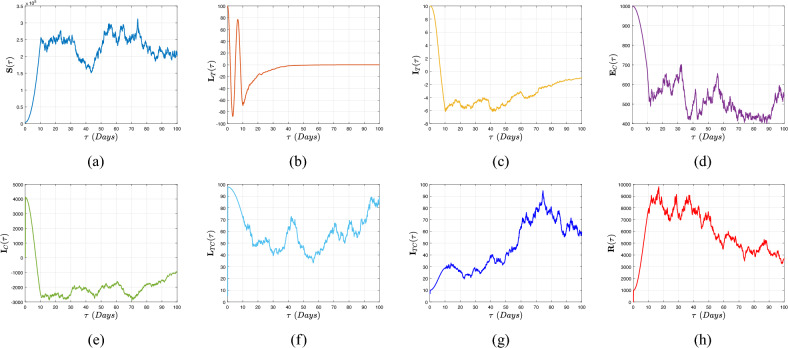
Time evaluation plots for deterministic-probabilistic co-infection TB-COVID-19 models (2), (3) and (28) with the impacts of latent and active TB outbreaks using CFD operator having FO $\omega =0.98,$ low intensities and ICs (4000, 100, 40, 1000, 10, 7, 5, 4).

**Figure 9:**
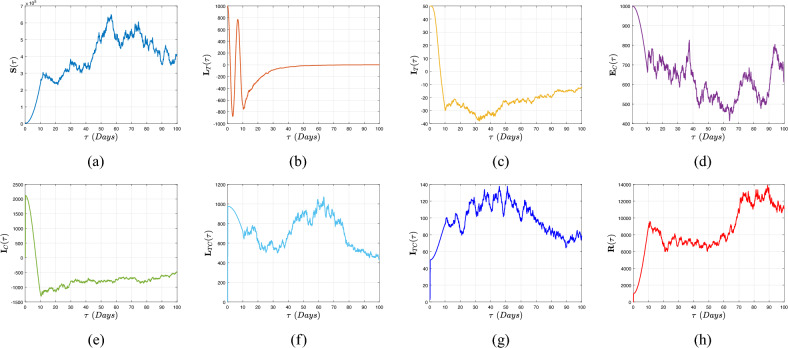
Time evaluation plots for deterministic-probabilistic co-infection TB-COVID-19 models (2), (3) and (28) with the impacts of latent and active TB outbreaks using CFD operator having FO $\omega =0.98,$ low intensities and ICs (5000, 100, 50, 1000, 10, 7, 5, 5).

When a power-law-type kernel with a FO $\omega =0.98$ is employed on (3), Fig. 7a–h illustrates how incorporating two propagation interprets enhances the occurrence of ailments in comparison to a single procedure. In view of the CFD operator and biological-nature strategy, we also find that certain combinations prove more deadly than others. A transmission surge is produced by all interactions comprising the effective collaboration rate connecting ${{\textbf{S}}}$ and ${{\textbf{I}}}_{{\textbf{T}}}$. This is followed by any coupling via the effective interface rate between ${\textbf{S}}$ and ${{\textbf{I}}}_{{\textbf{T}}{\textbf{C}}}$, and finally other procedures. The respective two ICs $({{\textbf{S}}},{{\textbf{L}}}_{{\textbf{T}}},{{\textbf{I}}}_{{\textbf{T}}},{{\textbf{E}}}_{{\textbf{C}}},{{\textbf{I}}}_{{\textbf{C}}},{{\textbf{L}}}_{{\textbf{T}}{\textbf{C}}},{{\textbf{I}}}_{{\textbf{T}}{\textbf{C}}},{{\textbf{R}}})(0)=(4000,100,40,1000,10,7,5,4)$ and $({{\textbf{S}}},{{\textbf{L}}}_{{\textbf{T}}},{{\textbf{I}}}_{{\textbf{T}}},{{\textbf{E}}}_{{\textbf{C}}},{{\textbf{I}}}_{{\textbf{C}}},{{\textbf{L}}}_{{\textbf{T}}{\textbf{C}}},{{\textbf{I}}}_{{\textbf{T}}{\textbf{C}}},{{\textbf{R}}})(0)=(5000,100,50,1000,10,7,5,5),$ are depicted in Figs. 8a–h and 9a–h, with various population schemes.

**Figure 10:**
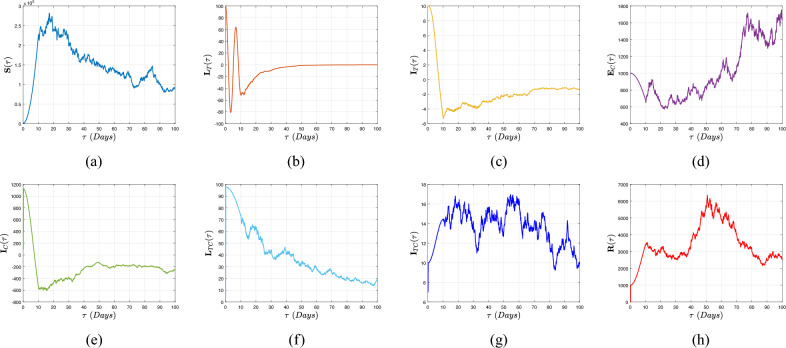
Time evaluation plots for deterministic-probabilistic co-infection TB-COVID-19 models (2), (3) and (28) with the impacts of latent and active TB outbreaks using CFFD operator having FO $\omega =0.98,$ low intensities and ICs (1000, 100, 10, 1000, 10, 7, 5, 1).

**Figure 11:**
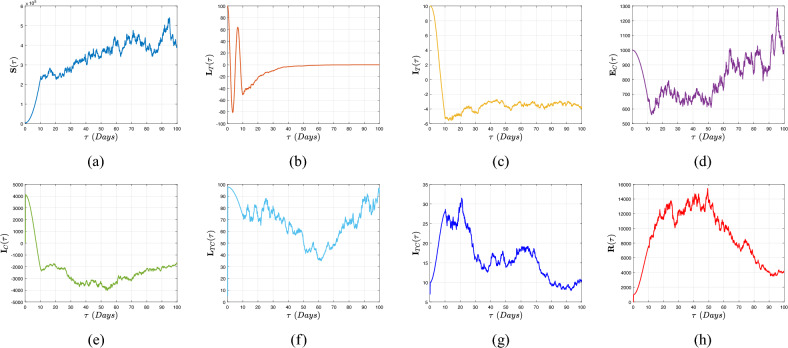
Time evaluation plots for deterministic-probabilistic co-infection TB-COVID-19 models (2), (4) and (28) with the impacts of latent and active TB outbreaks using CFFD operator having FO $\omega =0.98,$ low intensities and ICs (4000, 100, 40, 1000, 10, 7, 5, 4).

**Figure 12:**
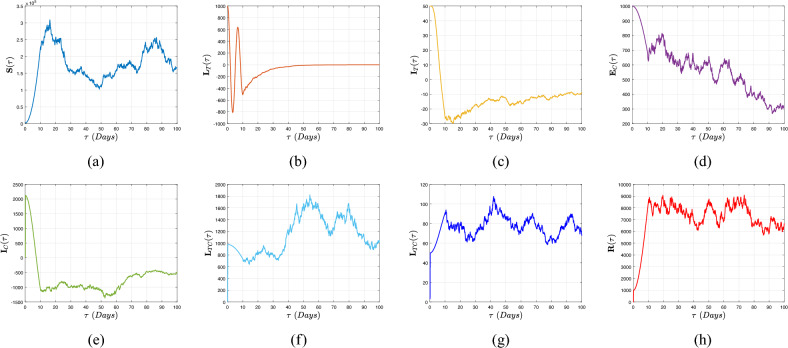
Time evaluation plots for deterministic-probabilistic co-infection TB-COVID-19 models (2), (4) and (28) with the impacts of latent and active TB outbreaks using CFFD operator having FO $\omega =0.98,$ low intensities and ICs (5000, 100, 50, 1000, 10, 7, 5, 5).

We employ the identical factors as in (3), when implementing the identical methodology to the CFFD operator of DEs (2), (4) and (28), respectively. However, we modify the ICs as previously mentioned. It is simple to compute the threshold factors ${\mathbb {R}}_{0}^{{\mathbb {S}}}>1$ and ${\mathbb {R}}_{0}^{{\mathbb {C}}}<1.$ As seen in Fig. 10a–h, the co-infections are expected to continue in a typical way, supporting the result of Theorem 8 (see Figs. 11a–h, 12a–h). According to this research, co-infection will spread throughout the body and develop ineffective causative agents, whereas mycobacterium TB will go obsolete.

**Figure 13:**
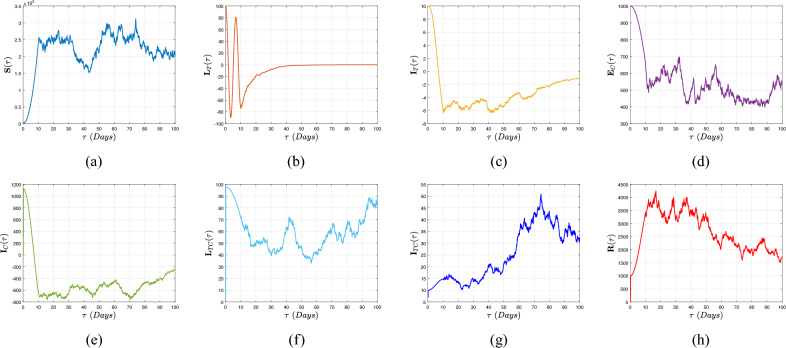
Time evaluation plots for deterministic-probabilistic co-infection TB-COVID-19 models (2), (5) and (28) with the impacts of latent and active TB outbreaks using ABCFD operator having FO $\omega =0.98,$ low intensities and ICs (1000, 100, 10, 1000, 10, 7, 5, 1).

**Figure 14:**
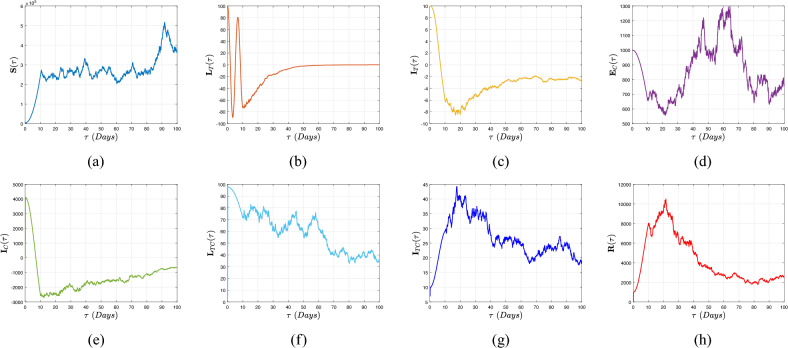
Time evaluation plots for deterministic-probabilistic co-infection TB-COVID-19 models (2), (5) and (28) with the impacts of latent and active TB outbreaks using ABCFD operator having FO $\omega =0.98,$ low intensities and ICs (4000, 100, 40, 1000, 10, 7, 5, 4).

**Figure 15:**
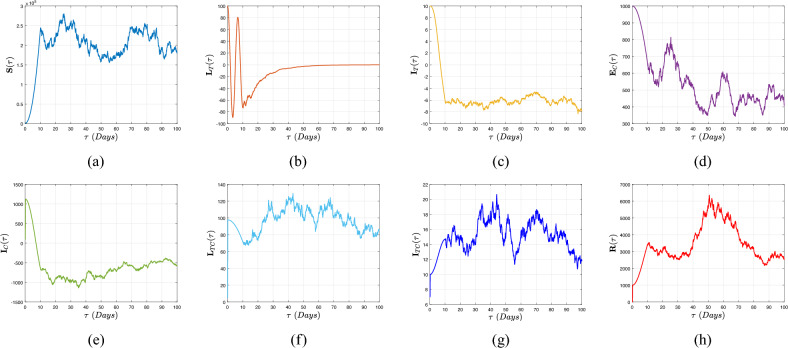
Time evaluation plots for deterministic-probabilistic co-infection TB-COVID-19 models (2), (5) and (28) with the impacts of latent and active TB outbreaks using ABCFD operator having FO $\omega =0.98,$ low intensities and ICs (5000, 100, 50, 1000, 10, 7, 5, 5).

In light of the prevalent concentrations and parameterization fluctuations discussed above, an intervention plan based on the computational findings for (2), (5) and (28) seems to be effective. There is an ESD of a probabilistic framework (28), as shown by Theorems 8. These results suggest that co-infection will become increasingly permanent while TB will go extinct for ABCFD case. These are corroborated by Figs. 13a–h, 14a–h and 15a–h, respectively.

#### Example 2

For probabilistic co-infection systems (28) involving community propagation, it is challenging to define appropriate criteria for virus extermination considering the limits of statistical approaches. Nonetheless, we provide a numerical model of the disappearance of illnesses where the noise is high for a thorough explanation. For instance, in the actual environment, individuals haphazardly raise vaccination or exterminating rates to stop co-infection from spreading. This successfully removes contamination.

**Figure 16:**
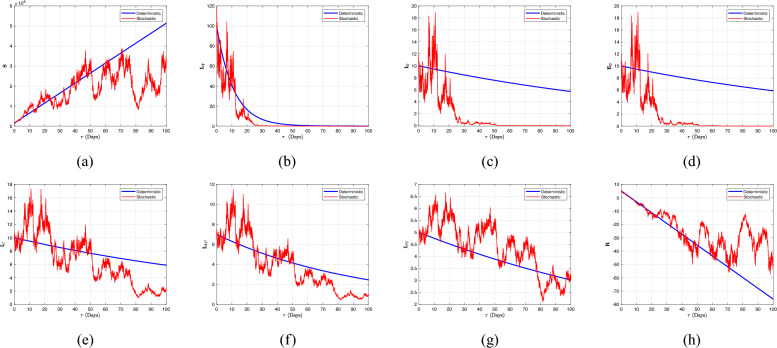
Time evaluation plots for deterministic-probabilistic co-infection TB-COVID-19 model (2) and (28) with the impacts of latent and active TB outbreaks with low intensities and ICs (5000, 100, 50, 1000, 10, 7, 5, 5) using CFD when $\omega =0.95$.

To illustrate that high levels of environmental disturbance will eventually cause TB to disappear, we set $\wp _{1}=\wp _{4}=0.21,$ $\wp _{\kappa }=0.11,~\kappa =2,3,5,6,7$ with the identical setting off rate as well as additional factors as in the aforesaid discussion. Following this, as Fig. 16a–h illustrates, co-infection will become extinct.

#### Example 3

For the probabilistic COVID-19 model in the absence of TB (75), the white noise $\wp _{\jmath }=0.02,~(\jmath =1,...,8)$ and the IC and the remaining arguments are the similar as in Example 1. Thus, we determine ${\mathcal {R}}_{0}^{\kappa }=2.83212>1.$ and the quasi-equilibrium $({{\textbf{S}}}_{\jmath }^{*},{{{\textbf{E}}}_{{\textbf{C}}}}_{\jmath }^{*},{{{\textbf{I}}}_{{\textbf{T}}}}_{\jmath }^{*},{{\textbf{R}}}_{\jmath }^{*})=(801867.49, 6698439.45, 10.768934.65, 1387430.65).$ In view of Theorem 11.

100 $$\begin{aligned} \Pi =\begin{pmatrix} 0.0011&{}0.0003&{}-0.00055&{}0.0003\\ 0.0003&{}0.0072&{}0.007&{}0.0033\\ -0.00061&{}0.0070&{}0.0078&{}0.0026\\ 0.003&{}0.0033&{}0.0026&{}0.0022 \end{pmatrix}. \end{aligned}$$

As a result, the following is the relevant P.D.F ${\mathcal {U}}({\tilde{\Psi }})=\frac{1}{4\varphi _{2}^{2}}\vert \Pi \vert ^{-1/2}\exp \Big (\frac{-1}{2}{\tilde{\Psi }}\Pi ^{-1}{\tilde{\Psi }}^{{\textbf{T}}}\Big ).$ where

$$\begin{aligned} {\tilde{\Psi }}=\Big (\ln \frac{{{\textbf{S}}}_{\jmath }}{801867.49},\ln \frac{{{{\textbf{E}}}_{{\textbf{C}}}}_{\jmath }}{6698439.45},\ln \frac{{{{\textbf{I}}}_{{\textbf{T}}}}_{\jmath }}{10.768934.65},\ln \frac{{{{\textbf{R}}}}_{\jmath }}{1387430.65}\Big ). \end{aligned}$$

Consequently, the four marginal D. Fs of ${\tilde{\Psi }}$ are as follows:

$$\begin{aligned}{} & {} \frac{\partial {\mathcal {U}}}{\partial {{\textbf{S}}}_{\jmath }}=10.34267\exp \big (-420.4235(\ln {{\textbf{S}}}_{\jmath }-17.1893)\big ),~~~~\frac{\partial {\mathcal {U}}}{\partial {{{\textbf{E}}}_{{\textbf{C}}}}_{\jmath }}=3.75123\exp \big (-70.8992(\ln {{{\textbf{E}}}_{{\textbf{C}}}}_{\jmath }-8.7834)\big ), \nonumber \\{} & {} \frac{\partial {\mathcal {U}}}{\partial {{\textbf{I}}}_{\jmath }}=3.3410\exp \big (-80.2341(\ln {{\textbf{I}}}_{\jmath }-12.00345)\big ),~~~~\frac{\partial {\mathcal {U}}}{\partial {{{\textbf{R}}}}_{\jmath }}=9.2301\exp \big (-235.9921(\ln {{{\textbf{R}}}}_{\jmath }-5.5512)\big ). \end{aligned}$$

**Figure 17:**
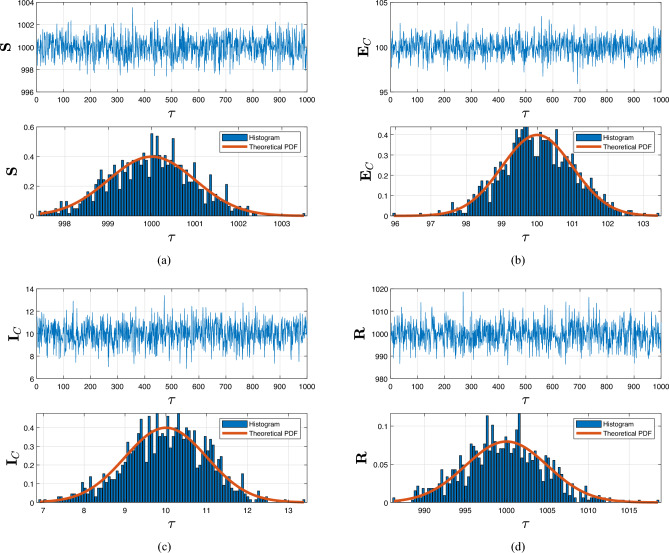
Numerical modeling of the outcome $\big ({\textbf{S}}(\tau ),{{\textbf{E}}}_{{\textbf{C}}}(\tau ),{{\textbf{I}}}_{{\textbf{C}}}(\tau ),{{\textbf{R}}}(\tau )\big )$ in system (75) is displayed in the upper portion row. The P.D.Fs and marginal D.Fs of ${\textbf{S}},{{\textbf{E}}}_{{\textbf{C}}},{{\textbf{I}}}_{{\textbf{C}}}$ and ${{\textbf{R}}}$ are displayed in the lower portion row, respectively, with $\wp _{\jmath }=0.02~(\jmath =1,...,8)$ and ${\mathcal {R}}_{0}^{\kappa }=2.83212>1$.

Finally, population concentrations oscillate according to the quasi-stable equilibrium ${\textbf{U}}^{*}$, as seen in Fig. 17.

## Conclusions

In this article, a deterministic-stochastic model is being suggested to investigate the potential transmission of the codynamics of COVID-19 and TB. Taking into account the deterministic fractional model and stochastic approach, we have provided the qualitative characteristics such as positivity and boundedness, reproduction number and their allied outcomes for co-infection model (2), global positive solution and unique erogdicity for the co-dynamics of (28). Besides that, applying the Khasminskii notion and a suitable Lyapunov function, the existence of a stationary distribution in model (28) was analytically verified. Additionally, an accurate representation of the P.D.F regarding a quasi-equilibrium point of the random-perturbed COVID-19 model constitutes one of this research’s particularly noteworthy discoveries. In fact, it has been determined that the validity and strength of our numerical outcomes and modeled estimates have been provided in a piecewise fractional DEs context. Furthermore, the outcomes of this study shed a spotlight on the P.D.F and stationary distribution of the probabilistic multidimensional framework at its quasi-equilibrium point. Although the ABCFD, CFFD and CFD have been demonstrated to be efficient in documenting various interaction practices, we contend that their ability to accomplish this effectively may be hindered by the vastness of biological systems. It follows that oscillation might eliminate signals that are widely dispersed, despite leaving infectious diseases uncontrolled.

Predicting how TB is propagated by population mobility and random disturbances was challenging until this research was conducted. The research advances our knowledge of why TB still exists around the globe. Regarding stochastic TB systems, including community propagation, it is challenging to define adequate requirements for infection eradication considering the restrictive nature of computational approaches. On the other hand, we also offer a simulation of the disease’s disappearance. To determine the necessary requirements for TB’s endurance and extermination, additional investigation needs to be performed.

Numerous fascinating and open-ended, high-dimensional models deserve further consideration. It is vital for inquiry into phenomena that are impacted by additional factors, such as neural networking with stochastic resonance or oscillatory spectrum disruption, while examining the unpredictable nature of this form of contention. Such studies may include certain specific but complex concepts, including evaluating the effects of Lévy and Poisson noise or Markov processes. These pertinent issues might be covered in the upcoming analysis.

## Data Availability

The datasets used and/or analyzed during the current study available from the corresponding author on reasonable request.
